# Glutamine metabolism-related genes and immunotherapy in nonspecific orbital inflammation were validated using bioinformatics and machine learning

**DOI:** 10.1186/s12864-023-09946-6

**Published:** 2024-01-17

**Authors:** Zixuan Wu, Na Li, Yuan Gao, Liyuan Cao, Xiaolei Yao, Qinghua Peng

**Affiliations:** 1 Hunan University of Traditional Chinese Medicine, Changsha, 410208 Hunan Province China; 2 Dongying People’s Hospital (Dongying Hospital of Shandong Provincial Hospital Group), Dongying, Shandong 257091 People’s Republic of China; 3 Department of Ophthalmology, the First Affiliated Hospital of Hunan University of Traditional Chinese Medicine, Changsha, 410007 Hunan Province China

**Keywords:** Nonspecific orbital inflammation (NSOI), Gln-Metabolism genes (GlnMgs), Lasso regression, SVM-RFE, Bioinformatics

## Abstract

**Background:**

Nonspecific orbital inflammation (NSOI) is an idiopathic, persistent, and proliferative inflammatory condition affecting the orbit, characterized by polymorphous lymphoid infiltration. Its pathogenesis and progression have been linked to imbalances in tumor metabolic pathways, with glutamine (Gln) metabolism emerging as a critical aspect in cancer. Metabolic reprogramming is known to influence clinical outcomes in various malignancies. However, comprehensive research on glutamine metabolism's significance in NSOI is lacking.

**Methods:**

This study conducted a bioinformatics analysis to identify and validate potential glutamine-related molecules (GlnMgs) associated with NSOI. The discovery of GlnMgs involved the intersection of differential expression analysis with a set of 42 candidate GlnMgs. The biological functions and pathways of the identified GlnMgs were analyzed using GSEA and GSVA. Lasso regression and SVM-RFE methods identified hub genes and assessed the diagnostic efficacy of fourteen GlnMgs in NSOI. The correlation between hub GlnMgs and clinical characteristics was also examined. The expression levels of the fourteen GlnMgs were validated using datasets GSE58331 and GSE105149.

**Results:**

Fourteen GlnMgs related to NSOI were identified, including FTCD, CPS1, CTPS1, NAGS, DDAH2, PHGDH, GGT1, GCLM, GLUD1, ART4, AADAT, ASNSD1, SLC38A1, and GFPT2. Biological function analysis indicated their involvement in responses to extracellular stimulus, mitochondrial matrix, and lipid transport. The diagnostic performance of these GlnMgs in distinguishing NSOI showed promising results.

**Conclusions:**

This study successfully identified fourteen GlnMgs associated with NSOI, providing insights into potential novel biomarkers for NSOI and avenues for monitoring disease progression.

**Supplementary Information:**

The online version contains supplementary material available at 10.1186/s12864-023-09946-6.

## Introduction

Non-specific orbital inflammation (NSOI) is a benign inflammatory condition of the orbit that lacks a specific infectious or local etiology. It accounts for 6%-16% of all ocular lesions and 11% of orbital malignancies [1, 2]. NSOI predominantly affects middle-aged individuals, particularly women. The precise pathophysiological basis of NSOI remains uncertain. Some studies have suggested associations with Streptococcal pharyngitis, viral upper respiratory infection, or other autoimmune conditions such as rheumatologic disease, multifocal fibrosis, and Crohn's disease [3, 4]. NSOI manifests a spectrum of clinical presentations, ranging from lacrimal gland inflammation, known as dacryoadenitis, to myositis affecting one or several extraocular muscles, alongside a cadre of other less typical symptoms [5]. Systemic corticosteroids, by virtue of empirical evidence, are entrenched as the standard therapeutic recourse, yet their prolonged administration is marred by a well-documented profile of adverse effects [6]. Evenwith efficacious corticosteroid interventions, the propensity for recurrence eclipses 50%, underscoring a pressing need for enhanced understanding of the molecular intricacies of NSOI [7]. Such insights hold the key to forging novel therapeutic avenues, which are pivotal for staving off relapse and enhancing the prognostic landscape for patients. This imperative drives the scientific quest to elucidate the underpinnings of NSOI, a quest that promises to recalibrate the management paradigms for this enigmatic condition.

Metabolic processes are the cornerstone of vitality and proliferation within the biosphere, underpinning the survival and propagation of all biotic entities. In the realm of oncology, the concept of metabolic reprogramming has emerged as a critical facilitator of neoplastic cell proliferation and resilience. Contemporary insights reveal that oncogenic transformation precipitates the genesis of discrete metabolic signatures within tumorous cells, which in turn exerts profound modulatory effects on the tumor microenvironment (TME). The TME represents a labyrinthine ecosystem, comprising a diverse array of cellular entities. This environment is frequently marred by suboptimal oxygen and nutrient availability, a consequence of an aberrant or underdeveloped vascular network [8]. With the advent of refined scientific comprehension, the significance of the infiltrating non-malignant immune contingents within the TME has been cast into the spotlight, heralding a new paradigm in cancer research. Accruing evidence underscores a deep entanglement between the immune response and salient shifts in tissue metabolism, encompassing nutrient scarcity, augmented oxygen consumption, and the biogenesis of reactive nitrogen and oxygen species [9]. Concurrently, a plethora of microenvironmental factors are known to exert influence over the maturation and functional orientation of immune cells. This intricate interplay alludes to the potential of metabolic modulation as a strategic axis to potentiate the therapeutic efficacy of immune-based interventions, presenting a frontier of translational significance in the ongoing crusade against cancer [10].

Glutamine (Gln), being the most abundant circulating amino acid, is rapidly taken up by cultured tumor cells. It plays a crucial role in cellular aerobic glycolysis by supporting tricarboxylic acid (TCA) cycle flux or serving as a citrate source for reductive carboxylation in lipid synthesis. Moreover, glutaminolysis contributes to cell survival by reducing oxidative stress and maintaining mitochondrial membrane integrity [11]. M2 macrophages heavily rely on Gln metabolism, while reduced Gln metabolism can shift macrophages towards the pro-inflammatory M1 phenotype [12]. Thus, targeting Gln metabolism may potentially reprogram tumor-associated macrophages from M2 to M1, thereby enhancing the anti-tumor inflammatory immune response. Gln metabolism also impacts Th1 cell differentiation and effector T cell activation, suggesting that modulating Gln metabolism could reshape the tumor microenvironment (TME) and improve the efficacy of immunotherapies. Alzheimer’s disease is typified by the aberrant assembly of inflammasomes-substantial multiprotein structures convened by specific pattern recognition receptors. These inflammasomes orchestrate the formation of membrane breaches and catalyze the maturation of proinflammatory cytokines, culminating in pyroptosis-a fiery and inflammatory demise of cells. While orchestrating a strategy that simultaneously targets Gln metabolism and immunotherapy emerges as a beacon of hope for NSOI, the intricacies of Gln metabolism within the scope of immune recognition and immunotherapeutic interventions remain enigmatic [13]. This study embarked on a methodical appraisal of GlnMgs and their interplay with immunotherapy in the NSOI context. The resulting insights delineate a novel therapeutic vista targeting purinosome biogenesis and Gln metabolic pathways. Despite these advances, the explicit role of Gln metabolism in the modulation of immunogenicity and the orchestration of immunotherapy for NSOI warrants a more granular probe. Therefore, we undertook this investigation to furnish a holistic assessment of GlnMgs and their nexus with immunotherapy in NSOI, a pursuit poised to unravel novel corridors for clinical innovation.

**Fig. 1:**
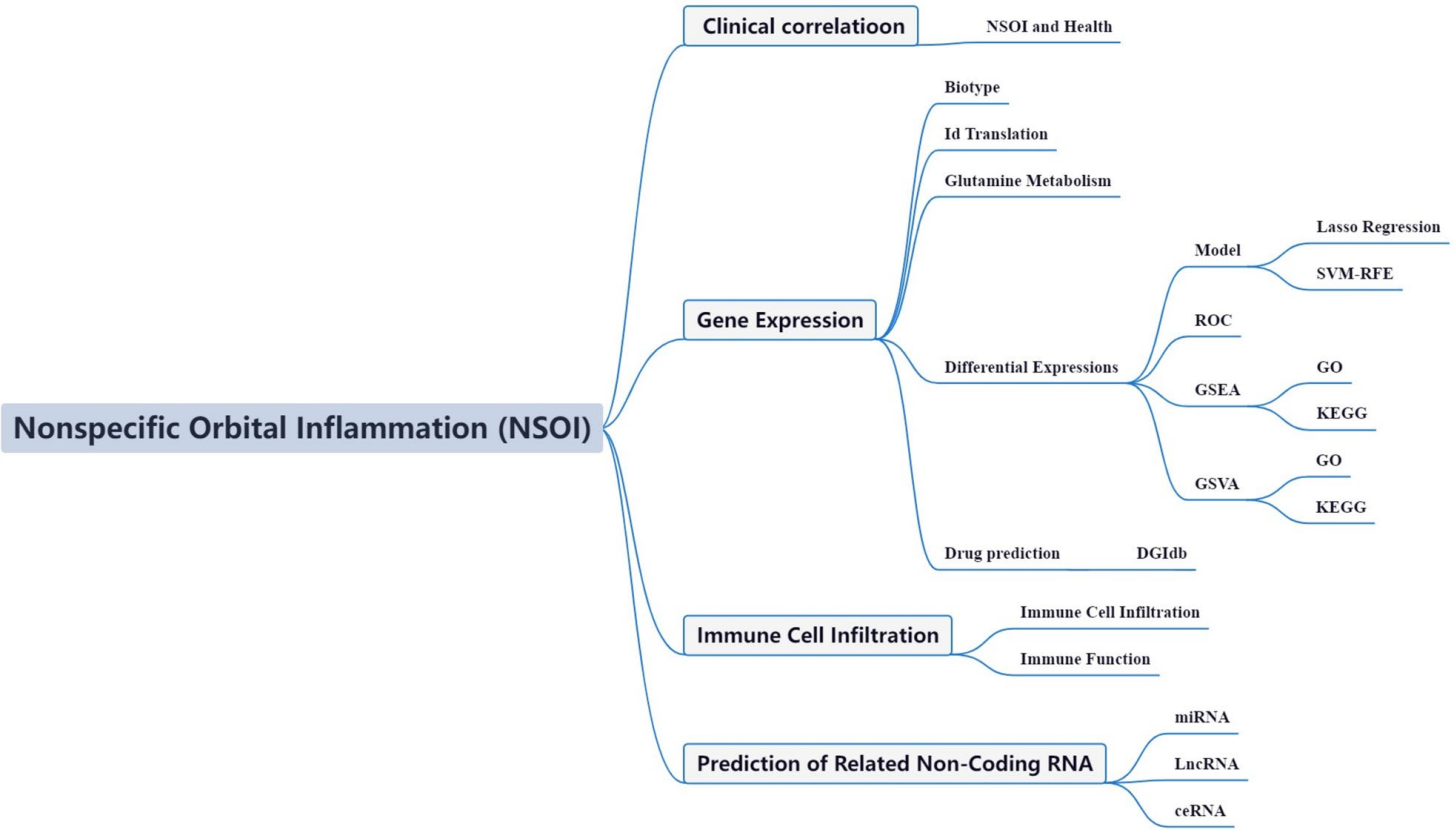
Framework. The data of NSOI patients were obtained from GEO databases, and then the GlnMgs were matched to carry out difference analysis and risk model construction, respectively. GSE132903 was used as the main body and GSE63060 was used to verify the model with good grouping, and GlnMgs related to the prognosis of NSOI patients were obtained. Then, GO, KEGG and GSEA analyses were performed with multiple databases to obtain the functions related to GlnMgs. Last, the immune cells, function and RNA changes were analyzed

The integration of bioinformatics methodologies with the prowess of high-throughput [14]. data analytics has been a cornerstone in the dissection of functional genomic constellations across a spectrum of disease paradigms, providing a fertile ground for elucidating complex molecular mechanisms [15]. The advent of expansive transcriptomic sequencing repositories, coupled with the clinical delineations proffered by the NSOI Initiative, affords an unprecedented opportunity to interrogate the transcriptional alterations and the interlinked molecular cascades germane to NSOI. The deployment of such bioinformatic explorations has rendered new vistas into the pathobiological intricacies and the foundational mechanisms operative in NSOI, offering a multi-dimensional understanding of its nature [16, 17]. Despite the rich potential of such approaches, the involvement of GlnMgs within the context of NSOI has yet to be systematically scrutinized through the bioinformatics lens. Anchoring on this gap, the present study endeavored to interrogate the NSOI-related GEO dataset with a focused aperture on GlnMgs. The aim was to illuminate their conjectural involvement in NSOI, enriching the existing compendium of knowledge as delineated in Fig. 1 of our exposition.

## Materials and methods

The methodologies proposed by Zi-Xuan Wu et al. in 2023 were employed in this study [18]. NSOI patients' data were gathered from GEO databases, and the GlnMgs were then matched to perform difference analysis and risk model creation, respectively. GSE132903 was utilized as the main body, while GSE63060 was used to validate the model with excellent grouping, and GlnMgs associated with NSOI patient prognosis were obtained. The functions associated with GlnMgs were then identified using GO, KEGG, and GSEA studies on numerous databases. Finally, immune cells, function, and RNA alterations were investigated.

### Raw data

GEO was searched for mRNA expression. Series: GSE58331 and GSE105149. Platform: GPL570-55999. GSE58331 and GSE105149 were used as the trian and test groups respectively. Strategy for searching ('eye' [MeSH] mRNA [All Fields] and normal) AND ('Homo sapiens' [Organism] AND 'Non-coding RNA profiling by array' [Filter]). A total of 79 GlnMgs were included from the MSigDB (Table S1).

### Analysis of Differentially Expressed Genes (DEGs)

Perl (https://github.com/Perl) matched and sorted transcription data to acquire exact mRNA data. The IDs were converted into gene names. After the data standardization of GSE58331 using the normalize Between Arrays function in the “limma” package, PCA was conducted by using the “factoextra” package. The Differentially expressed genes (DEGs) between NSOI and controls were analyzed. The DEGs were screened with the criteria of |Fold2FC|>1 and *p*<0.05. To show significantly deregulated genes, a heat map was created using ggplot2 and the "ComplexHeatmap" package. Pearson's correlation coefficient was employed to analyze the statistically significant and highly correlated genes within modules using the correlation analysis provided by the corrplot package.

### GO and KEGG Analysis

The biological pathways associated with the DEGs were then examined using Gene Ontology (GO). Biological processes (BP), molecular functions (MF), and cellular components (CC) controlled by the differentially expressed genes participating in autophagy were further investigated using R software, clusterProfiler, org.Hs.eg.db, enrichplot, and ggplot2 package based on KEGG data.

### Model construction and analysis of immune cell infiltration

For model construction, the glmnet package was employed for Lasso regression analysis along with cross-validation. Additionally, the support vector machine recursive feature elimination algorithm (SVM-RFE) was utilized to build a machine learning model using the e1071 package. SVMs epitomize a class of generalized linear classifiers that operate under the paradigm of supervised learning, with the primary objective of executing binary classification on datasets. The architecture of SVMs incorporates the hinge loss function as a computational tool to quantify empirical risk. In a quest for optimizing structural risk, SVMs employ regularization terms within their resolution framework, thereby endowing these classifiers with inherent sparsity and robustness. Moreover, SVMs are adept at transcending the linearity barrier by leveraging kernel methods, situating them prominently within the sphere of kernel learning methodologies. Cross-validation was used to assess the model's error and accuracy. Furthermore, the significance of the feature genes was ranked using the Lasso and SVM models. Immune cell composition was analyzed using the CIBERSORT method.

### GSEA and GSVA

GSEA and GSVA was used to find related functions and pathway variations in several samples, and Perl were used to import information. The associated score and graphs were wont to verify whether the functions and routes within the numerous Risk groups were dynamic. Every sample was classified as 'H' or 'L' depending on whether it had been a high-risk cluster of prognosis-related GlnMgs. The associated scores and visualizations were employed to assess the dynamic activities and pathways within different risk subcategories. R was employed to investigate the impact of differentially expressed GlnMgs on BP, MF, and CC, and pathways.

### Drug-gene interactions

As bioinformatics advancements have led to the identification of potential biomarkers, it has become increasingly important to develop biological models and discover effective biomarkers for diagnosing diseases. However, it is crucial to understand how to effectively utilize these biomarkers in a clinical setting. Therefore, predicting drug responses based on informative markers will be vital for future prevention and treatment strategies for NSOI. Validated biomarkers serve as reference points for targeted therapies. Thus, accurate drug prediction is of utmost importance. In this study, the DGIdb (https://dgidb.genome.wustl.edu/) database was utilized to predict drug interactions with the identified hub genes.

### Identification of common miRNAs and lncRNAs

In the intricate tapestry of genetic regulation, non-coding RNA transcripts, inclusive of microRNAs (miRNAs) and long non-coding RNAs (lncRNAs), emerge as pivotal orchestrators. MiRNAs, in their capacity to modulate gene expression, wield influence through mechanisms that encompass both the augmentation and attenuation of mRNA degradation and translation. LncRNAs, distinguished as non-coding RNA entities typically spanning in the vicinity of 200 nucleotides, preside over a spectrum of physiological and biochemical cellular phenomena, mediating chromosomal modifications, transcriptional activation, and intricate networks of interference.The recent proliferation of studies in this domain has cast light upon a complex interplay between miRNAs and lncRNAs, revealing a competitive landscape where these entities vie for binding affinities with a myriad of regulatory targets. Within this competitive arena, certain competitive endogenous RNAs (ceRNAs) have surfaced, distinguished by their ability to sequester miRNAs, thus unfolding a novel dimension of lncRNA functionality.

With this backdrop, our investigation seeks to delve into the intricacies of these interactions, probing the question of whether miRNAs and lncRNAs share common regulatory motifs and partake in parallel developmental trajectories within the context of TED. To navigate this inquiry, we have enlisted the computational prowess of Perl software, aiming to illuminate the shared pathways and regulatory networks that intertwine miRNAs and lncRNAs in a dance of genetic regulation.

### Construction of a network of common mRNA-miRNA-lncRNA Genes

In the quest to unravel the intricate regulatory networks underpinning common miRNAs and lncRNAs, our study delved into the wealth of data harbored in miRTarBase and PrognoScan—two empirically validated repositories that serve as treasuries of information on miRNA-lncRNA-target gene interactions. These databases stand as pillars in the scientific community, providing a robust foundation for the exploration of molecular relationships. Employing a meticulous approach, we crafted a regulatory network by forging connections between the target genes of the miRNA-mRNA-lncRNA triad and the shared genetic components identified within the NSOI landscape. This intricate web of interactions was subsequently brought to life through visualization using Cytoscape software, a tool renowned for its ability to translate complex data into comprehensible and informative graphical representations.

## Results

### Elucidation of differentially expressed genes and dimensionality reduction via principal component analysis

**Fig. 2:**
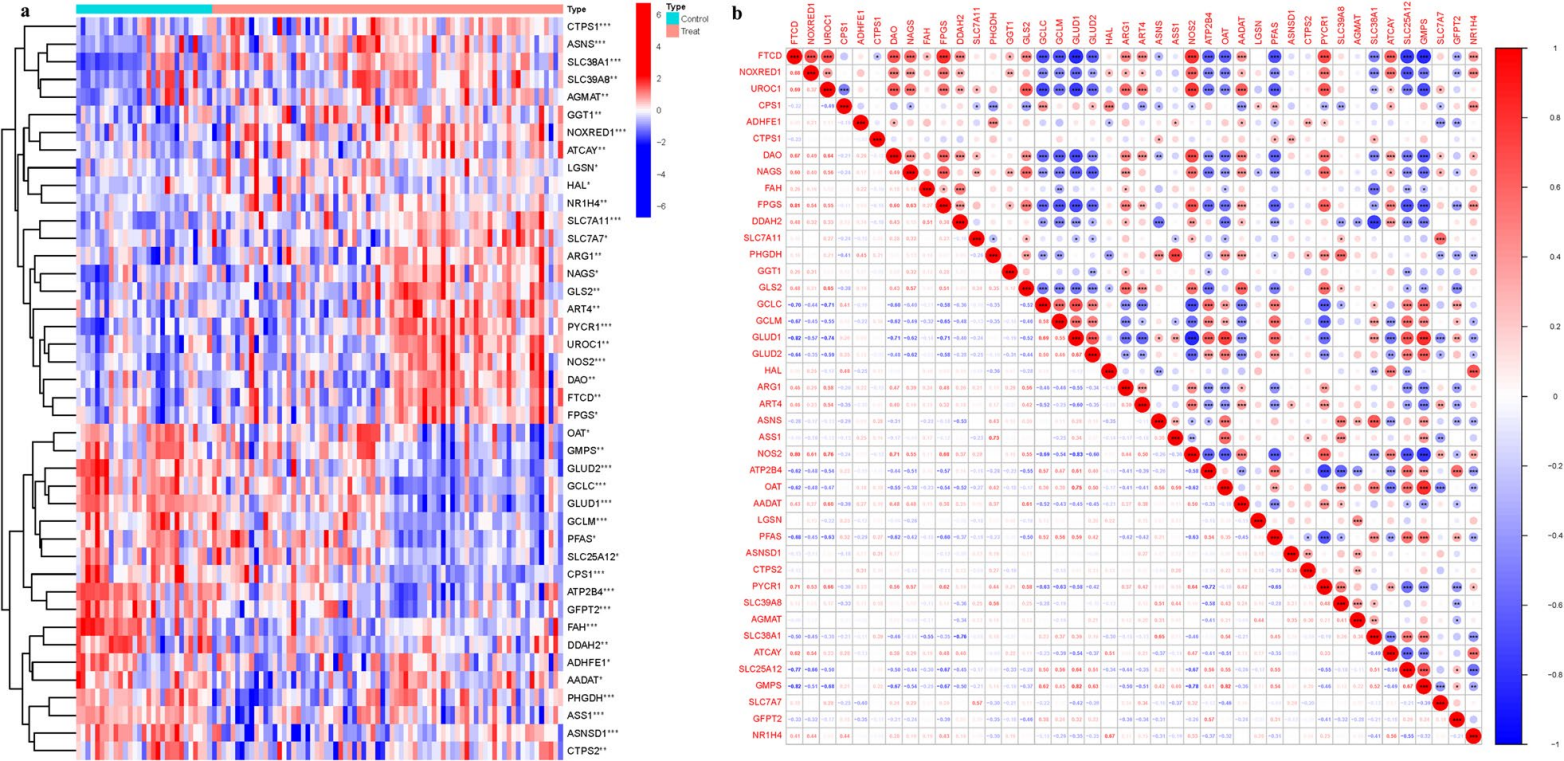
Principal Component Analysis. **a** Analysis of difference (green: low expression level; red: high expression level) of the genes participating in autophagy between the normal (N, brilliant blue) and the NSOI tissues (T, red). *P* values were showed as:*P < 0.05; **P < 0.01; ***P < 0.001). **b** Analysis of correlation

In the intricate biological tapestry of GlnMgs, a subset of 42 was meticulously analyzed. This exploration unveiled a panoply of significant fluctuations in expression profiles. A sophisticated gene clustering algorithm artfully demarcated the distinct expression topologies characteristic of the treated cohorts as opposed to the controls. Within the treatment paradigm, a suite of GlnMgs emerged as prominent, namely CTPS1, ASNS, SLC38A1, SLC39A8, AGMAT, and GGT1. Contrasting these, the control constellation comprised OAT, GMPS, GLUD2, GCLC, GLUD1, GCLM, and PFAS, among others, delineating a unique gene expression milieu (Fig. 2a). Complementing the gene expression profiling, a correlation analysis was meticulously carried out among the GlnMgs, culminating in the construction of a comprehensive correlation matrix. This matrix serves as a graphical abstract of the intricate interplay of gene expression, providing a clear visual representation of the relationships between the genes studied (Fig. 2b; Table S2).

### Functional enrichment and pathway analysis of glutamine metabolism genes

**Fig. 3:**
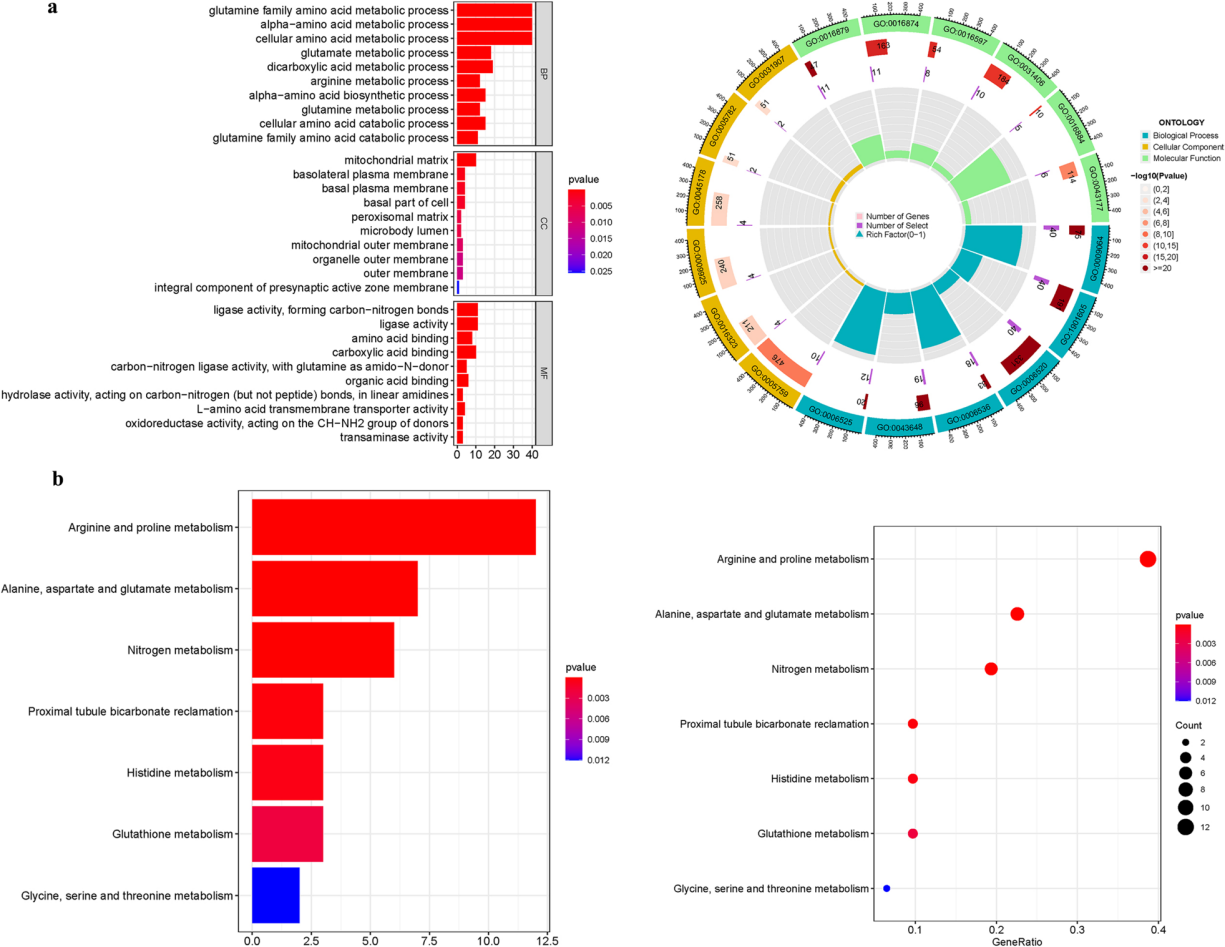
For GlnMgs, GO, and KEGG analyses were performed. **a** Bubble graph for GO enrichment (the bigger bubble means the more genes enriched, and the increasing depth of red means the differences were more obvious; q-value: the adjusted *p*-value); The GO circle shows the scatter map of the logFC of the specified gene. **b** Barplot graph for KEGG pathways (the longer bar means the more genes enriched, and the increasing depth of red means the differences were more obvious); The KEGG circle shows the scatter map of the logFC of the specified gene. The higher the Z-score value indicated, the higher expression of the enriched pathway

The comprehensive GO enrichment analysis has meticulously delineated a consortium of 515 pivotal target genes within the realm of GlnMgs, which are stratified across three fundamental categories: BP, MF, and CC. The findings in the MF domain were predominantly aligned with an array of transporter activities, including anion transmembrane transporter activity (GO:0008509), active transmembrane transporter activity (GO:0022804), and secondary active transmembrane transporter activity (GO:0015291). This indicates a significant role for GlnMgs in the facilitation of molecular trafficking across cellular membranes. In the realm of CC, the enrichment was notably concentrated within the mitochondrial matrix (GO:0005759), highlighting the centrality of mitochondria in glutamine metabolism. This was alongside significant associations with the basal part of the cell (GO:0045178) and the basal plasma membrane (GO:0009925), underscoring the structural components critical for cellular function and integrity. Within the BP category, the response to extracellular stimuli (GO:0009991), lipid transport (GO:0006869), and the metabolic processing of purine-containing compounds (GO:0072521) were particularly highlighted, reflecting the diverse and crucial roles that GlnMgs execute in the cellular response to the external environment, lipid dynamics, and nucleotide metabolism. Supplementary to the GO analysis, KEGG pathway enrichment offered a more nuanced insight, with upregulated genes predominately orchestrating the metabolic symphony of Arginine and proline (hsa00330), Alanine, aspartate, and glutamate (hsa00250), Glutathione (hsa00480), and the interlinked pathways of Glycine, serine, and threonine (hsa00260) metabolism. These findings, depicted in Fig. 3 and Tables S3a-b, underscore the multi-faceted roles of GlnMgs in maintaining cellular homeostasis and metabolic plasticity.

### Model construction synthesis of a predictive gene signature model

**Fig. 4:**
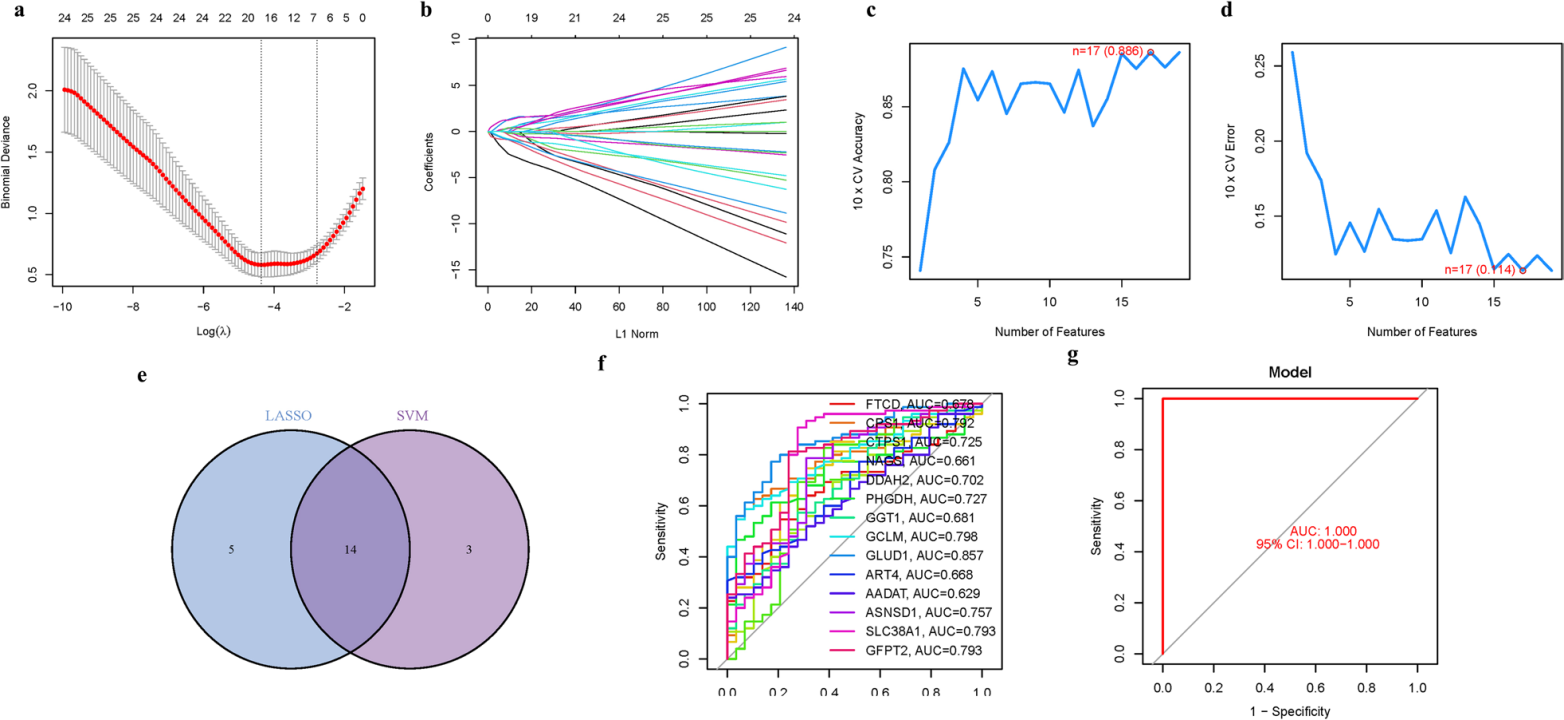
The development of the GlnMgs signature. **a** Regression of the 14 NSOI-related genes using LASSO. **b** Cross-validation is used in the LASSO regression to fine-tune parameter selection. **c**-**d** Accuracy and error of this model. **e** Venn. **f** AUC of 14 hub genes. **g** AUC of train group

Within the quantitative tapestry of gene analysis, we have engineered a prognostic gene signature utilizing the precision of LASSO logistic regression coupled with Cox proportional hazards regression analysis. This dual analytical approach has been calibrated to pinpoint an optimal penalization parameter that refines the gene selection process, an endeavor that is graphically encapsulated in Fig. 4a-b. To rigorously assess the predictive prowess and stability of our constructed model, we enlisted the aid of a machine learning algorithm-SVM-RFE. This model has exhibited commendable predictive accuracy, manifesting an impressive score of 0.886 and a concomitant error rate of a mere 0.114, as depicted in Fig. 4c-d. This robust validation underpins the potential clinical applicability of our model. The analytical rigor was further intensified by the intersection of fourteen GlnMgs discerned through both LASSO and SVM-RFE methodologies, which corroborated a remarkable consistency, detailed in Fig. 4e. In a focused examination of the model utilizing these 14 hub genes, the diagnostic veracity was quantified through ROC values, yielding high accuracy for individual genes: FTCD (AUC = 0.678), CPS1 (AUC = 0.792), CTPS1 (AUC = 0.725), NAGS (AUC = 0.661), DDAH2 (AUC = 0.702), PHGDH (AUC = 0.727), GGT1 (AUC = 0.681), GCLM (AUC = 0.798), GLUD1 (AUC = 0.857), ART4 (AUC = 0.668), AADAT (AUC = 0.629), ASNSD1 (AUC = 0.757), SLC38A1 (AUC = 0.793), GFPT2 (AUC = 0.793), which are visually represented in Fig. 4f. The clinical relevance and robustness of our model were further substantiated through its application to an independent dataset (GSE58331), in which it achieved an optimal AUC of 1.000 within a 95% confidence interval, as illustrated in Fig. 4g. This pinnacle of predictive performance indicates an exceptional level of accuracy and robustness of our model, offering a powerful tool for the prognostication based on GlnMgs signatures (Table S4).

### Delineation of key genes via gene set enrichment analysis

**Fig. 5:**
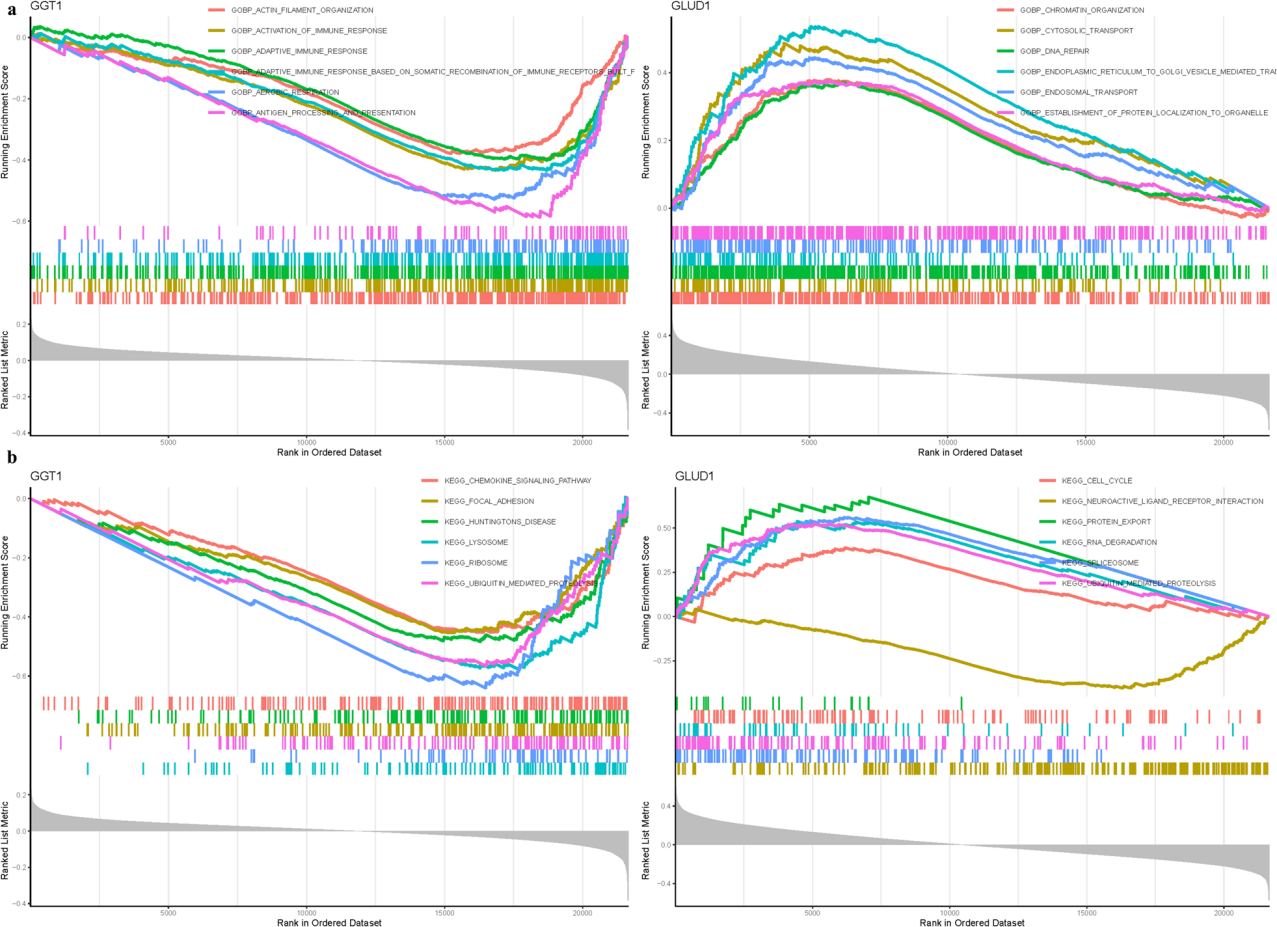
GSEA of Analysis in GGT1 and GLUD1. **a** GO. **b** KEGG

In an endeavor to pinpoint the pivotal genes instrumental to NSOI, we embarked on a meticulous review of the literature coupled with a sensitivity analysis of hub genes within our predictive model. This approach heralded the identification of GGT1 and GLUD1 as candidates of paramount relevance to the pathophysiology of NSOI. GO analysis illuminated the role of GGT1 in a trio of biological processes: it is a key player in the biosynthesis of histamine pivotal to the inflammatory response, engages in the post-translational modification of proteins through nitrosylation, and is instrumental in the immune system’s recognition of bacterial molecules. Conversely, GLUD1 emerged as a regulatory sentinel in several critical cellular processes. It governs the initiation of translation via the phosphorylation of eif2 alpha, a crucial regulatory point for protein synthesis. It also modulates the stimulatory signaling pathways mediated by Fc receptors, and is essential for the import of nuclear localization sequence (NLS)-bearing proteins into the nucleus, as graphically represented in Fig. 5a. Parallel insights were garnered from KEGG pathway analysis. GGT1 was found to be primarily linked with pathways integral to protein export, RNA degradation, and the ubiquitin-mediated proteolytic system. GLUD1, meanwhile, was associated with the regulation of the mammalian circadian rhythm, protein export mechanisms, and components of the spliceosome. These pathways underscore the multifaceted roles of these genes in cellular function and systemic regulation (Fig. 5b; Table S5).

### Immune cell landscape analysis in NSOI

**Fig. 6:**
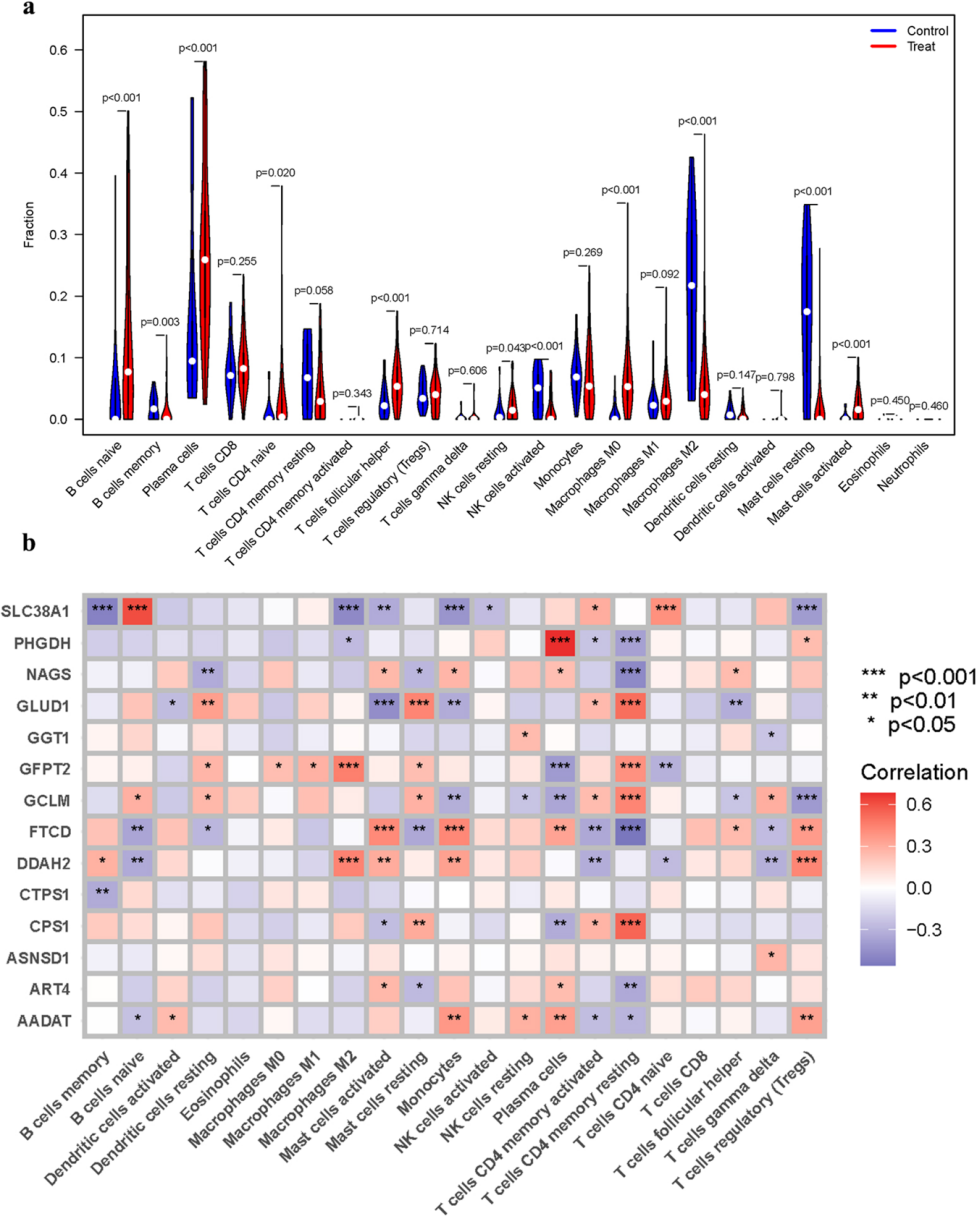
Expression of Immune cells. **a** Expression of immune cells in different clusters. **b** Correlation between GlnMgs and immune cells

The convoluted interplay within the immune microenvironment is increasingly recognized as a pivotal determinant in the pathogenesis of NSOI. To dissect this complexity, we employed violin plots to elucidate the differential expression patterns of immune cell populations between the affected and control cohorts. The results underscored a pronounced expression of B cells naive, Plasma cells, CD4 naive T cells, T follicular helper cells, Macrophages M0, and activated Mast cells within the treatment group, suggestive of an active immune response. In stark contrast, the immune milieu of the control group was characterized by an augmented presence of B cells memory, activated NK cells, M2 macrophages, and resting Mast cells. These findings, captured in Fig. 6a, reflect the dynamic nature of the immune landscape in NSOI. To further unravel the interactions between these immune cells and our previously identified gene signatures, we conducted a correlation analysis. This analysis sought to demystify the associations that may influence the behavior of immune cells within the context of NSOI. The correlation heatmap presented in Fig. 6b reveals these intricate relationships, offering a window into the potential mechanisms driving immune cell function and interaction in the disease state.

### Gene Set Variation Analysis (GSVA)

**Fig. 7:**
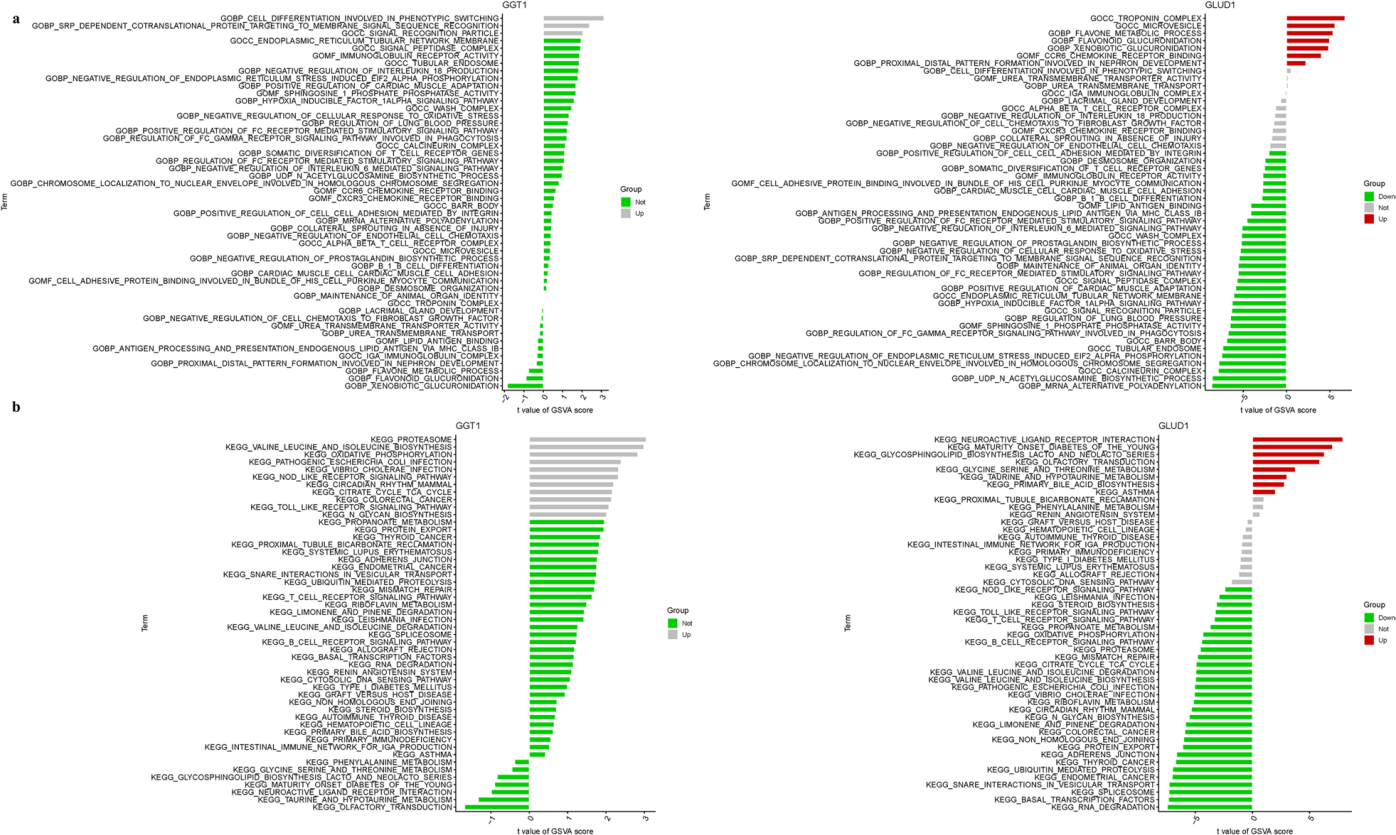
GSVA of Analysis in GGT1 and GLUD1. **a** GO. **b** KEGG

Within the framework of GO analysis, GGT1 was identified as a key regulator of several biological processes, notably cell differentiation implicated in phenotypic switching. This gene is also pivotal in the srp-dependent co-translational protein targeting to membranes, a process essential for signal sequence recognition, and plays a role in the formation of the cellular signal recognition particle component. In parallel, GLUD1 was highlighted for its integral role within the cellular troponin complex, a critical component of muscle contraction mechanisms. Additionally, it is involved in the metabolic processing of xenobiotics through glucuronidation, and the metabolism of flavonoids, a class of compounds with various biological activities. The molecular function of ccr6 chemokine receptor binding further implicates GLUD1 in the modulation of immune responses, as depicted in Fig. 7a. The insights extend into KEGG pathway analysis, where GGT1 was associated with crucial metabolic pathways such as oxidative phosphorylation and the biosynthesis of branched-chain amino acids (valine, leucine, and isoleucine). This gene also plays a role in the regulation of the proteasome, a complex responsible for protein degradation. GLUD1's involvement is marked in glycosphingolipid biosynthesis, specifically in the lacto and neolacto series, which have implications in cell–cell recognition and signaling. It also plays a role in the genetic predispositions to MODY and interacts within the intricate network of neuroactive ligand-receptor interactions, suggesting a broad spectrum of influence in metabolic and signaling pathways, as shown in Fig. 7b.

### Pharmacogenomic interactions between candidate drugs and hub genes

**Fig. 8:**
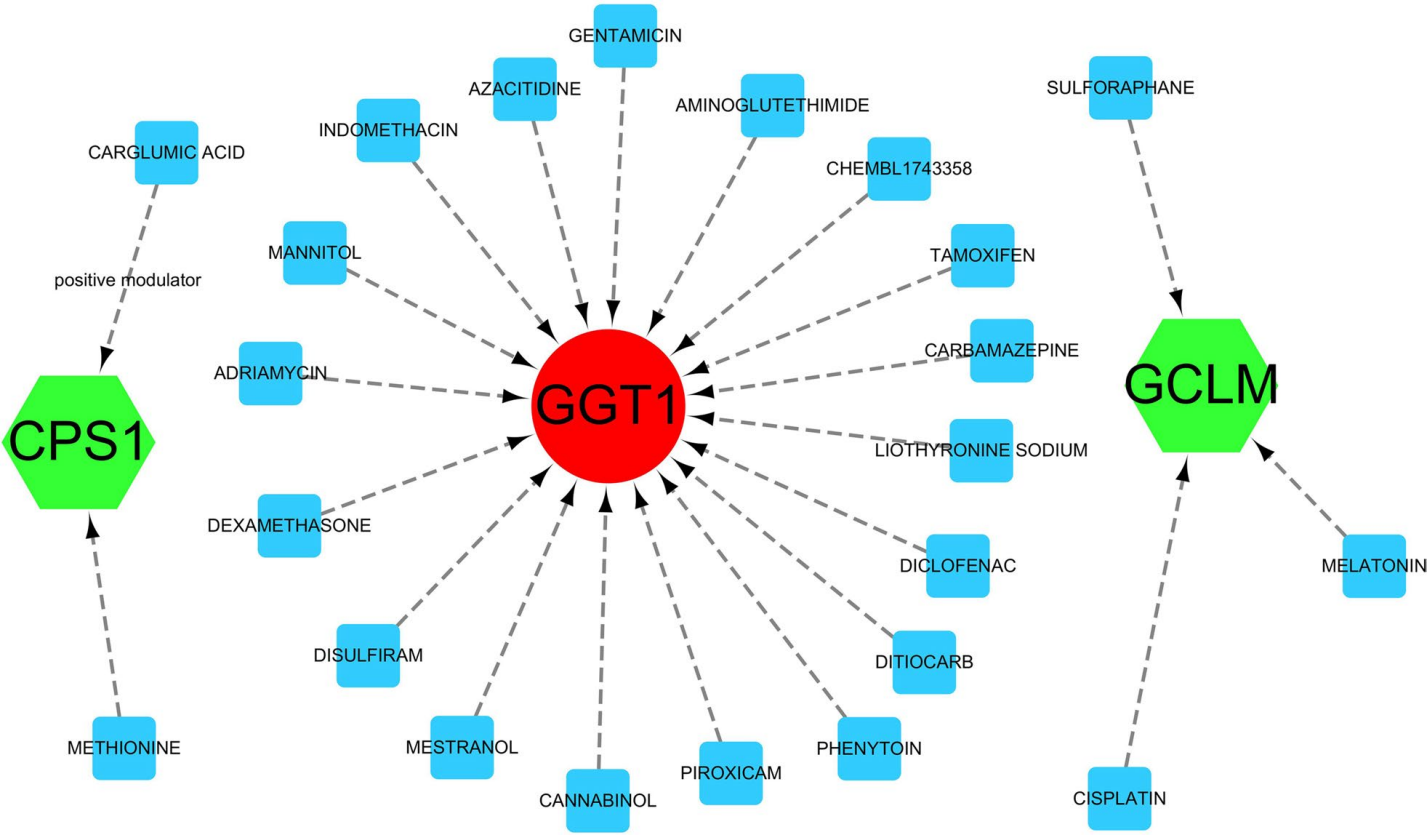
Drug-gene interactions. Note: Red circles are up-regulated genes, green hexagons are down-regulated genes, and blue squares are associated drugs

In a bid to elucidate potential therapeutic avenues, our analysis forecasted a network of interactions between a repertoire of pharmacological agents and the fourteen hub genes delineated in our study. Among these, twenty-three drugs emerged as prime candidates, with the potential to modulate the gene expression signature characteristic of NSOI. Noteworthy among these are CARGLUMIC ACID, METHIONINE, TAMOXIFEN, DITIOCARB, PIROXICAM, and DICLOFENAC, each presenting a unique profile of interaction with our genes of interest. To render these complex interactions comprehensible, we harnessed the capabilities of Cytoscape 3.7.1, a platform for visualizing molecular interaction networks. The resulting visual schema, Fig. 8, offers an intuitive representation of the drug-gene interplay, laying the groundwork for further exploration into the pharmacogenomics of NSOI and providing a springboard for future targeted therapeutic strategies, as cataloged comprehensively in Table S6.

### Elucidation of non-coding RNA networks and integration with miRNA-lncRNA-gene interactions

**Fig. 9:**
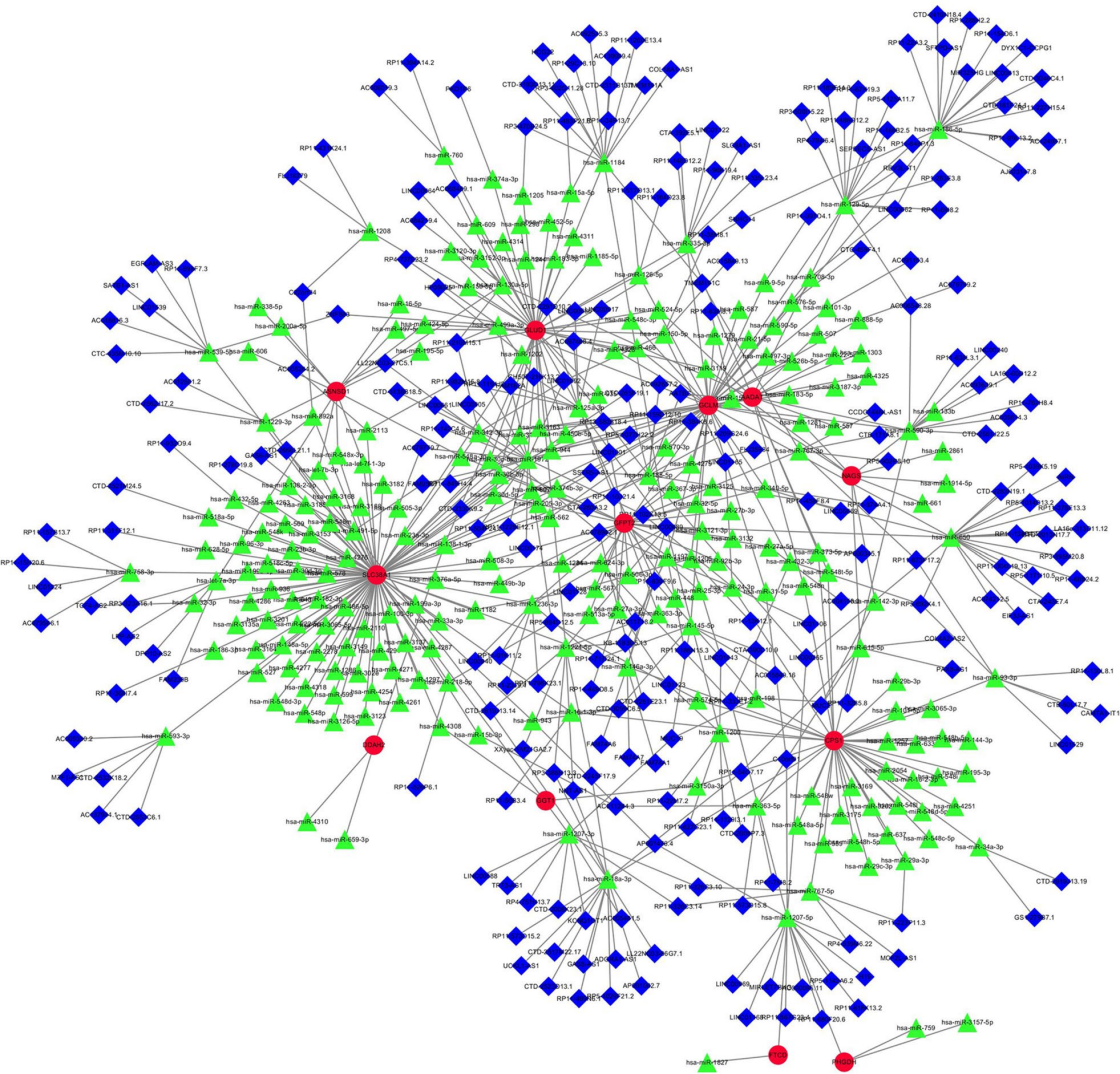
miRNAs-LncRNAs shared Genes Network. Note: Red circles are mrnas, blue quadrangles are miRNAs, and green triangles are lncRNAs

In our comprehensive analysis, we mined three extensive databases to identify non-coding RNAs implicated in the molecular tapestry of NSOI. This search yielded a substantial cohort of 293 microRNAs (miRNAs) and 334 long non-coding RNAs (lncRNAs) posited to be involved in the pathogenesis of NSOI, detailed within Table S7a-b. These databases include miRanda [19], miRDB [20], and TargetScan [21]. When the corresponding database matched the relevant miRNA, the score was marked as 1. It can be seen that when all three databases can be matched, it is 3 points. The miRNA was matched by spongeScan database [22] to obtain the corresponding lncRNA data. The miRNA-lncRNA-gene network was constructed by intersecting these non-coding RNAs with the shared genes obtained through Lasso regression and SVM-RFE. The resulting miRNA-lncRNA-gene network represents a robust framework comprising 254 lncRNAs, 235 miRNAs, and a core set of shared genes, including twelve notable hub genes: SLC38A1, GCLM, GLUD1, NAGS, AADAT, GFPT2, CPS1, ASNSD1, PHGDH, DDAH2, GGT1, and FTCD. The architectural representation of this network is showcased in Fig. 9, offering a multidimensional perspective on the regulatory axes that may underlie the complexity of NSOI. This network serves as a nexus for understanding the multifaceted interactions that span the coding and non-coding realms, shedding light on potential regulatory cascades that could be harnessed for therapeutic interventions.

### Empirical verification of central genes

**Fig. 10:**
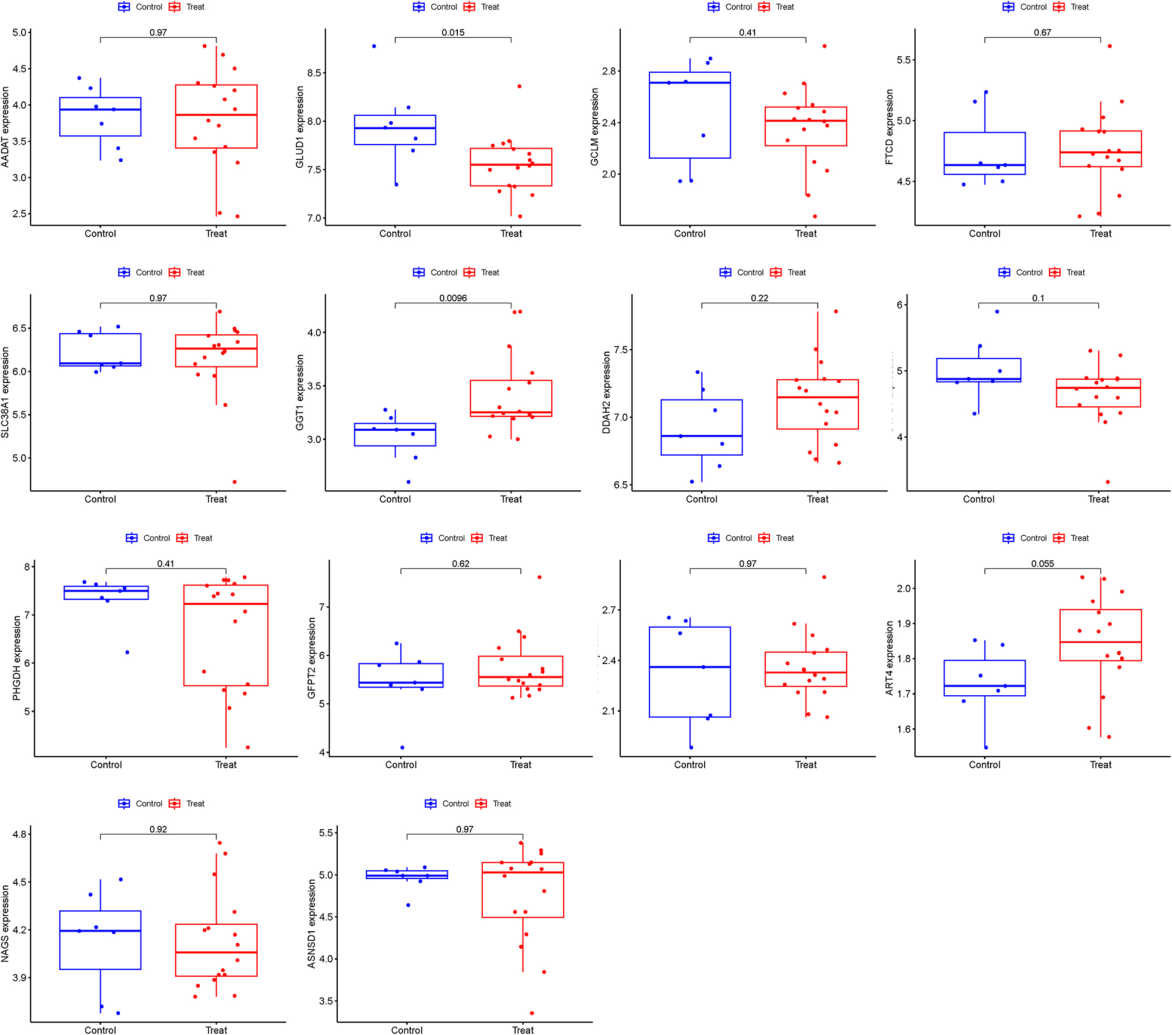
Fourteen hub genes were validated

The imperative of substantiating the predictive model's integrity led us to employ the GSE105149 dataset for a rigorous validation exercise. Within the constellation of fourteen GlnMgs that were posited as central in our model, GGT1 and GLUD1 emerged with pronounced differential expression patterns in the GSE105149 dataset analysis. This empirical evidence corroborates their proposed significance in the pathobiology of NSOI, as illustrated in Fig. 10. The consistency of these findings across independent datasets enhances the credibility of these biomarkers, underscoring their utility in deciphering the molecular underpinnings of NSOI.

## Discussions

NSOI emerges as a clinically enigmatic entity, presenting typically with unilateral, painful orbital edema devoid of identifiable viral or systemic etiologies, and harbors the potential for optic nerve compromise. The molecular intricacies that underpin this idiopathic ocular affliction remain largely enshrouded in uncertainty [23]. Nonetheless, it is becoming increasingly recognized that the regulation of gene expression may hold the key to unraveling the pathophysiological conundrums of NSOI. Gln, lauded as one of the most profuse nonessential amino acids within the circulatory milieu, assumes pivotal roles across a plethora of biosynthetic conduits in the realm of proliferating cellular populations [24]. It is esteemed for its role as a nitrogen donor in the biosynthesis of purines and pyrimidines and serves as an indispensable substrate for the synthesis of proteins and glutathione. Moreover, Gln-derived α-ketoglutarate (α-KG) is a crucial input for the TCA cycle, an essential metabolic pathway that is commandeered by cancerous cells engaging in glutaminolysis to maintain an uninterrupted supply of vital biomolecules [25]. Notably, the metabolic voracity of proliferating neoplastic cells extends beyond glucose, with Gln being requisitioned not only as an energy source but also as a fundamental scaffold for cellular architecture and function. This reliance is so pronounced that many tumor cell lineages exhibit a heightened dependence on exogenous Gln, to the extent that their very survival becomes compromised in its absence [26].

The dysregulation of Gln metabolism has been implicated in cancer development, and drugs targeting Gln metabolism have been approved for various malignancies. As cancer progresses from premalignant lesions to clinically detectable tumors and eventually to metastatic malignancies, metabolic demands and phenotypes may undergo changes. Gln metabolism has emerged as a fascinating regulatory node that exhibits variations in diverse clinical settings. Gln, the most abundant nonessential amino acid in circulation, plays a critical role in multiple cellular metabolic functions [27]. Glutaminase, an enzyme responsible for deaminating Gln to produce glutamate, serves as a key intermediate metabolite with versatile metabolic functions in the cell [28]. Recent studies have shed light on the involvement of GlnMgs in various age-related diseases. For instance, Dai et al. investigated the potential roles of Gln metabolism-related genes in hepatocellular carcinoma [29], while Liu et al. established a Gln metabolism signature for predicting prognosis in lung adenocarcinoma [30]. Although most studies have focused on the impact of individual regulators of Gln metabolism in cancer, the collective contributions of multiple Gln metabolism-related genes in other diseases remain unclear [31]. With the growing understanding of tumorigenesis, researchers have increasingly directed their attention to non-tumor aspects. Exploring distinct patterns of Gln metabolism during the progression of NSOI may provide insights into the role of Gln metabolism in NSOI pathogenesis and guide the development of appropriate therapeutic strategies.

In this investigation, we elucidated a cohort of 42 DEGs intricately connected with glutamine metabolism in NSOI. An integrative approach employing the intersection of DEGs, Lasso regression, and SVM-RFE analysis culminated in the identification of fourteen pivotal GlnMgs-namely FTCD, CPS1, CTPS1, NAGS, DDAH2, PHGDH, GGT1, GCLM, GLUD1, ART4, AADAT, ASNSD1, SLC38A1, and GFPT2. These hub genes manifested notable diagnostic potential, as substantiated by external dataset validation, intimating their probable entanglement in the molecular tapestry of NSOI pathogenesis. Whilst these insights lay the groundwork for subsequent exploration, it is imperative to acknowledge the extant paucity of evidence delineating the nexus between these genes and the orchestration of specific transcription factors within glutamine metabolism. Concurrent literature survey further discerned that GGT1 and GLUD1 may reside at the core of the association with NSOI. Further biological function analysis cast light upon their roles in myriad processes, including the response to extracellular stimuli, localization within the mitochondrial matrix, and lipid transport dynamics. This underscores the potential of GlnMgs to modulate a diverse array of biological mechanisms, possibly by steering immune-related pathways, which in turn could influence the pathophysiological trajectory of NSOI.

GGT1 overexpression has been implicated in various human diseases, including asthma, reperfusion injury, and cancer [32]. Previous studies have suggested that GGT1 and HNF1A genes may contribute to the abnormal glucose metabolism and altered lipid profile observed in Polycystic ovary syndrome, a significant clinical feature of the disorder [33]. Baumann et al. provided evidence demonstrating that recombinant and isolated hepatic human GGT1 has the ability to transform SG-3M3SH to Cys-Gly-3M3SH in vitro. This finding highlights the role of GGT1 as a key enzyme involved in the biosynthesis of Cys-Gly-3M3SH [34]. Ionotropic GluD1 and GluD2 possess the molecular architecture of postsynaptic ionotropic glutamate receptors, but they also form trans-synaptic adhesion complexes by binding to secreted cerebellins, which in turn interact with presynaptic neurexins1-4 [35]. Dai et al.'s research [36] in hippocampal synapses demonstrated that the binding of presynaptic neurexin-cerebellin complexes to postsynaptic GluD1 controls glutamate receptor activity without affecting synapse numbers. GluD proteins serve as signaling molecules that modulate NMDA and AMPA receptors through an unconventional transduction mechanism, bypassing their ionotropic receptor architecture and directly converting extracellular neurexin-cerebellin signals into postsynaptic receptor responses [37]. These findings provide further support for the validity and plausibility of our results, as GlnMgs, particularly GGT1 and GLUD1, have been implicated in the context of NSOI patients examined in this study. According to the GSE105149 research, a Gln Metabolism-related trait could serve as an effective prognostic predictor. However, only a limited number of studies have investigated the gene alterations associated with Gln Metabolism thus far.

Within the scope of NSOI, emerging perspectives contend that the augmented immune response transcends the activity of CD4 + T cells. This amplification appears to be rooted in an established milieu of T-regulatory cells and a concomitant cytokine disequilibrium, thereby precipitating a spectrum of proinflammatory and regulatory responses [38]. The perturbed reconstitution of immune competence, particularly against a backdrop of active or erstwhile opportunistic infections, is believed to exacerbate the progression of NSOI. Such infections-spanning tuberculosis, cytomegalovirus, progressive multifocal leukoencephalopathy, Kaposi sarcoma, along with a cadre of autoimmune maladies-may potentiate or covertly persist within NSOI. Notably, cytomegalovirus retinitis has garnered attention for its frequent linkage with immune reconstitution inflammatory syndrome, specifically immunological recovery uveitis [39, 40]. Mounting evidence posits that the augmentation of intracellular cAMP could serve as a salient mechanism to attenuate persistent inflammation. One tactical approach to this end involves the inhibition of its degradation, which has fostered the advancement of targeted small molecule PDE4 inhibitors [41, 42]. Such compounds have demonstrated therapeutic efficacy in a suite of inflammatory conditions, including inflammatory bowel disease, atopic dermatitis, rheumatoid arthritis, amongst others. Concomitantly, our investigation delved into the expression patterns of glutamine metabolism genes (GlnMgs) within the immune landscape, thereby extending the arc of prior research. Our observations delineated an upregulated expression of naive B cells, plasma cells, naive CD4 + T cells, follicular helper T cells, M0 macrophages, and activated mast cells in the cohort subjected to treatment. Contrastingly, the control arm manifested a predominance of memory B cells, activated NK cells, M2 macrophages, and resting mast cells. These empirical insights underscore the critical role of GlnMgs in the pathophysiological matrix of NSOI, particularly in relation to the orchestration of inflammatory and immunological responses.

The investigation of biomarkers and their association with NSOI has received limited attention in the existing literature. Currently, there are several studies utilizing bioinformatics analysis to uncover the relationship between metabolism and eye diseases [43–45]. For instance, Liu et al. identified hub genes for NSOI through Weighted Gene Coexpression Network Analysis. Hu et al. developed a bioinformatics model for thyroid eye disease and identified 11 hub genes (ATP6V1A, PTGES3, PSMD12, etc.). Huang et al. utilized advanced comprehensive bioinformatics analysis and in vivo validation to identify six genes (CD44, CDC42, TIMP1, BMP7, RHOC, FLT1) as significant genes for diabetic retinopathy. Despite the burgeoning corpus of research delineating various metabolic pathways implicated in neurological disorders, a profound lacuna persists regarding the nexus between glutamine metabolism and NSOI. Bridging this critical knowledge chasm, our investigation casts new light on the metabolic underpinnings of NSOI, proffering a promising scaffold for the development of metabolic-centric therapeutic stratagems. Employing an innovative methodological framework, our study diverges from the trodden path of prior inquiries by harnessing an expansive compendium of GlnMgs extracted from the GEO, thereby enriching the granularity and potential utility of our metabolic analysis. Despite this robust theoretical foundation and methodological ingenuity, we must acknowledge the nascent state of our findings. The cryptic intricacies governing the pathophysiological mechanisms of NSOI stubbornly elude complete scientific elucidation. Thus, it is incumbent upon the scientific community to undertake a rigorous sequence of in vivo and in vitro experimentation to substantiate the preliminary connections posited by our research. Moreover, the interplay between prognostic genes and glutamine metabolism, a domain still shrouded in mystery, beckons for a concerted investigative effort. Unraveling this relationship may unearth pivotal insights into the role of GlnMgs in NSOI pathobiology. As we stand on the precipice of these investigative forays, it is evident that the terrain ahead holds fertile ground for groundbreaking discoveries and innovative research trajectories.

To further advance our understanding and improve the management of NSOI, the following suggestions are proposed for future research: Increase the number of data sources: ① In future studies, expanding the range of data sources will contribute to a more comprehensive analysis and interpretation of NSOI-related molecular mechanisms. ② Explore the potential of effective medications: Investigate whether therapeutic interventions targeting these GlnMgs can modulate the immune microenvironment and reduce the levels of inflammatory factors associated with NSOI. This may lead to the development of novel treatment strategies for NSOI. By addressing these research directions, we can further elucidate the intricate relationship between GlnMgs and NSOI, paving the way for improved diagnostic and therapeutic approaches in the future. By addressing these research directions, we can further elucidate the intricate relationship between GlnMgs and NSOI, paving the way for improved diagnostic and therapeutic approaches in the future.

## Conclusions

The pathogenesis and progression of NSOI are the result of complex, multifactorial interactions encompassing a multitude of targets, pathways, signaling entities, and regulatory frameworks. These components engage in a synergistic and reciprocal dance that underlies the condition's intricate nature. Central to this biological interplay are the GlnMgs, which are crucial in the biosynthesis of a series of proteins including FTCD, CPS1, CTPS1, NAGS, DDAH2, PHGDH, GGT1, GCLM, GLUD1, ART4, AADAT, ASNSD1, SLC38A1, and GFPT2. Of particular note, GGT1 and GLUD1 are underscored for their prominent roles. Through their activity, they hold the capacity to exert significant influence on the metabolic circuitry, with implications that extend beyond mere biochemical pathways, potentially impacting the clinical course and therapeutic targets in NSOI.

### Supplementary Information

**Additional file 1:** **Table S1.** Glutamine Metabolism genes. **Table S2.** 42 DEGs linked to Glutamine Metabolism genes. **Table S3.** a. Analysis of GO. **Table S3.** b. Analysis of KEGG. **Table S4.** a. LASSO genes. **Table S4.** b. SVM-RFE genes. **Table S4.** c. InterGenes. **Table 5.** a. GGT1 of GSEA analysis. **Table 6.** Drug prediction. **Table 7.** a. Gene-miRNA. **Table 7.** b. Gene-lncRNA.

## Data Availability

The datasets generated during and/or analyzed during the current study are available in the appendix. Authors need to include the GEO Data set number (GSE58331 and GSE105149) here.

## Abbreviations

NSOI: Nonspecific orbital inflammation
GO: Gene Ontology
TCM: Traditional Chinese medicine
MF: Molecular functions
KEGG: Kyoto Encyclopedia of Genes and Genomes
GEO: Gene Expression Omnibus
GlnMgs: Gln-metabolism genes
BP: Biological processes
CC: Cellular components
DEGs: Differentially Expressed Genes
