# Correction: Glutamine metabolism-related genes and immunotherapy in nonspecifc orbital infammation were validated using bioinformatics and machine learning

**DOI:** 10.1186/s12864-024-10105-8

**Published:** 2024-02-29

**Authors:** Zixuan Wu, Na Li, Yuan Gao, Liyuan Cao, Xiaolei Yao, Qinghua Peng

**Affiliations:** 1 Hunan University of Traditional Chinese Medicine, 410208 Changsha, Hunan Province China; 2 Dongying People’s Hospital (Dongying Hospital of Shandong Provincial Hospital Group), 257091 Dongying, Shandong People’s Republic of China; 3 Department of Ophthalmology, the First Affiliated Hospital of Hunan University of Traditional Chinese Medicine, 410007 Changsha, Hunan Province China

**Correction: BMC Genomics 25, 71 (2024)**

10.1186/s12864-023-09946-6

Following publication of the original article it was reported that affiliations 2 and 3 were transposed, resulting in Na Li, Xiaolei Yao and Qinghua Peng having incorrect affiliations.

The correct authorship list and affiliations are given in this Correction, and the original article has been updated.

