# Supplementary material for: Glutamine metabolism-related genes and immunotherapy in nonspecific orbital inflammation were validated using bioinformatics and machine learning
Source: BMC Genomics. 2024 Jan 17;25:71. doi: 10.1186/s12864-023-09946-6 (PMC10795212; doi:10.1186/s12864-023-09946-6)
Supplement: Supplementary file 1 — Additional file 1: Table S1. Glutamine Metabolism genes. Table S2. 42 DEGs linked to Glutamine Metabolism genes. Table S3. a. Analysis of GO. Table S3. b. Analysis of KEGG. Table S4. a. LASSO genes. Table S4. b. SVM-RFE genes. Table S4. c. InterGenes. Table 5. a. GGT1 of GSEA analysis. Table 6. Drug prediction. Table 7. a. Gene-miRNA. Table 7. b. Gene-lncRNA. [file 12864_2023_9946_MOESM1_ESM.doc]

Glutamine Metabolism-Related Genes and Immunotherapy in Nonspecific Orbital Inflammation were validated using bioinformatics and machine learning

**Supplementary appendix to the manuscript**

Contents of supplementary appendix

[Appendix 1 3](#__RefHeading___Toc9423)

[Datasets and Glutamine Metabolism 3](#__RefHeading___Toc22857)

[Table S1. Glutamine Metabolism Genes 3](#__RefHeading___Toc15723)

[Appendix 2 5](#__RefHeading___Toc28929)

[DEGs linked to Glutamine Metabolism genes 5](#__RefHeading___Toc18492)

[Table S2. 42 DEGs linked to Glutamine Metabolism genes. 5](#__RefHeading___Toc30404)

[Appendix 3 7](#__RefHeading___Toc28655)

[Table S3a. Analysis of GO. 7](#__RefHeading___Toc20949)

[Table S3b. Analysis of KEGG. 48](#__RefHeading___Toc28896)

[Appendix 4 49](#__RefHeading___Toc32443)

[Table S4a. LASSO genes. 49](#__RefHeading___Toc10410)

[Table S4b. SVM-RFE genes. 50](#__RefHeading___Toc13065)

[Table S4c. InterGenes. 50](#__RefHeading___Toc13140)

[Appendix 5 50](#__RefHeading___Toc27756)

[Table 5a. GGT1 of GSEA analysis. 51](#__RefHeading___Toc2735)

[Table 5b. GLUD1 of GSEA analysis. 64](#__RefHeading___Toc11508)

[Appendix 6 74](#__RefHeading___Toc485)

[Table 6. Drug prediction. 74](#__RefHeading___Toc30466)

[Appendix 7 75](#__RefHeading___Toc1613)

[Table 7a. Gene-miRNA. 75](#__RefHeading___Toc27962)

[Table 7b. Gene-lncRNA. 86](#__RefHeading___Toc6133)

# Appendix 1

**Datasets and Glutamine Metabolism genes**

**Table S1. Glutamine Metabolism genes**

| GLYATL1B | PHGDH | ASL | PRODH2 | GAD2 |
| --- | --- | --- | --- | --- |
| MTHFS | GFPT1 | ASNS | SLC39A8 | MECP2 |
| FTCD | GGT1 | ASS1 | PYCR3 | ALDH18A1 |
| CLN3 | GLS2 | NOS1 | TAT | GAD1 |
| NOXRED1 | GCLC | NOS2 | CAD | ART4 |
| UROC1 | GCLM | NOS3 | ALDH5A1 | PYCR1 |
| CPS1 | GLS | ATP2B4 | AGMAT | NR1H4 |
| ADHFE1 | GLUD1 | OAT | DGLUCY | SLC7A11 |
| AMDHD1 | GLUD2 | OTC | ASRGL1 | MIR21 |
| CTPS1 | GLUL | AADAT | SLC38A1 | RIMKLB |
| DAO | GOT1 | LGSN | ATCAY | GFPT2 |
| NAGS | GOT2 | PFAS | SLC25A12 | DDAH1 |
| FAH | RIMKLA | ASNSD1 | ALDH4A1 | ARG2 |
| SIRT4 | PYCR2 | PPAT | GMPS | NIT2 |
| FPGS | HAL | PRODH | ARHGAP11B | GLYATL1 |
| DDAH2 | ARG1 | CTPS2 | SLC7A7 |  |

# Appendix 2

## **DEGs linked to Glutamine Metabolism genes**

**Table S2. 42 DEGs linked to Glutamine Metabolism genes.**

| Gene | conMean | treatMean | pvalue | Type |
| --- | --- | --- | --- | --- |
| FTCD | 4.760481417 | 5.06238988 | 0.005027179 | Up |
| NOXRED1 | 4.440564959 | 4.741067773 | 0.000361903 | Up |
| UROC1 | 3.309112338 | 3.615601507 | 0.007396595 | Up |
| CPS1 | 3.2279805 | 2.832557933 | 4.31E-06 | Down |
| ADHFE1 | 6.124175369 | 5.901169267 | 0.030761115 | Down |
| CTPS1 | 3.778128759 | 4.047433133 | 0.000393098 | Up |
| DAO | 3.313719959 | 3.59066068 | 0.008875361 | Up |
| NAGS | 3.570223624 | 3.85198752 | 0.011062685 | Up |
| FAH | 4.347318783 | 3.67835224 | 1.46E-05 | Down |
| FPGS | 5.687818072 | 5.92307972 | 0.016917604 | Up |
| DDAH2 | 7.579093748 | 7.18378176 | 0.001498489 | Down |
| SLC7A11 | 2.288588303 | 2.549757733 | 5.31E-06 | Up |
| PHGDH | 7.2592515 | 6.465661373 | 0.000356919 | Down |
| GGT1 | 3.022647072 | 3.311634147 | 0.004388723 | Up |
| GLS2 | 2.900068893 | 3.048904133 | 0.001209594 | Up |
| GCLC | 3.635788745 | 3.137579333 | 4.02E-06 | Down |
| GCLM | 2.460322252 | 2.09789384 | 2.64E-06 | Down |
| GLUD1 | 7.22250211 | 6.35484688 | 1.82E-08 | Down |
| GLUD2 | 4.398681659 | 3.93123584 | 1.80E-07 | Down |
| HAL | 2.005059452 | 2.10277692 | 0.013578022 | Up |
| ARG1 | 2.413358069 | 2.530939427 | 0.00574834 | Up |
| ART4 | 1.610623345 | 1.738958107 | 0.008148456 | Up |
| ASNS | 3.629403441 | 3.931939227 | 0.000289631 | Up |
| ASS1 | 8.308429248 | 7.528337333 | 0.000221324 | Down |
| NOS2 | 5.525672534 | 6.083905693 | 0.000432681 | Up |
| ATP2B4 | 7.834373945 | 6.57789792 | 5.58E-07 | Down |
| OAT | 6.848285207 | 6.216212107 | 0.017424757 | Down |
| AADAT | 3.26280079 | 3.052164707 | 0.04202274 | Down |
| LGSN | 1.333164541 | 1.468742733 | 0.035217828 | Up |
| PFAS | 4.432362052 | 4.127166293 | 0.033678548 | Down |
| ASNSD1 | 4.824395283 | 4.384968613 | 5.08E-05 | Down |
| CTPS2 | 4.273428055 | 4.045009293 | 0.007477034 | Down |
| PYCR1 | 6.004625845 | 6.70443972 | 5.08E-05 | Up |
| SLC39A8 | 4.744201572 | 5.010316853 | 0.006419441 | Up |
| AGMAT | 2.707672748 | 2.894579027 | 0.00157492 | Up |
| SLC38A1 | 4.511012248 | 5.45579092 | 3.95E-06 | Up |
| ATCAY | 4.268604962 | 4.541683293 | 0.005083883 | Up |
| SLC25A12 | 4.558812486 | 4.28713452 | 0.012390009 | Down |
| GMPS | 4.594893734 | 4.307351027 | 0.006419441 | Down |
| SLC7A7 | 4.364234955 | 4.716886413 | 0.01884351 | Up |
| GFPT2 | 6.705684466 | 5.7021586 | 4.02E-06 | Down |
| NR1H4 | 2.296218945 | 2.4634312 | 0.002416286 | Up |

# Appendix 3

**Analysis of enrichment**

**Table S3a. Analysis of GO.**

| ONTOLOGY | ID | Description | BgRatio | pvalue | qvalue | Count |
| --- | --- | --- | --- | --- | --- | --- |
| BP | GO:0009064 | glutamine family amino acid metabolic process | 75/18862 | 9.70E-101 | 6.25E-98 | 40 |
| BP | GO:1901605 | alpha-amino acid metabolic process | 191/18862 | 8.61E-81 | 2.77E-78 | 40 |
| BP | GO:0006520 | cellular amino acid metabolic process | 331/18862 | 2.12E-70 | 4.56E-68 | 40 |
| BP | GO:0006536 | glutamate metabolic process | 33/18862 | 1.46E-41 | 2.35E-39 | 18 |
| BP | GO:0043648 | dicarboxylic acid metabolic process | 96/18862 | 8.99E-34 | 1.16E-31 | 19 |
| BP | GO:0006525 | arginine metabolic process | 20/18862 | 2.33E-28 | 2.50E-26 | 12 |
| BP | GO:1901607 | alpha-amino acid biosynthetic process | 67/18862 | 1.98E-27 | 1.82E-25 | 15 |
| BP | GO:0006541 | glutamine metabolic process | 23/18862 | 2.49E-27 | 2.01E-25 | 12 |
| BP | GO:0009063 | cellular amino acid catabolic process | 106/18862 | 3.53E-24 | 2.53E-22 | 15 |
| BP | GO:0009065 | glutamine family amino acid catabolic process | 27/18862 | 1.50E-23 | 9.65E-22 | 11 |
| BP | GO:0008652 | cellular amino acid biosynthetic process | 77/18862 | 4.84E-22 | 2.84E-20 | 13 |
| BP | GO:0009084 | glutamine family amino acid biosynthetic process | 17/18862 | 1.01E-20 | 5.43E-19 | 9 |
| BP | GO:1901606 | alpha-amino acid catabolic process | 88/18862 | 3.45E-19 | 1.71E-17 | 12 |
| BP | GO:0046395 | carboxylic acid catabolic process | 243/18862 | 1.36E-18 | 6.27E-17 | 15 |
| BP | GO:0016054 | organic acid catabolic process | 258/18862 | 3.37E-18 | 1.45E-16 | 15 |
| BP | GO:0016053 | organic acid biosynthetic process | 335/18862 | 4.73E-18 | 1.90E-16 | 16 |
| BP | GO:0044282 | small molecule catabolic process | 431/18862 | 2.56E-16 | 9.71E-15 | 16 |
| BP | GO:0046394 | carboxylic acid biosynthetic process | 327/18862 | 3.88E-15 | 1.39E-13 | 14 |
| BP | GO:0006527 | arginine catabolic process | 11/18862 | 3.30E-14 | 1.12E-12 | 6 |
| BP | GO:0019627 | urea metabolic process | 13/18862 | 4.79E-11 | 1.47E-09 | 5 |
| BP | GO:0071941 | nitrogen cycle metabolic process | 13/18862 | 4.79E-11 | 1.47E-09 | 5 |
| BP | GO:0009069 | serine family amino acid metabolic process | 43/18862 | 4.14E-10 | 1.21E-08 | 6 |
| BP | GO:0044106 | cellular amine metabolic process | 158/18862 | 1.53E-09 | 4.29E-08 | 8 |
| BP | GO:0009308 | amine metabolic process | 163/18862 | 1.96E-09 | 5.27E-08 | 8 |
| BP | GO:0006560 | proline metabolic process | 10/18862 | 4.00E-09 | 1.03E-07 | 4 |
| BP | GO:0000050 | urea cycle | 11/18862 | 6.27E-09 | 1.55E-07 | 4 |
| BP | GO:0006534 | cysteine metabolic process | 12/18862 | 9.39E-09 | 2.24E-07 | 4 |
| BP | GO:0000096 | sulfur amino acid metabolic process | 37/18862 | 1.56E-08 | 3.59E-07 | 5 |
| BP | GO:0043650 | dicarboxylic acid biosynthetic process | 14/18862 | 1.89E-08 | 4.20E-07 | 4 |
| BP | GO:0006750 | glutathione biosynthetic process | 17/18862 | 4.48E-08 | 9.62E-07 | 4 |
| BP | GO:0046112 | nucleobase biosynthetic process | 18/18862 | 5.75E-08 | 1.19E-06 | 4 |
| BP | GO:0042398 | cellular modified amino acid biosynthetic process | 48/18862 | 6.03E-08 | 1.21E-06 | 5 |
| BP | GO:0019184 | nonribosomal peptide biosynthetic process | 19/18862 | 7.27E-08 | 1.41E-06 | 4 |
| BP | GO:0009066 | aspartate family amino acid metabolic process | 50/18862 | 7.43E-08 | 1.41E-06 | 5 |
| BP | GO:0015711 | organic anion transport | 376/18862 | 9.01E-08 | 1.66E-06 | 9 |
| BP | GO:0046942 | carboxylic acid transport | 284/18862 | 1.49E-07 | 2.66E-06 | 8 |
| BP | GO:0001101 | response to acid chemical | 119/18862 | 2.09E-07 | 3.56E-06 | 6 |
| BP | GO:0006575 | cellular modified amino acid metabolic process | 198/18862 | 2.10E-07 | 3.56E-06 | 7 |
| BP | GO:0006521 | regulation of cellular amino acid metabolic process | 62/18862 | 2.23E-07 | 3.68E-06 | 5 |
| BP | GO:0015807 | L-amino acid transport | 66/18862 | 3.06E-07 | 4.92E-06 | 5 |
| BP | GO:0033238 | regulation of cellular amine metabolic process | 77/18862 | 6.64E-07 | 1.02E-05 | 5 |
| BP | GO:0046209 | nitric oxide metabolic process | 77/18862 | 6.64E-07 | 1.02E-05 | 5 |
| BP | GO:2001057 | reactive nitrogen species metabolic process | 78/18862 | 7.08E-07 | 1.06E-05 | 5 |
| BP | GO:0009112 | nucleobase metabolic process | 34/18862 | 8.50E-07 | 1.24E-05 | 4 |
| BP | GO:0006865 | amino acid transport | 152/18862 | 8.84E-07 | 1.27E-05 | 6 |
| BP | GO:0006547 | histidine metabolic process | 10/18862 | 1.13E-06 | 1.59E-05 | 3 |
| BP | GO:0043200 | response to amino acid | 102/18862 | 2.69E-06 | 3.69E-05 | 5 |
| BP | GO:0006206 | pyrimidine nucleobase metabolic process | 16/18862 | 5.23E-06 | 7.03E-05 | 3 |
| BP | GO:0015849 | organic acid transport | 324/18862 | 5.63E-06 | 7.40E-05 | 7 |
| BP | GO:0043649 | dicarboxylic acid catabolic process | 17/18862 | 6.35E-06 | 8.18E-05 | 3 |
| BP | GO:1902475 | L-alpha-amino acid transmembrane transport | 63/18862 | 1.04E-05 | 0.000131739 | 4 |
| BP | GO:0042493 | response to drug | 359/18862 | 1.10E-05 | 0.000135007 | 7 |
| BP | GO:0006749 | glutathione metabolic process | 64/18862 | 1.11E-05 | 0.000135007 | 4 |
| BP | GO:0001889 | liver development | 138/18862 | 1.18E-05 | 0.000141012 | 5 |
| BP | GO:0061008 | hepaticobiliary system development | 140/18862 | 1.27E-05 | 0.000148459 | 5 |
| BP | GO:0006790 | sulfur compound metabolic process | 378/18862 | 1.53E-05 | 0.000176526 | 7 |
| BP | GO:0042180 | cellular ketone metabolic process | 254/18862 | 1.70E-05 | 0.000192421 | 6 |
| BP | GO:0006809 | nitric oxide biosynthetic process | 73/18862 | 1.88E-05 | 0.000208373 | 4 |
| BP | GO:0009165 | nucleotide biosynthetic process | 264/18862 | 2.12E-05 | 0.000231178 | 6 |
| BP | GO:1901293 | nucleoside phosphate biosynthetic process | 267/18862 | 2.26E-05 | 0.000242266 | 6 |
| BP | GO:0071377 | cellular response to glucagon stimulus | 26/18862 | 2.39E-05 | 0.000252807 | 3 |
| BP | GO:0007263 | nitric oxide mediated signal transduction | 27/18862 | 2.69E-05 | 0.000279398 | 3 |
| BP | GO:0072593 | reactive oxygen species metabolic process | 281/18862 | 3.01E-05 | 0.000307465 | 6 |
| BP | GO:0062012 | regulation of small molecule metabolic process | 437/18862 | 3.89E-05 | 0.000391064 | 7 |
| BP | GO:0015718 | monocarboxylic acid transport | 178/18862 | 4.03E-05 | 0.000399025 | 5 |
| BP | GO:0010565 | regulation of cellular ketone metabolic process | 185/18862 | 4.84E-05 | 0.000472311 | 5 |
| BP | GO:0009260 | ribonucleotide biosynthetic process | 188/18862 | 5.22E-05 | 0.000502267 | 5 |
| BP | GO:0003333 | amino acid transmembrane transport | 96/18862 | 5.52E-05 | 0.00052323 | 4 |
| BP | GO:0046390 | ribose phosphate biosynthetic process | 195/18862 | 6.21E-05 | 0.00058016 | 5 |
| BP | GO:0032496 | response to lipopolysaccharide | 326/18862 | 6.87E-05 | 0.000632593 | 6 |
| BP | GO:0033762 | response to glucagon | 37/18862 | 7.04E-05 | 0.000638403 | 3 |
| BP | GO:0071398 | cellular response to fatty acid | 38/18862 | 7.63E-05 | 0.000673117 | 3 |
| BP | GO:0071548 | response to dexamethasone | 38/18862 | 7.63E-05 | 0.000673117 | 3 |
| BP | GO:0072528 | pyrimidine-containing compound biosynthetic process | 40/18862 | 8.91E-05 | 0.000775338 | 3 |
| BP | GO:0002237 | response to molecule of bacterial origin | 346/18862 | 9.54E-05 | 0.000819598 | 6 |
| BP | GO:0014075 | response to amine | 42/18862 | 0.000103174 | 0.000874548 | 3 |
| BP | GO:1903409 | reactive oxygen species biosynthetic process | 123/18862 | 0.000144685 | 0.001210491 | 4 |
| BP | GO:0009636 | response to toxic substance | 239/18862 | 0.000161784 | 0.001336189 | 5 |
| BP | GO:0003018 | vascular process in circulatory system | 245/18862 | 0.000181585 | 0.001480746 | 5 |
| BP | GO:0051712 | positive regulation of killing of cells of other organism | 10/18862 | 0.00020517 | 0.001652156 | 2 |
| BP | GO:0010043 | response to zinc ion | 57/18862 | 0.000257085 | 0.002044653 | 3 |
| BP | GO:0045428 | regulation of nitric oxide biosynthetic process | 59/18862 | 0.000284772 | 0.002237231 | 3 |
| BP | GO:0009259 | ribonucleotide metabolic process | 425/18862 | 0.000291206 | 0.002260214 | 6 |
| BP | GO:0070542 | response to fatty acid | 61/18862 | 0.000314316 | 0.002382181 | 3 |
| BP | GO:0080164 | regulation of nitric oxide metabolic process | 61/18862 | 0.000314316 | 0.002382181 | 3 |
| BP | GO:0019693 | ribose phosphate metabolic process | 435/18862 | 0.000329771 | 0.002470256 | 6 |
| BP | GO:1905039 | carboxylic acid transmembrane transport | 154/18862 | 0.000342093 | 0.0025331 | 4 |
| BP | GO:1903825 | organic acid transmembrane transport | 155/18862 | 0.000350606 | 0.002535043 | 4 |
| BP | GO:0007494 | midgut development | 13/18862 | 0.00035416 | 0.002535043 | 2 |
| BP | GO:0032354 | response to follicle-stimulating hormone | 13/18862 | 0.00035416 | 0.002535043 | 2 |
| BP | GO:0031667 | response to nutrient levels | 451/18862 | 0.000399724 | 0.002829738 | 6 |
| BP | GO:0006241 | CTP biosynthetic process | 14/18862 | 0.000412618 | 0.002841439 | 2 |
| BP | GO:0072350 | tricarboxylic acid metabolic process | 14/18862 | 0.000412618 | 0.002841439 | 2 |
| BP | GO:0015800 | acidic amino acid transport | 67/18862 | 0.000414609 | 0.002841439 | 3 |
| BP | GO:0007584 | response to nutrient | 165/18862 | 0.000444288 | 0.003012792 | 4 |
| BP | GO:0009209 | pyrimidine ribonucleoside triphosphate biosynthetic process | 15/18862 | 0.000475443 | 0.003190472 | 2 |
| BP | GO:0009201 | ribonucleoside triphosphate biosynthetic process | 73/18862 | 0.000533451 | 0.003393773 | 3 |
| BP | GO:0009991 | response to extracellular stimulus | 477/18862 | 0.000537706 | 0.003393773 | 6 |
| BP | GO:0006103 | 2-oxoglutarate metabolic process | 16/18862 | 0.000542615 | 0.003393773 | 2 |
| BP | GO:0017014 | protein nitrosylation | 16/18862 | 0.000542615 | 0.003393773 | 2 |
| BP | GO:0018119 | peptidyl-cysteine S-nitrosylation | 16/18862 | 0.000542615 | 0.003393773 | 2 |
| BP | GO:0046036 | CTP metabolic process | 16/18862 | 0.000542615 | 0.003393773 | 2 |
| BP | GO:0051709 | regulation of killing of cells of other organism | 16/18862 | 0.000542615 | 0.003393773 | 2 |
| BP | GO:0009208 | pyrimidine ribonucleoside triphosphate metabolic process | 18/18862 | 0.000689932 | 0.004232968 | 2 |
| BP | GO:0015802 | basic amino acid transport | 18/18862 | 0.000689932 | 0.004232968 | 2 |
| BP | GO:0009148 | pyrimidine nucleoside triphosphate biosynthetic process | 19/18862 | 0.00077004 | 0.004679886 | 2 |
| BP | GO:0044272 | sulfur compound biosynthetic process | 193/18862 | 0.000800311 | 0.004794732 | 4 |
| BP | GO:0009142 | nucleoside triphosphate biosynthetic process | 84/18862 | 0.000803823 | 0.004794732 | 3 |
| BP | GO:0072527 | pyrimidine-containing compound metabolic process | 85/18862 | 0.000831978 | 0.004917146 | 3 |
| BP | GO:0009074 | aromatic amino acid family catabolic process | 20/18862 | 0.000854423 | 0.004968964 | 2 |
| BP | GO:0006164 | purine nucleotide biosynthetic process | 197/18862 | 0.000863885 | 0.004968964 | 4 |
| BP | GO:0071222 | cellular response to lipopolysaccharide | 197/18862 | 0.000863885 | 0.004968964 | 4 |
| BP | GO:0009199 | ribonucleoside triphosphate metabolic process | 87/18862 | 0.000890154 | 0.005074747 | 3 |
| BP | GO:0009067 | aspartate family amino acid biosynthetic process | 21/18862 | 0.000943064 | 0.005237341 | 2 |
| BP | GO:0009070 | serine family amino acid biosynthetic process | 21/18862 | 0.000943064 | 0.005237341 | 2 |
| BP | GO:0009168 | purine ribonucleoside monophosphate biosynthetic process | 21/18862 | 0.000943064 | 0.005237341 | 2 |
| BP | GO:0009220 | pyrimidine ribonucleotide biosynthetic process | 22/18862 | 0.001035944 | 0.005608116 | 2 |
| BP | GO:0030810 | positive regulation of nucleotide biosynthetic process | 22/18862 | 0.001035944 | 0.005608116 | 2 |
| BP | GO:1900373 | positive regulation of purine nucleotide biosynthetic process | 22/18862 | 0.001035944 | 0.005608116 | 2 |
| BP | GO:0072522 | purine-containing compound biosynthetic process | 208/18862 | 0.001057023 | 0.005674545 | 4 |
| BP | GO:0071219 | cellular response to molecule of bacterial origin | 209/18862 | 0.001075965 | 0.005702804 | 4 |
| BP | GO:0006835 | dicarboxylic acid transport | 93/18862 | 0.001079992 | 0.005702804 | 3 |
| BP | GO:0009127 | purine nucleoside monophosphate biosynthetic process | 23/18862 | 0.001133045 | 0.005934305 | 2 |
| BP | GO:0034698 | response to gonadotropin | 24/18862 | 0.00123435 | 0.006412751 | 2 |
| BP | GO:1903426 | regulation of reactive oxygen species biosynthetic process | 99/18862 | 0.001293667 | 0.006667154 | 3 |
| BP | GO:0009147 | pyrimidine nucleoside triphosphate metabolic process | 25/18862 | 0.00133984 | 0.006743276 | 2 |
| BP | GO:0015813 | L-glutamate transmembrane transport | 25/18862 | 0.00133984 | 0.006743276 | 2 |
| BP | GO:0042537 | benzene-containing compound metabolic process | 25/18862 | 0.00133984 | 0.006743276 | 2 |
| BP | GO:0062014 | negative regulation of small molecule metabolic process | 101/18862 | 0.001370378 | 0.006843503 | 3 |
| BP | GO:0006576 | cellular biogenic amine metabolic process | 103/18862 | 0.001449896 | 0.007184909 | 3 |
| BP | GO:0098657 | import into cell | 230/18862 | 0.001531177 | 0.007513501 | 4 |
| BP | GO:0006760 | folic acid-containing compound metabolic process | 27/18862 | 0.001563308 | 0.007513501 | 2 |
| BP | GO:0015949 | nucleobase-containing small molecule interconversion | 27/18862 | 0.001563308 | 0.007513501 | 2 |
| BP | GO:0018195 | peptidyl-arginine modification | 27/18862 | 0.001563308 | 0.007513501 | 2 |
| BP | GO:0018958 | phenol-containing compound metabolic process | 106/18862 | 0.00157452 | 0.007513501 | 3 |
| BP | GO:0071216 | cellular response to biotic stimulus | 233/18862 | 0.001605648 | 0.007605699 | 4 |
| BP | GO:0009141 | nucleoside triphosphate metabolic process | 109/18862 | 0.001705674 | 0.008020532 | 3 |
| BP | GO:0009218 | pyrimidine ribonucleotide metabolic process | 29/18862 | 0.001803305 | 0.008357612 | 2 |
| BP | GO:0071549 | cellular response to dexamethasone stimulus | 29/18862 | 0.001803305 | 0.008357612 | 2 |
| BP | GO:0048732 | gland development | 413/18862 | 0.001924227 | 0.008815444 | 5 |
| BP | GO:0006221 | pyrimidine nucleotide biosynthetic process | 30/18862 | 0.001929459 | 0.008815444 | 2 |
| BP | GO:0009072 | aromatic amino acid family metabolic process | 32/18862 | 0.002193988 | 0.009883847 | 2 |
| BP | GO:0009310 | amine catabolic process | 32/18862 | 0.002193988 | 0.009883847 | 2 |
| BP | GO:0042558 | pteridine-containing compound metabolic process | 33/18862 | 0.002332329 | 0.010434102 | 2 |
| BP | GO:0009156 | ribonucleoside monophosphate biosynthetic process | 34/18862 | 0.002474697 | 0.010919356 | 2 |
| BP | GO:0055081 | anion homeostasis | 34/18862 | 0.002474697 | 0.010919356 | 2 |
| BP | GO:0006163 | purine nucleotide metabolic process | 441/18862 | 0.00255888 | 0.011213996 | 5 |
| BP | GO:0006979 | response to oxidative stress | 444/18862 | 0.00263493 | 0.01132253 | 5 |
| BP | GO:0035296 | regulation of tube diameter | 127/18862 | 0.002636373 | 0.01132253 | 3 |
| BP | GO:0097746 | blood vessel diameter maintenance | 127/18862 | 0.002636373 | 0.01132253 | 3 |
| BP | GO:0035150 | regulation of tube size | 128/18862 | 0.002695598 | 0.011500215 | 3 |
| BP | GO:0072521 | purine-containing compound metabolic process | 460/18862 | 0.003068654 | 0.012873312 | 5 |
| BP | GO:0042401 | cellular biogenic amine biosynthetic process | 38/18862 | 0.003084103 | 0.012873312 | 2 |
| BP | GO:0045429 | positive regulation of nitric oxide biosynthetic process | 38/18862 | 0.003084103 | 0.012873312 | 2 |
| BP | GO:0006869 | lipid transport | 461/18862 | 0.003097378 | 0.012873312 | 5 |
| BP | GO:0051384 | response to glucocorticoid | 135/18862 | 0.003133284 | 0.012939069 | 3 |
| BP | GO:0009309 | amine biosynthetic process | 39/18862 | 0.003246351 | 0.013153041 | 2 |
| BP | GO:0060416 | response to growth hormone | 39/18862 | 0.003246351 | 0.013153041 | 2 |
| BP | GO:1904407 | positive regulation of nitric oxide metabolic process | 39/18862 | 0.003246351 | 0.013153041 | 2 |
| BP | GO:0006730 | one-carbon metabolic process | 40/18862 | 0.003412524 | 0.013654558 | 2 |
| BP | GO:0009167 | purine ribonucleoside monophosphate metabolic process | 40/18862 | 0.003412524 | 0.013654558 | 2 |
| BP | GO:0062013 | positive regulation of small molecule metabolic process | 141/18862 | 0.003541307 | 0.014082391 | 3 |
| BP | GO:1900371 | regulation of purine nucleotide biosynthetic process | 42/18862 | 0.003756576 | 0.014846785 | 2 |
| BP | GO:0009124 | nucleoside monophosphate biosynthetic process | 43/18862 | 0.003934422 | 0.015086881 | 2 |
| BP | GO:0009126 | purine nucleoside monophosphate metabolic process | 43/18862 | 0.003934422 | 0.015086881 | 2 |
| BP | GO:0030808 | regulation of nucleotide biosynthetic process | 43/18862 | 0.003934422 | 0.015086881 | 2 |
| BP | GO:0045454 | cell redox homeostasis | 43/18862 | 0.003934422 | 0.015086881 | 2 |
| BP | GO:0046189 | phenol-containing compound biosynthetic process | 43/18862 | 0.003934422 | 0.015086881 | 2 |
| BP | GO:0015908 | fatty acid transport | 150/18862 | 0.004211937 | 0.016055467 | 3 |
| BP | GO:0031960 | response to corticosteroid | 152/18862 | 0.004370719 | 0.016562724 | 3 |
| BP | GO:0045981 | positive regulation of nucleotide metabolic process | 46/18862 | 0.004491034 | 0.016820762 | 2 |
| BP | GO:1900544 | positive regulation of purine nucleotide metabolic process | 46/18862 | 0.004491034 | 0.016820762 | 2 |
| BP | GO:0018198 | peptidyl-cysteine modification | 48/18862 | 0.004881171 | 0.018176309 | 2 |
| BP | GO:0098739 | import across plasma membrane | 161/18862 | 0.005130141 | 0.018993627 | 3 |
| BP | GO:0006220 | pyrimidine nucleotide metabolic process | 50/18862 | 0.005286403 | 0.019197601 | 2 |
| BP | GO:1902001 | fatty acid transmembrane transport | 50/18862 | 0.005286403 | 0.019197601 | 2 |
| BP | GO:1903202 | negative regulation of oxidative stress-induced cell death | 50/18862 | 0.005286403 | 0.019197601 | 2 |
| BP | GO:0071375 | cellular response to peptide hormone stimulus | 325/18862 | 0.005304435 | 0.019197601 | 4 |
| BP | GO:0050999 | regulation of nitric-oxide synthase activity | 52/18862 | 0.005706598 | 0.020537711 | 2 |
| BP | GO:0071320 | cellular response to cAMP | 53/18862 | 0.005922265 | 0.021195476 | 2 |
| BP | GO:0001912 | positive regulation of leukocyte mediated cytotoxicity | 55/18862 | 0.00636466 | 0.022405359 | 2 |
| BP | GO:0071385 | cellular response to glucocorticoid stimulus | 55/18862 | 0.00636466 | 0.022405359 | 2 |
| BP | GO:1903428 | positive regulation of reactive oxygen species biosynthetic process | 55/18862 | 0.00636466 | 0.022405359 | 2 |
| BP | GO:0009152 | purine ribonucleotide biosynthetic process | 175/18862 | 0.006461294 | 0.02262192 | 3 |
| BP | GO:0009161 | ribonucleoside monophosphate metabolic process | 56/18862 | 0.006591353 | 0.022952537 | 2 |
| BP | GO:0071346 | cellular response to interferon-gamma | 177/18862 | 0.006666697 | 0.023090088 | 3 |
| BP | GO:0050796 | regulation of insulin secretion | 178/18862 | 0.006770848 | 0.02332541 | 3 |
| BP | GO:0046148 | pigment biosynthetic process | 57/18862 | 0.006821691 | 0.023375558 | 2 |
| BP | GO:0010038 | response to metal ion | 352/18862 | 0.007008785 | 0.023889593 | 4 |
| BP | GO:0071384 | cellular response to corticosteroid stimulus | 60/18862 | 0.007534399 | 0.025545996 | 2 |
| BP | GO:0071230 | cellular response to amino acid stimulus | 61/18862 | 0.007779148 | 0.026237742 | 2 |
| BP | GO:0060541 | respiratory system development | 188/18862 | 0.007866037 | 0.026392626 | 3 |
| BP | GO:0031343 | positive regulation of cell killing | 63/18862 | 0.008279318 | 0.027635356 | 2 |
| BP | GO:2000377 | regulation of reactive oxygen species metabolic process | 192/18862 | 0.008331739 | 0.027666979 | 3 |
| BP | GO:1901654 | response to ketone | 193/18862 | 0.008450656 | 0.027917957 | 3 |
| BP | GO:0032768 | regulation of monooxygenase activity | 65/18862 | 0.008793613 | 0.028902747 | 2 |
| BP | GO:0034341 | response to interferon-gamma | 197/18862 | 0.00893635 | 0.029222796 | 3 |
| BP | GO:0031640 | killing of cells of other organism | 67/18862 | 0.009321908 | 0.030329654 | 2 |
| BP | GO:0006625 | protein targeting to peroxisome | 68/18862 | 0.009591266 | 0.030588093 | 2 |
| BP | GO:0072662 | protein localization to peroxisome | 68/18862 | 0.009591266 | 0.030588093 | 2 |
| BP | GO:0072663 | establishment of protein localization to peroxisome | 68/18862 | 0.009591266 | 0.030588093 | 2 |
| BP | GO:1903201 | regulation of oxidative stress-induced cell death | 68/18862 | 0.009591266 | 0.030588093 | 2 |
| BP | GO:0071229 | cellular response to acid chemical | 69/18862 | 0.009864078 | 0.031303165 | 2 |
| BP | GO:1901653 | cellular response to peptide | 391/18862 | 0.010059738 | 0.031767592 | 4 |
| BP | GO:0030073 | insulin secretion | 207/18862 | 0.010221384 | 0.032120601 | 3 |
| BP | GO:0032024 | positive regulation of insulin secretion | 71/18862 | 0.010419997 | 0.032428365 | 2 |
| BP | GO:0051881 | regulation of mitochondrial membrane potential | 71/18862 | 0.010419997 | 0.032428365 | 2 |
| BP | GO:0090276 | regulation of peptide hormone secretion | 209/18862 | 0.010490636 | 0.03249124 | 3 |
| BP | GO:0042440 | pigment metabolic process | 72/18862 | 0.010703074 | 0.032833489 | 2 |
| BP | GO:0043574 | peroxisomal transport | 72/18862 | 0.010703074 | 0.032833489 | 2 |
| BP | GO:0006487 | protein N-linked glycosylation | 74/18862 | 0.011279386 | 0.034437437 | 2 |
| BP | GO:0009123 | nucleoside monophosphate metabolic process | 75/18862 | 0.011572591 | 0.03515439 | 2 |
| BP | GO:0009150 | purine ribonucleotide metabolic process | 408/18862 | 0.011623351 | 0.03515439 | 4 |
| BP | GO:0001960 | negative regulation of cytokine-mediated signaling pathway | 76/18862 | 0.011869141 | 0.035730025 | 2 |
| BP | GO:0001910 | regulation of leukocyte mediated cytotoxicity | 79/18862 | 0.012778711 | 0.038111945 | 2 |
| BP | GO:1990830 | cellular response to leukemia inhibitory factor | 79/18862 | 0.012778711 | 0.038111945 | 2 |
| BP | GO:1990823 | response to leukemia inhibitory factor | 80/18862 | 0.013088491 | 0.038855961 | 2 |
| BP | GO:0060761 | negative regulation of response to cytokine stimulus | 81/18862 | 0.013401541 | 0.03942198 | 2 |
| BP | GO:0140353 | lipid export from cell | 81/18862 | 0.013401541 | 0.03942198 | 2 |
| BP | GO:0007031 | peroxisome organization | 84/18862 | 0.01436016 | 0.041833246 | 2 |
| BP | GO:1900407 | regulation of cellular response to oxidative stress | 84/18862 | 0.01436016 | 0.041833246 | 2 |
| BP | GO:0043434 | response to peptide hormone | 435/18862 | 0.014416065 | 0.041833246 | 4 |
| BP | GO:0150104 | transport across blood-brain barrier | 87/18862 | 0.015347673 | 0.044336917 | 2 |
| BP | GO:0010232 | vascular transport | 88/18862 | 0.015683196 | 0.045103928 | 2 |
| BP | GO:0036473 | cell death in response to oxidative stress | 89/18862 | 0.016021871 | 0.045873145 | 2 |
| BP | GO:0070301 | cellular response to hydrogen peroxide | 90/18862 | 0.016363682 | 0.046539786 | 2 |
| BP | GO:0030072 | peptide hormone secretion | 247/18862 | 0.016399191 | 0.046539786 | 3 |
| BP | GO:1901655 | cellular response to ketone | 92/18862 | 0.017056658 | 0.048133162 | 2 |
| BP | GO:1901617 | organic hydroxy compound biosynthetic process | 251/18862 | 0.017110081 | 0.048133162 | 3 |
| BP | GO:0051591 | response to cAMP | 93/18862 | 0.017407794 | 0.048546684 | 2 |
| BP | GO:1902882 | regulation of response to oxidative stress | 93/18862 | 0.017407794 | 0.048546684 | 2 |
| BP | GO:0090277 | positive regulation of peptide hormone secretion | 95/18862 | 0.018119288 | 0.050313086 | 2 |
| BP | GO:0046883 | regulation of hormone secretion | 260/18862 | 0.018772306 | 0.051654675 | 3 |
| BP | GO:0098659 | inorganic cation import across plasma membrane | 97/18862 | 0.018842984 | 0.051654675 | 2 |
| BP | GO:0099587 | inorganic ion import across plasma membrane | 97/18862 | 0.018842984 | 0.051654675 | 2 |
| BP | GO:0031341 | regulation of cell killing | 98/18862 | 0.019209372 | 0.052435931 | 2 |
| BP | GO:2000379 | positive regulation of reactive oxygen species metabolic process | 101/18862 | 0.020326527 | 0.054607037 | 2 |
| BP | GO:0002725 | negative regulation of T cell cytokine production | 10/18862 | 0.02153052 | 0.054607037 | 1 |
| BP | GO:0006188 | IMP biosynthetic process | 10/18862 | 0.02153052 | 0.054607037 | 1 |
| BP | GO:0006569 | tryptophan catabolic process | 10/18862 | 0.02153052 | 0.054607037 | 1 |
| BP | GO:0009113 | purine nucleobase biosynthetic process | 10/18862 | 0.02153052 | 0.054607037 | 1 |
| BP | GO:0009396 | folic acid-containing compound biosynthetic process | 10/18862 | 0.02153052 | 0.054607037 | 1 |
| BP | GO:0031284 | positive regulation of guanylate cyclase activity | 10/18862 | 0.02153052 | 0.054607037 | 1 |
| BP | GO:0035999 | tetrahydrofolate interconversion | 10/18862 | 0.02153052 | 0.054607037 | 1 |
| BP | GO:0042436 | indole-containing compound catabolic process | 10/18862 | 0.02153052 | 0.054607037 | 1 |
| BP | GO:0046218 | indolalkylamine catabolic process | 10/18862 | 0.02153052 | 0.054607037 | 1 |
| BP | GO:0051351 | positive regulation of ligase activity | 10/18862 | 0.02153052 | 0.054607037 | 1 |
| BP | GO:0070942 | neutrophil mediated cytotoxicity | 10/18862 | 0.02153052 | 0.054607037 | 1 |
| BP | GO:0071073 | positive regulation of phospholipid biosynthetic process | 10/18862 | 0.02153052 | 0.054607037 | 1 |
| BP | GO:0072537 | fibroblast activation | 10/18862 | 0.02153052 | 0.054607037 | 1 |
| BP | GO:0106072 | negative regulation of adenylate cyclase-activating G protein-coupled receptor signaling pathway | 10/18862 | 0.02153052 | 0.054607037 | 1 |
| BP | GO:1901748 | leukotriene D4 metabolic process | 10/18862 | 0.02153052 | 0.054607037 | 1 |
| BP | GO:1901750 | leukotriene D4 biosynthetic process | 10/18862 | 0.02153052 | 0.054607037 | 1 |
| BP | GO:2000169 | regulation of peptidyl-cysteine S-nitrosylation | 10/18862 | 0.02153052 | 0.054607037 | 1 |
| BP | GO:0015980 | energy derivation by oxidation of organic compounds | 278/18862 | 0.022358449 | 0.05582811 | 3 |
| BP | GO:0050708 | regulation of protein secretion | 279/18862 | 0.022567937 | 0.05582811 | 3 |
| BP | GO:1990542 | mitochondrial transmembrane transport | 109/18862 | 0.023435072 | 0.05582811 | 2 |
| BP | GO:0006002 | fructose 6-phosphate metabolic process | 11/18862 | 0.02365853 | 0.05582811 | 1 |
| BP | GO:0006048 | UDP-N-acetylglucosamine biosynthetic process | 11/18862 | 0.02365853 | 0.05582811 | 1 |
| BP | GO:0006558 | L-phenylalanine metabolic process | 11/18862 | 0.02365853 | 0.05582811 | 1 |
| BP | GO:0006559 | L-phenylalanine catabolic process | 11/18862 | 0.02365853 | 0.05582811 | 1 |
| BP | GO:0006751 | glutathione catabolic process | 11/18862 | 0.02365853 | 0.05582811 | 1 |
| BP | GO:0010269 | response to selenium ion | 11/18862 | 0.02365853 | 0.05582811 | 1 |
| BP | GO:0033197 | response to vitamin E | 11/18862 | 0.02365853 | 0.05582811 | 1 |
| BP | GO:0033212 | iron import into cell | 11/18862 | 0.02365853 | 0.05582811 | 1 |
| BP | GO:0034969 | histone arginine methylation | 11/18862 | 0.02365853 | 0.05582811 | 1 |
| BP | GO:0042416 | dopamine biosynthetic process | 11/18862 | 0.02365853 | 0.05582811 | 1 |
| BP | GO:0070189 | kynurenine metabolic process | 11/18862 | 0.02365853 | 0.05582811 | 1 |
| BP | GO:0070778 | L-aspartate transmembrane transport | 11/18862 | 0.02365853 | 0.05582811 | 1 |
| BP | GO:1901660 | calcium ion export | 11/18862 | 0.02365853 | 0.05582811 | 1 |
| BP | GO:1902221 | erythrose 4-phosphate/phosphoenolpyruvate family amino acid metabolic process | 11/18862 | 0.02365853 | 0.05582811 | 1 |
| BP | GO:1902222 | erythrose 4-phosphate/phosphoenolpyruvate family amino acid catabolic process | 11/18862 | 0.02365853 | 0.05582811 | 1 |
| BP | GO:2000551 | regulation of T-helper 2 cell cytokine production | 11/18862 | 0.02365853 | 0.05582811 | 1 |
| BP | GO:0051341 | regulation of oxidoreductase activity | 110/18862 | 0.023836631 | 0.056043098 | 2 |
| BP | GO:0001909 | leukocyte mediated cytotoxicity | 113/18862 | 0.025058303 | 0.056686184 | 2 |
| BP | GO:0006554 | lysine catabolic process | 12/18862 | 0.025782025 | 0.056686184 | 1 |
| BP | GO:0006563 | L-serine metabolic process | 12/18862 | 0.025782025 | 0.056686184 | 1 |
| BP | GO:0006568 | tryptophan metabolic process | 12/18862 | 0.025782025 | 0.056686184 | 1 |
| BP | GO:0006570 | tyrosine metabolic process | 12/18862 | 0.025782025 | 0.056686184 | 1 |
| BP | GO:0015809 | arginine transport | 12/18862 | 0.025782025 | 0.056686184 | 1 |
| BP | GO:0034372 | very-low-density lipoprotein particle remodeling | 12/18862 | 0.025782025 | 0.056686184 | 1 |
| BP | GO:0035672 | oligopeptide transmembrane transport | 12/18862 | 0.025782025 | 0.056686184 | 1 |
| BP | GO:0043455 | regulation of secondary metabolic process | 12/18862 | 0.025782025 | 0.056686184 | 1 |
| BP | GO:0048021 | regulation of melanin biosynthetic process | 12/18862 | 0.025782025 | 0.056686184 | 1 |
| BP | GO:0051001 | negative regulation of nitric-oxide synthase activity | 12/18862 | 0.025782025 | 0.056686184 | 1 |
| BP | GO:0051340 | regulation of ligase activity | 12/18862 | 0.025782025 | 0.056686184 | 1 |
| BP | GO:0070857 | regulation of bile acid biosynthetic process | 12/18862 | 0.025782025 | 0.056686184 | 1 |
| BP | GO:0071872 | cellular response to epinephrine stimulus | 12/18862 | 0.025782025 | 0.056686184 | 1 |
| BP | GO:0072578 | neurotransmitter-gated ion channel clustering | 12/18862 | 0.025782025 | 0.056686184 | 1 |
| BP | GO:0106070 | regulation of adenylate cyclase-activating G protein-coupled receptor signaling pathway | 12/18862 | 0.025782025 | 0.056686184 | 1 |
| BP | GO:1900376 | regulation of secondary metabolite biosynthetic process | 12/18862 | 0.025782025 | 0.056686184 | 1 |
| BP | GO:1903909 | regulation of receptor clustering | 12/18862 | 0.025782025 | 0.056686184 | 1 |
| BP | GO:2001171 | positive regulation of ATP biosynthetic process | 12/18862 | 0.025782025 | 0.056686184 | 1 |
| BP | GO:0034599 | cellular response to oxidative stress | 299/18862 | 0.02698477 | 0.057981054 | 3 |
| BP | GO:0046879 | hormone secretion | 302/18862 | 0.027684581 | 0.057981054 | 3 |
| BP | GO:0002829 | negative regulation of type 2 immune response | 13/18862 | 0.027901014 | 0.057981054 | 1 |
| BP | GO:0006553 | lysine metabolic process | 13/18862 | 0.027901014 | 0.057981054 | 1 |
| BP | GO:0006596 | polyamine biosynthetic process | 13/18862 | 0.027901014 | 0.057981054 | 1 |
| BP | GO:0006857 | oligopeptide transport | 13/18862 | 0.027901014 | 0.057981054 | 1 |
| BP | GO:0009071 | serine family amino acid catabolic process | 13/18862 | 0.027901014 | 0.057981054 | 1 |
| BP | GO:0014745 | negative regulation of muscle adaptation | 13/18862 | 0.027901014 | 0.057981054 | 1 |
| BP | GO:0019336 | phenol-containing compound catabolic process | 13/18862 | 0.027901014 | 0.057981054 | 1 |
| BP | GO:0031282 | regulation of guanylate cyclase activity | 13/18862 | 0.027901014 | 0.057981054 | 1 |
| BP | GO:0034616 | response to laminar fluid shear stress | 13/18862 | 0.027901014 | 0.057981054 | 1 |
| BP | GO:0035745 | T-helper 2 cell cytokine production | 13/18862 | 0.027901014 | 0.057981054 | 1 |
| BP | GO:0038183 | bile acid signaling pathway | 13/18862 | 0.027901014 | 0.057981054 | 1 |
| BP | GO:0042762 | regulation of sulfur metabolic process | 13/18862 | 0.027901014 | 0.057981054 | 1 |
| BP | GO:0046349 | amino sugar biosynthetic process | 13/18862 | 0.027901014 | 0.057981054 | 1 |
| BP | GO:0050667 | homocysteine metabolic process | 13/18862 | 0.027901014 | 0.057981054 | 1 |
| BP | GO:1901070 | guanosine-containing compound biosynthetic process | 13/18862 | 0.027901014 | 0.057981054 | 1 |
| BP | GO:1900542 | regulation of purine nucleotide metabolic process | 120/18862 | 0.028006263 | 0.058012634 | 2 |
| BP | GO:0007568 | aging | 304/18862 | 0.028156519 | 0.058136941 | 3 |
| BP | GO:0019932 | second-messenger-mediated signaling | 307/18862 | 0.028872515 | 0.058587125 | 3 |
| BP | GO:0006140 | regulation of nucleotide metabolic process | 122/18862 | 0.0288731 | 0.058587125 | 2 |
| BP | GO:0002791 | regulation of peptide secretion | 308/18862 | 0.029113337 | 0.058587125 | 3 |
| BP | GO:0035821 | modulation of process of other organism | 123/18862 | 0.029310547 | 0.058587125 | 2 |
| BP | GO:0046887 | positive regulation of hormone secretion | 124/18862 | 0.029750661 | 0.058587125 | 2 |
| BP | GO:0001780 | neutrophil homeostasis | 14/18862 | 0.030015505 | 0.058587125 | 1 |
| BP | GO:0009415 | response to water | 14/18862 | 0.030015505 | 0.058587125 | 1 |
| BP | GO:0032310 | prostaglandin secretion | 14/18862 | 0.030015505 | 0.058587125 | 1 |
| BP | GO:0034370 | triglyceride-rich lipoprotein particle remodeling | 14/18862 | 0.030015505 | 0.058587125 | 1 |
| BP | GO:0046040 | IMP metabolic process | 14/18862 | 0.030015505 | 0.058587125 | 1 |
| BP | GO:0051775 | response to redox state | 14/18862 | 0.030015505 | 0.058587125 | 1 |
| BP | GO:0086103 | G protein-coupled receptor signaling pathway involved in heart process | 14/18862 | 0.030015505 | 0.058587125 | 1 |
| BP | GO:1901160 | primary amino compound metabolic process | 14/18862 | 0.030015505 | 0.058587125 | 1 |
| BP | GO:1903727 | positive regulation of phospholipid metabolic process | 14/18862 | 0.030015505 | 0.058587125 | 1 |
| BP | GO:1990822 | basic amino acid transmembrane transport | 14/18862 | 0.030015505 | 0.058587125 | 1 |
| BP | GO:0009895 | negative regulation of catabolic process | 312/18862 | 0.030087395 | 0.058587125 | 3 |
| BP | GO:0009914 | hormone transport | 312/18862 | 0.030087395 | 0.058587125 | 3 |
| BP | GO:0002705 | positive regulation of leukocyte mediated immunity | 125/18862 | 0.03019343 | 0.058587125 | 2 |
| BP | GO:0032355 | response to estradiol | 125/18862 | 0.03019343 | 0.058587125 | 2 |
| BP | GO:0048565 | digestive tract development | 125/18862 | 0.03019343 | 0.058587125 | 2 |
| BP | GO:0071333 | cellular response to glucose stimulus | 127/18862 | 0.031086881 | 0.05929969 | 2 |
| BP | GO:0050714 | positive regulation of protein secretion | 129/18862 | 0.031990794 | 0.05929969 | 2 |
| BP | GO:0071331 | cellular response to hexose stimulus | 129/18862 | 0.031990794 | 0.05929969 | 2 |
| BP | GO:0034612 | response to tumor necrosis factor | 320/18862 | 0.032087137 | 0.05929969 | 3 |
| BP | GO:0006089 | lactate metabolic process | 15/18862 | 0.03212551 | 0.05929969 | 1 |
| BP | GO:0006828 | manganese ion transport | 15/18862 | 0.03212551 | 0.05929969 | 1 |
| BP | GO:0018216 | peptidyl-arginine methylation | 15/18862 | 0.03212551 | 0.05929969 | 1 |
| BP | GO:0042559 | pteridine-containing compound biosynthetic process | 15/18862 | 0.03212551 | 0.05929969 | 1 |
| BP | GO:0046007 | negative regulation of activated T cell proliferation | 15/18862 | 0.03212551 | 0.05929969 | 1 |
| BP | GO:0046037 | GMP metabolic process | 15/18862 | 0.03212551 | 0.05929969 | 1 |
| BP | GO:0051938 | L-glutamate import | 15/18862 | 0.03212551 | 0.05929969 | 1 |
| BP | GO:0070885 | negative regulation of calcineurin-NFAT signaling cascade | 15/18862 | 0.03212551 | 0.05929969 | 1 |
| BP | GO:0071371 | cellular response to gonadotropin stimulus | 15/18862 | 0.03212551 | 0.05929969 | 1 |
| BP | GO:0071871 | response to epinephrine | 15/18862 | 0.03212551 | 0.05929969 | 1 |
| BP | GO:0098712 | L-glutamate import across plasma membrane | 15/18862 | 0.03212551 | 0.05929969 | 1 |
| BP | GO:0106057 | negative regulation of calcineurin-mediated signaling | 15/18862 | 0.03212551 | 0.05929969 | 1 |
| BP | GO:1904251 | regulation of bile acid metabolic process | 15/18862 | 0.03212551 | 0.05929969 | 1 |
| BP | GO:0046683 | response to organophosphorus | 130/18862 | 0.032446642 | 0.059551192 | 2 |
| BP | GO:0071326 | cellular response to monosaccharide stimulus | 130/18862 | 0.032446642 | 0.059551192 | 2 |
| BP | GO:0006047 | UDP-N-acetylglucosamine metabolic process | 16/18862 | 0.034231036 | 0.061714074 | 1 |
| BP | GO:0035729 | cellular response to hepatocyte growth factor stimulus | 16/18862 | 0.034231036 | 0.061714074 | 1 |
| BP | GO:0048311 | mitochondrion distribution | 16/18862 | 0.034231036 | 0.061714074 | 1 |
| BP | GO:0070365 | hepatocyte differentiation | 16/18862 | 0.034231036 | 0.061714074 | 1 |
| BP | GO:0097067 | cellular response to thyroid hormone stimulus | 16/18862 | 0.034231036 | 0.061714074 | 1 |
| BP | GO:1903204 | negative regulation of oxidative stress-induced neuron death | 16/18862 | 0.034231036 | 0.061714074 | 1 |
| BP | GO:0055123 | digestive system development | 134/18862 | 0.034295681 | 0.061714074 | 2 |
| BP | GO:0048545 | response to steroid hormone | 330/18862 | 0.034683301 | 0.062209779 | 3 |
| BP | GO:0042542 | response to hydrogen peroxide | 135/18862 | 0.034764288 | 0.062209779 | 2 |
| BP | GO:0071322 | cellular response to carbohydrate stimulus | 138/18862 | 0.036185144 | 0.062749376 | 2 |
| BP | GO:1903038 | negative regulation of leukocyte cell-cell adhesion | 138/18862 | 0.036185144 | 0.062749376 | 2 |
| BP | GO:0000097 | sulfur amino acid biosynthetic process | 17/18862 | 0.036332094 | 0.062749376 | 1 |
| BP | GO:0002888 | positive regulation of myeloid leukocyte mediated immunity | 17/18862 | 0.036332094 | 0.062749376 | 1 |
| BP | GO:0006586 | indolalkylamine metabolic process | 17/18862 | 0.036332094 | 0.062749376 | 1 |
| BP | GO:0006595 | polyamine metabolic process | 17/18862 | 0.036332094 | 0.062749376 | 1 |
| BP | GO:0010612 | regulation of cardiac muscle adaptation | 17/18862 | 0.036332094 | 0.062749376 | 1 |
| BP | GO:0015732 | prostaglandin transport | 17/18862 | 0.036332094 | 0.062749376 | 1 |
| BP | GO:0021756 | striatum development | 17/18862 | 0.036332094 | 0.062749376 | 1 |
| BP | GO:0031643 | positive regulation of myelination | 17/18862 | 0.036332094 | 0.062749376 | 1 |
| BP | GO:0055089 | fatty acid homeostasis | 17/18862 | 0.036332094 | 0.062749376 | 1 |
| BP | GO:0071071 | regulation of phospholipid biosynthetic process | 17/18862 | 0.036332094 | 0.062749376 | 1 |
| BP | GO:1903242 | regulation of cardiac muscle hypertrophy in response to stress | 17/18862 | 0.036332094 | 0.062749376 | 1 |
| BP | GO:0044344 | cellular response to fibroblast growth factor stimulus | 142/18862 | 0.038114229 | 0.064469186 | 2 |
| BP | GO:0032769 | negative regulation of monooxygenase activity | 18/18862 | 0.038428691 | 0.064469186 | 1 |
| BP | GO:0033189 | response to vitamin A | 18/18862 | 0.038428691 | 0.064469186 | 1 |
| BP | GO:0035728 | response to hepatocyte growth factor | 18/18862 | 0.038428691 | 0.064469186 | 1 |
| BP | GO:0035743 | CD4-positive, alpha-beta T cell cytokine production | 18/18862 | 0.038428691 | 0.064469186 | 1 |
| BP | GO:0045019 | negative regulation of nitric oxide biosynthetic process | 18/18862 | 0.038428691 | 0.064469186 | 1 |
| BP | GO:0071498 | cellular response to fluid shear stress | 18/18862 | 0.038428691 | 0.064469186 | 1 |
| BP | GO:0071605 | monocyte chemotactic protein-1 production | 18/18862 | 0.038428691 | 0.064469186 | 1 |
| BP | GO:0071637 | regulation of monocyte chemotactic protein-1 production | 18/18862 | 0.038428691 | 0.064469186 | 1 |
| BP | GO:1902548 | negative regulation of cellular response to vascular endothelial growth factor stimulus | 18/18862 | 0.038428691 | 0.064469186 | 1 |
| BP | GO:1904406 | negative regulation of nitric oxide metabolic process | 18/18862 | 0.038428691 | 0.064469186 | 1 |
| BP | GO:0014074 | response to purine-containing compound | 144/18862 | 0.039093382 | 0.065413944 | 2 |
| BP | GO:0062197 | cellular response to chemical stress | 347/18862 | 0.039341234 | 0.065658127 | 3 |
| BP | GO:0001678 | cellular glucose homeostasis | 145/18862 | 0.039586568 | 0.065896858 | 2 |
| BP | GO:0006144 | purine nucleobase metabolic process | 19/18862 | 0.040520839 | 0.066422267 | 1 |
| BP | GO:0006544 | glycine metabolic process | 19/18862 | 0.040520839 | 0.066422267 | 1 |
| BP | GO:0010988 | regulation of low-density lipoprotein particle clearance | 19/18862 | 0.040520839 | 0.066422267 | 1 |
| BP | GO:0034755 | iron ion transmembrane transport | 19/18862 | 0.040520839 | 0.066422267 | 1 |
| BP | GO:0046653 | tetrahydrofolate metabolic process | 19/18862 | 0.040520839 | 0.066422267 | 1 |
| BP | GO:0051900 | regulation of mitochondrial depolarization | 19/18862 | 0.040520839 | 0.066422267 | 1 |
| BP | GO:0071774 | response to fibroblast growth factor | 148/18862 | 0.041080419 | 0.067168625 | 2 |
| BP | GO:0002793 | positive regulation of peptide secretion | 151/18862 | 0.042595454 | 0.068111347 | 2 |
| BP | GO:0006925 | inflammatory cell apoptotic process | 20/18862 | 0.042608545 | 0.068111347 | 1 |
| BP | GO:0009713 | catechol-containing compound biosynthetic process | 20/18862 | 0.042608545 | 0.068111347 | 1 |
| BP | GO:0010042 | response to manganese ion | 20/18862 | 0.042608545 | 0.068111347 | 1 |
| BP | GO:0042423 | catecholamine biosynthetic process | 20/18862 | 0.042608545 | 0.068111347 | 1 |
| BP | GO:0046655 | folic acid metabolic process | 20/18862 | 0.042608545 | 0.068111347 | 1 |
| BP | GO:0050849 | negative regulation of calcium-mediated signaling | 20/18862 | 0.042608545 | 0.068111347 | 1 |
| BP | GO:0098703 | calcium ion import across plasma membrane | 20/18862 | 0.042608545 | 0.068111347 | 1 |
| BP | GO:1903206 | negative regulation of hydrogen peroxide-induced cell death | 20/18862 | 0.042608545 | 0.068111347 | 1 |
| BP | GO:0019370 | leukotriene biosynthetic process | 21/18862 | 0.044691819 | 0.07056603 | 1 |
| BP | GO:0042438 | melanin biosynthetic process | 21/18862 | 0.044691819 | 0.07056603 | 1 |
| BP | GO:1902656 | calcium ion import into cytosol | 21/18862 | 0.044691819 | 0.07056603 | 1 |
| BP | GO:2000479 | regulation of cAMP-dependent protein kinase activity | 21/18862 | 0.044691819 | 0.07056603 | 1 |
| BP | GO:2001169 | regulation of ATP biosynthetic process | 21/18862 | 0.044691819 | 0.07056603 | 1 |
| BP | GO:0007159 | leukocyte cell-cell adhesion | 366/18862 | 0.044907471 | 0.070733167 | 3 |
| BP | GO:0009306 | protein secretion | 367/18862 | 0.045210856 | 0.07103734 | 3 |
| BP | GO:0035592 | establishment of protein localization to extracellular region | 368/18862 | 0.045515277 | 0.071341656 | 3 |
| BP | GO:0034614 | cellular response to reactive oxygen species | 159/18862 | 0.04673666 | 0.071398478 | 2 |
| BP | GO:0002710 | negative regulation of T cell mediated immunity | 22/18862 | 0.04677067 | 0.071398478 | 1 |
| BP | GO:0006582 | melanin metabolic process | 22/18862 | 0.04677067 | 0.071398478 | 1 |
| BP | GO:0031281 | positive regulation of cyclase activity | 22/18862 | 0.04677067 | 0.071398478 | 1 |
| BP | GO:0042451 | purine nucleoside biosynthetic process | 22/18862 | 0.04677067 | 0.071398478 | 1 |
| BP | GO:0042455 | ribonucleoside biosynthetic process | 22/18862 | 0.04677067 | 0.071398478 | 1 |
| BP | GO:0043457 | regulation of cellular respiration | 22/18862 | 0.04677067 | 0.071398478 | 1 |
| BP | GO:0046129 | purine ribonucleoside biosynthetic process | 22/18862 | 0.04677067 | 0.071398478 | 1 |
| BP | GO:0051882 | mitochondrial depolarization | 22/18862 | 0.04677067 | 0.071398478 | 1 |
| BP | GO:1901032 | negative regulation of response to reactive oxygen species | 22/18862 | 0.04677067 | 0.071398478 | 1 |
| BP | GO:1903203 | regulation of oxidative stress-induced neuron death | 22/18862 | 0.04677067 | 0.071398478 | 1 |
| BP | GO:0043467 | regulation of generation of precursor metabolites and energy | 160/18862 | 0.047264439 | 0.071981677 | 2 |
| BP | GO:0071692 | protein localization to extracellular region | 375/18862 | 0.047675134 | 0.072194431 | 3 |
| BP | GO:0032635 | interleukin-6 production | 162/18862 | 0.048326622 | 0.072194431 | 2 |
| BP | GO:0009068 | aspartate family amino acid catabolic process | 23/18862 | 0.048845107 | 0.072194431 | 1 |
| BP | GO:0009226 | nucleotide-sugar biosynthetic process | 23/18862 | 0.048845107 | 0.072194431 | 1 |
| BP | GO:0021544 | subpallium development | 23/18862 | 0.048845107 | 0.072194431 | 1 |
| BP | GO:0033032 | regulation of myeloid cell apoptotic process | 23/18862 | 0.048845107 | 0.072194431 | 1 |
| BP | GO:0036499 | PERK-mediated unfolded protein response | 23/18862 | 0.048845107 | 0.072194431 | 1 |
| BP | GO:0044550 | secondary metabolite biosynthetic process | 23/18862 | 0.048845107 | 0.072194431 | 1 |
| BP | GO:0046628 | positive regulation of insulin receptor signaling pathway | 23/18862 | 0.048845107 | 0.072194431 | 1 |
| BP | GO:0051349 | positive regulation of lyase activity | 23/18862 | 0.048845107 | 0.072194431 | 1 |
| BP | GO:0071577 | zinc ion transmembrane transport | 23/18862 | 0.048845107 | 0.072194431 | 1 |
| BP | GO:0097066 | response to thyroid hormone | 23/18862 | 0.048845107 | 0.072194431 | 1 |
| BP | GO:0030324 | lung development | 163/18862 | 0.048861002 | 0.072194431 | 2 |
| CC | GO:0005759 | mitochondrial matrix | 476/19520 | 4.95E-08 | 3.28E-06 | 10 |
| CC | GO:0016323 | basolateral plasma membrane | 211/19520 | 0.00107667 | 0.035700119 | 4 |
| CC | GO:0009925 | basal plasma membrane | 240/19520 | 0.00172896 | 0.037293457 | 4 |
| CC | GO:0045178 | basal part of cell | 258/19520 | 0.002249447 | 0.037293457 | 4 |
| CC | GO:0005782 | peroxisomal matrix | 51/19520 | 0.005390201 | 0.059575905 | 2 |
| CC | GO:0031907 | microbody lumen | 51/19520 | 0.005390201 | 0.059575905 | 2 |
| CC | GO:0005741 | mitochondrial outer membrane | 195/19520 | 0.008465375 | 0.08019829 | 3 |
| CC | GO:0031968 | organelle outer membrane | 220/19520 | 0.011735608 | 0.088601336 | 3 |
| CC | GO:0019867 | outer membrane | 222/19520 | 0.012024467 | 0.088601336 | 3 |
| CC | GO:0099059 | integral component of presynaptic active zone membrane | 12/19520 | 0.02552341 | 0.169260509 | 1 |
| CC | GO:0098945 | intrinsic component of presynaptic active zone membrane | 15/19520 | 0.031804226 | 0.170381671 | 1 |
| CC | GO:0005777 | peroxisome | 136/19520 | 0.034601388 | 0.170381671 | 2 |
| CC | GO:0042579 | microbody | 136/19520 | 0.034601388 | 0.170381671 | 2 |
| CC | GO:0097449 | astrocyte projection | 17/19520 | 0.035969464 | 0.170381671 | 1 |
| CC | GO:0097228 | sperm principal piece | 22/19520 | 0.046306178 | 0.204722049 | 1 |
| MF | GO:0016879 | ligase activity, forming carbon-nitrogen bonds | 47/18337 | 2.65E-20 | 2.06E-18 | 11 |
| MF | GO:0016874 | ligase activity | 163/18337 | 4.89E-14 | 1.90E-12 | 11 |
| MF | GO:0016597 | amino acid binding | 54/18337 | 2.92E-13 | 7.57E-12 | 8 |
| MF | GO:0031406 | carboxylic acid binding | 184/18337 | 6.93E-12 | 1.35E-10 | 10 |
| MF | GO:0016884 | carbon-nitrogen ligase activity, with glutamine as amido-N-donor | 10/18337 | 1.08E-11 | 1.69E-10 | 5 |
| MF | GO:0043177 | organic acid binding | 114/18337 | 1.90E-07 | 2.47E-06 | 6 |
| MF | GO:0016813 | hydrolase activity, acting on carbon-nitrogen (but not peptide) bonds, in linear amidines | 11/18337 | 1.69E-06 | 1.88E-05 | 3 |
| MF | GO:0015179 | L-amino acid transmembrane transporter activity | 59/18337 | 8.96E-06 | 8.72E-05 | 4 |
| MF | GO:0016638 | oxidoreductase activity, acting on the CH-NH2 group of donors | 20/18337 | 1.15E-05 | 9.50E-05 | 3 |
| MF | GO:0008483 | transaminase activity | 21/18337 | 1.34E-05 | 9.50E-05 | 3 |
| MF | GO:0016881 | acid-amino acid ligase activity | 21/18337 | 1.34E-05 | 9.50E-05 | 3 |
| MF | GO:0016769 | transferase activity, transferring nitrogenous groups | 23/18337 | 1.78E-05 | 0.000115621 | 3 |
| MF | GO:0015171 | amino acid transmembrane transporter activity | 81/18337 | 3.16E-05 | 0.000189314 | 4 |
| MF | GO:0043531 | ADP binding | 39/18337 | 8.97E-05 | 0.000498858 | 3 |
| MF | GO:0016810 | hydrolase activity, acting on carbon-nitrogen (but not peptide) bonds | 117/18337 | 0.000132849 | 0.000689885 | 4 |
| MF | GO:0016840 | carbon-nitrogen lyase activity | 13/18337 | 0.000374568 | 0.001789027 | 2 |
| MF | GO:0046943 | carboxylic acid transmembrane transporter activity | 157/18337 | 0.00040927 | 0.001789027 | 4 |
| MF | GO:0005342 | organic acid transmembrane transporter activity | 158/18337 | 0.000419225 | 0.001789027 | 4 |
| MF | GO:0005313 | L-glutamate transmembrane transporter activity | 14/18337 | 0.000436378 | 0.001789027 | 2 |
| MF | GO:0015172 | acidic amino acid transmembrane transporter activity | 16/18337 | 0.000573815 | 0.002234858 | 2 |
| MF | GO:0008514 | organic anion transmembrane transporter activity | 177/18337 | 0.000643025 | 0.002385155 | 4 |
| MF | GO:0016646 | oxidoreductase activity, acting on the CH-NH group of donors, NAD or NADP as acceptor | 19/18337 | 0.00081422 | 0.002882883 | 2 |
| MF | GO:0015245 | fatty acid transmembrane transporter activity | 21/18337 | 0.000997092 | 0.003376879 | 2 |
| MF | GO:0016645 | oxidoreductase activity, acting on the CH-NH group of donors | 29/18337 | 0.001906018 | 0.006186197 | 2 |
| MF | GO:0015175 | neutral amino acid transmembrane transporter activity | 34/18337 | 0.002615138 | 0.008148219 | 2 |
| MF | GO:0008509 | anion transmembrane transporter activity | 459/18337 | 0.003429412 | 0.010274353 | 5 |
| MF | GO:0019842 | vitamin binding | 145/18337 | 0.004143765 | 0.011954721 | 3 |
| MF | GO:0051287 | NAD binding | 53/18337 | 0.006253712 | 0.017397545 | 2 |
| MF | GO:0030170 | pyridoxal phosphate binding | 55/18337 | 0.006720342 | 0.017449308 | 2 |
| MF | GO:0070279 | vitamin B6 binding | 55/18337 | 0.006720342 | 0.017449308 | 2 |
| MF | GO:0016829 | lyase activity | 194/18337 | 0.009252078 | 0.023248005 | 3 |
| MF | GO:0008028 | monocarboxylic acid transmembrane transporter activity | 69/18337 | 0.010409654 | 0.025339288 | 2 |
| MF | GO:0022853 | active ion transmembrane transporter activity | 206/18337 | 0.010884972 | 0.025693394 | 3 |
| MF | GO:0050660 | flavin adenine dinucleotide binding | 81/18337 | 0.0141362 | 0.03238634 | 2 |
| MF | GO:0015291 | secondary active transmembrane transporter activity | 239/18337 | 0.016192999 | 0.036038555 | 3 |
| MF | GO:0072341 | modified amino acid binding | 93/18337 | 0.01835357 | 0.039712402 | 2 |
| MF | GO:0015294 | solute:cation symporter activity | 99/18337 | 0.020637728 | 0.04311651 | 2 |
| MF | GO:0005381 | iron ion transmembrane transporter activity | 10/18337 | 0.022140911 | 0.04311651 | 1 |
| MF | GO:0015643 | toxic substance binding | 10/18337 | 0.022140911 | 0.04311651 | 1 |
| MF | GO:0018455 | alcohol dehydrogenase [NAD(P)+] activity | 10/18337 | 0.022140911 | 0.04311651 | 1 |
| MF | GO:0032052 | bile acid binding | 11/18337 | 0.024328515 | 0.046221055 | 1 |
| MF | GO:0005542 | folic acid binding | 12/18337 | 0.026511344 | 0.049168909 | 1 |
| MF | GO:0016616 | oxidoreductase activity, acting on the CH-OH group of donors, NAD or NADP as acceptor | 120/18337 | 0.029497286 | 0.052225816 | 2 |
| MF | GO:0022804 | active transmembrane transporter activity | 301/18337 | 0.029500529 | 0.052225816 | 3 |
| MF | GO:0050998 | nitric-oxide synthase binding | 14/18337 | 0.030862719 | 0.052261812 | 1 |
| MF | GO:0070403 | NAD+ binding | 14/18337 | 0.030862719 | 0.052261812 | 1 |
| MF | GO:0005283 | amino acid:sodium symporter activity | 15/18337 | 0.033031285 | 0.052721429 | 1 |
| MF | GO:0016614 | oxidoreductase activity, acting on CH-OH group of donors | 130/18337 | 0.034161031 | 0.052721429 | 2 |
| MF | GO:0010181 | FMN binding | 16/18337 | 0.035195116 | 0.052721429 | 1 |
| MF | GO:0015556 | C4-dicarboxylate transmembrane transporter activity | 16/18337 | 0.035195116 | 0.052721429 | 1 |
| MF | GO:0015662 | ion transmembrane transporter activity, phosphorylative mechanism | 16/18337 | 0.035195116 | 0.052721429 | 1 |
| MF | GO:0016641 | oxidoreductase activity, acting on the CH-NH2 group of donors, oxygen as acceptor | 16/18337 | 0.035195116 | 0.052721429 | 1 |
| MF | GO:0015174 | basic amino acid transmembrane transporter activity | 17/18337 | 0.037354223 | 0.053883285 | 1 |
| MF | GO:0046965 | retinoid X receptor binding | 17/18337 | 0.037354223 | 0.053883285 | 1 |
| MF | GO:0015355 | secondary active monocarboxylate transmembrane transporter activity | 18/18337 | 0.039508616 | 0.05399146 | 1 |
| MF | GO:0016755 | transferase activity, transferring amino-acyl groups | 18/18337 | 0.039508616 | 0.05399146 | 1 |
| MF | GO:0070122 | isopeptidase activity | 18/18337 | 0.039508616 | 0.05399146 | 1 |
| MF | GO:0015293 | symporter activity | 143/18337 | 0.040622169 | 0.054556089 | 2 |
| MF | GO:0005416 | amino acid:cation symporter activity | 19/18337 | 0.041658305 | 0.054999367 | 1 |
| MF | GO:0015295 | solute:proton symporter activity | 20/18337 | 0.043803299 | 0.056805799 | 1 |
| MF | GO:0005385 | zinc ion transmembrane transporter activity | 21/18337 | 0.045943609 | 0.056805799 | 1 |
| MF | GO:0015106 | bicarbonate transmembrane transporter activity | 21/18337 | 0.045943609 | 0.056805799 | 1 |
| MF | GO:0072349 | modified amino acid transmembrane transporter activity | 21/18337 | 0.045943609 | 0.056805799 | 1 |
| MF | GO:0015296 | anion:cation symporter activity | 22/18337 | 0.048079246 | 0.058517503 | 1 |

**Table S3b. Analysis of KEGG.**

| ID | Description | BgRatio | pvalue | qvalue | Count |
| --- | --- | --- | --- | --- | --- |
| hsa00330 | Arginine and proline metabolism | 54/5894 | 1.18E-17 | 2.85E-16 | 12 |
| hsa00250 | Alanine, aspartate and glutamate metabolism | 32/5894 | 1.66E-10 | 2.01E-09 | 7 |
| hsa00910 | Nitrogen metabolism | 23/5894 | 1.20E-09 | 9.71E-09 | 6 |
| hsa04964 | Proximal tubule bicarbonate reclamation | 23/5894 | 0.000217321 | 0.001315367 | 3 |
| hsa00340 | Histidine metabolism | 29/5894 | 0.000438904 | 0.002125219 | 3 |
| hsa00480 | Glutathione metabolism | 50/5894 | 0.002184817 | 0.008815929 | 3 |

# Appendix 4

**LASSO and** **SVM-RFE genes**

**Table S4a. LASSO genes.**

| FTCD | NAGS | PHGDH | HAL | ASNSD1 |
| --- | --- | --- | --- | --- |
| CPS1 | FPGS | GGT1 | ART4 | SLC38A1 |
| ADHFE1 | DDAH2 | GCLM | AADAT | GFPT2 |
| CTPS1 | SLC7A11 | GLUD1 | LGSN |  |

**Table S4b. SVM-RFE genes.**

| GCLM | FTCD | ASNSD1 | GGT1 | NAGS |
| --- | --- | --- | --- | --- |
| PHGDH | GFPT2 | DDAH2 | NR1H4 | AADAT |
| GLUD1 | SLC38A1 | CTPS1 | CPS1 | FAH |
| ART4 | ARG1 |  |  |  |

**Table S4c. InterGenes.**

| FTCD | NAGS | GGT1 | ART4 | SLC38A1 |
| --- | --- | --- | --- | --- |
| CPS1 | DDAH2 | GCLM | AADAT | GFPT2 |
| CTPS1 | PHGDH | GLUD1 | ASNSD1 |  |

# Appendix 5

**GSEA analysis**

**Table 5a. GGT1 of GSEA analysis.**

| ID | Description | setSize | NES | pvalue | qvalues | rank |
| --- | --- | --- | --- | --- | --- | --- |
| KEGG_LYSOSOME | KEGG_LYSOSOME | 116 | -2.604908491 | 1.00E-10 | 2.74E-09 | 3951 |
| KEGG_RIBOSOME | KEGG_RIBOSOME | 69 | -2.660017246 | 1.00E-10 | 2.74E-09 | 5166 |
| KEGG_UBIQUITIN_MEDIATED_PROTEOLYSIS | KEGG_UBIQUITIN_MEDIATED_PROTEOLYSIS | 125 | -2.588277985 | 1.00E-10 | 2.74E-09 | 5160 |
| KEGG_FOCAL_ADHESION | KEGG_FOCAL_ADHESION | 194 | -2.211584673 | 5.28E-10 | 1.08E-08 | 6404 |
| KEGG_HUNTINGTONS_DISEASE | KEGG_HUNTINGTONS_DISEASE | 152 | -2.258048682 | 8.37E-10 | 1.37E-08 | 5366 |
| KEGG_CHEMOKINE_SIGNALING_PATHWAY | KEGG_CHEMOKINE_SIGNALING_PATHWAY | 179 | -2.18030238 | 3.94E-09 | 5.39E-08 | 5305 |
| KEGG_OXIDATIVE_PHOSPHORYLATION | KEGG_OXIDATIVE_PHOSPHORYLATION | 95 | -2.315670133 | 1.27E-08 | 1.49E-07 | 4671 |
| KEGG_PROTEASOME | KEGG_PROTEASOME | 40 | -2.468144877 | 2.04E-08 | 1.95E-07 | 5282 |
| KEGG_ALZHEIMERS_DISEASE | KEGG_ALZHEIMERS_DISEASE | 138 | -2.191413739 | 2.14E-08 | 1.95E-07 | 5146 |
| KEGG_PATHWAYS_IN_CANCER | KEGG_PATHWAYS_IN_CANCER | 318 | -1.892389758 | 2.79E-08 | 2.29E-07 | 6617 |
| KEGG_SYSTEMIC_LUPUS_ERYTHEMATOSUS | KEGG_SYSTEMIC_LUPUS_ERYTHEMATOSUS | 50 | -2.380910976 | 6.76E-08 | 5.04E-07 | 3029 |
| KEGG_SPLICEOSOME | KEGG_SPLICEOSOME | 115 | -2.190595558 | 7.63E-08 | 5.22E-07 | 7145 |
| KEGG_T_CELL_RECEPTOR_SIGNALING_PATHWAY | KEGG_T_CELL_RECEPTOR_SIGNALING_PATHWAY | 107 | -2.192669333 | 1.26E-07 | 7.94E-07 | 5510 |
| KEGG_PARKINSONS_DISEASE | KEGG_PARKINSONS_DISEASE | 93 | -2.213649897 | 1.44E-07 | 8.43E-07 | 4134 |
| KEGG_ENDOCYTOSIS | KEGG_ENDOCYTOSIS | 170 | -2.052895448 | 2.36E-07 | 1.29E-06 | 3581 |
| KEGG_LEISHMANIA_INFECTION | KEGG_LEISHMANIA_INFECTION | 67 | -2.230878625 | 4.23E-07 | 2.17E-06 | 5459 |
| KEGG_NOD_LIKE_RECEPTOR_SIGNALING_PATHWAY | KEGG_NOD_LIKE_RECEPTOR_SIGNALING_PATHWAY | 60 | -2.26780179 | 5.81E-07 | 2.81E-06 | 4645 |
| KEGG_PATHOGENIC_ESCHERICHIA_COLI_INFECTION | KEGG_PATHOGENIC_ESCHERICHIA_COLI_INFECTION | 51 | -2.278120785 | 1.06E-06 | 4.82E-06 | 4482 |
| KEGG_COLORECTAL_CANCER | KEGG_COLORECTAL_CANCER | 62 | -2.188881078 | 1.43E-06 | 6.18E-06 | 5234 |
| KEGG_ECM_RECEPTOR_INTERACTION | KEGG_ECM_RECEPTOR_INTERACTION | 81 | -2.164177157 | 3.11E-06 | 1.28E-05 | 2775 |
| KEGG_OLFACTORY_TRANSDUCTION | KEGG_OLFACTORY_TRANSDUCTION | 108 | 1.994380995 | 4.67E-06 | 1.82E-05 | 7461 |
| KEGG_VIRAL_MYOCARDITIS | KEGG_VIRAL_MYOCARDITIS | 65 | -2.148815775 | 5.20E-06 | 1.94E-05 | 4759 |
| KEGG_AMINO_SUGAR_AND_NUCLEOTIDE_SUGAR_METABOLISM | KEGG_AMINO_SUGAR_AND_NUCLEOTIDE_SUGAR_METABOLISM | 40 | -2.196684626 | 5.50E-06 | 1.96E-05 | 5393 |
| KEGG_TOLL_LIKE_RECEPTOR_SIGNALING_PATHWAY | KEGG_TOLL_LIKE_RECEPTOR_SIGNALING_PATHWAY | 97 | -2.075192736 | 6.30E-06 | 2.08E-05 | 2961 |
| KEGG_PROTEIN_EXPORT | KEGG_PROTEIN_EXPORT | 22 | -2.269317418 | 6.33E-06 | 2.08E-05 | 4630 |
| KEGG_CELL_ADHESION_MOLECULES_CAMS | KEGG_CELL_ADHESION_MOLECULES_CAMS | 125 | -1.946606226 | 9.19E-06 | 2.85E-05 | 2905 |
| KEGG_APOPTOSIS | KEGG_APOPTOSIS | 87 | -2.020302169 | 9.38E-06 | 2.85E-05 | 5617 |
| KEGG_CHRONIC_MYELOID_LEUKEMIA | KEGG_CHRONIC_MYELOID_LEUKEMIA | 72 | -2.042028136 | 2.44E-05 | 7.14E-05 | 6281 |
| KEGG_REGULATION_OF_ACTIN_CYTOSKELETON | KEGG_REGULATION_OF_ACTIN_CYTOSKELETON | 208 | -1.779166833 | 2.98E-05 | 8.43E-05 | 6281 |
| KEGG_SMALL_CELL_LUNG_CANCER | KEGG_SMALL_CELL_LUNG_CANCER | 84 | -1.939227854 | 4.42E-05 | 0.000121058 | 4974 |
| KEGG_FC_GAMMA_R_MEDIATED_PHAGOCYTOSIS | KEGG_FC_GAMMA_R_MEDIATED_PHAGOCYTOSIS | 90 | -1.919145429 | 5.99E-05 | 0.000153781 | 5234 |
| KEGG_PANCREATIC_CANCER | KEGG_PANCREATIC_CANCER | 69 | -2.031568377 | 5.99E-05 | 0.000153781 | 6420 |
| KEGG_MAPK_SIGNALING_PATHWAY | KEGG_MAPK_SIGNALING_PATHWAY | 254 | -1.676739242 | 7.34E-05 | 0.000182612 | 5500 |
| KEGG_NEUROTROPHIN_SIGNALING_PATHWAY | KEGG_NEUROTROPHIN_SIGNALING_PATHWAY | 122 | -1.871400465 | 7.91E-05 | 0.000190183 | 7015 |
| KEGG_ADHERENS_JUNCTION | KEGG_ADHERENS_JUNCTION | 66 | -1.995237739 | 8.11E-05 | 0.000190183 | 4253 |
| KEGG_N_GLYCAN_BIOSYNTHESIS | KEGG_N_GLYCAN_BIOSYNTHESIS | 44 | -2.105372177 | 8.70E-05 | 0.000198394 | 4502 |
| KEGG_ALLOGRAFT_REJECTION | KEGG_ALLOGRAFT_REJECTION | 33 | -2.103369418 | 0.000113822 | 0.000252578 | 2349 |
| KEGG_EPITHELIAL_CELL_SIGNALING_IN_HELICOBACTER_PYLORI_INFECTION | KEGG_EPITHELIAL_CELL_SIGNALING_IN_HELICOBACTER_PYLORI_INFECTION | 67 | -1.90929165 | 0.000149939 | 0.000323967 | 6036 |
| KEGG_RNA_DEGRADATION | KEGG_RNA_DEGRADATION | 54 | -1.978380536 | 0.000235601 | 0.000496003 | 5377 |
| KEGG_RENAL_CELL_CARCINOMA | KEGG_RENAL_CELL_CARCINOMA | 66 | -1.923124553 | 0.00024406 | 0.000500965 | 6281 |
| KEGG_ENDOMETRIAL_CANCER | KEGG_ENDOMETRIAL_CANCER | 52 | -1.931496733 | 0.000290799 | 0.000582344 | 6301 |
| KEGG_PYRIMIDINE_METABOLISM | KEGG_PYRIMIDINE_METABOLISM | 91 | -1.839698311 | 0.000390934 | 0.000764233 | 4777 |
| KEGG_NEUROACTIVE_LIGAND_RECEPTOR_INTERACTION | KEGG_NEUROACTIVE_LIGAND_RECEPTOR_INTERACTION | 262 | 1.520196373 | 0.000425845 | 0.00081312 | 7814 |
| KEGG_LEUKOCYTE_TRANSENDOTHELIAL_MIGRATION | KEGG_LEUKOCYTE_TRANSENDOTHELIAL_MIGRATION | 112 | -1.800373066 | 0.000447464 | 0.000834981 | 3518 |
| KEGG_PURINE_METABOLISM | KEGG_PURINE_METABOLISM | 145 | -1.706757018 | 0.000507334 | 0.000925662 | 4805 |
| KEGG_PROSTATE_CANCER | KEGG_PROSTATE_CANCER | 89 | -1.812700715 | 0.000541412 | 0.000960162 | 6301 |
| KEGG_INSULIN_SIGNALING_PATHWAY | KEGG_INSULIN_SIGNALING_PATHWAY | 133 | -1.718467476 | 0.000549631 | 0.000960162 | 5537 |
| KEGG_MTOR_SIGNALING_PATHWAY | KEGG_MTOR_SIGNALING_PATHWAY | 50 | -1.870845637 | 0.000616778 | 0.001055016 | 5149 |
| KEGG_SPHINGOLIPID_METABOLISM | KEGG_SPHINGOLIPID_METABOLISM | 31 | -1.909191874 | 0.00064567 | 0.001073151 | 2101 |
| KEGG_VIBRIO_CHOLERAE_INFECTION | KEGG_VIBRIO_CHOLERAE_INFECTION | 51 | -1.864306741 | 0.000653522 | 0.001073151 | 5888 |
| KEGG_ACUTE_MYELOID_LEUKEMIA | KEGG_ACUTE_MYELOID_LEUKEMIA | 56 | -1.862251686 | 0.000671065 | 0.001080353 | 6302 |
| KEGG_CARDIAC_MUSCLE_CONTRACTION | KEGG_CARDIAC_MUSCLE_CONTRACTION | 70 | -1.810235968 | 0.000688447 | 0.001087021 | 5113 |
| KEGG_CELL_CYCLE | KEGG_CELL_CYCLE | 122 | -1.722168693 | 0.000704152 | 0.001090841 | 6191 |
| KEGG_WNT_SIGNALING_PATHWAY | KEGG_WNT_SIGNALING_PATHWAY | 146 | -1.643201801 | 0.000732934 | 0.001114402 | 4329 |
| KEGG_TYPE_I_DIABETES_MELLITUS | KEGG_TYPE_I_DIABETES_MELLITUS | 39 | -1.921757739 | 0.001239648 | 0.001850575 | 2349 |
| KEGG_B_CELL_RECEPTOR_SIGNALING_PATHWAY | KEGG_B_CELL_RECEPTOR_SIGNALING_PATHWAY | 75 | -1.736347211 | 0.00127382 | 0.001867631 | 5679 |
| KEGG_ARRHYTHMOGENIC_RIGHT_VENTRICULAR_CARDIOMYOPATHY_ARVC | KEGG_ARRHYTHMOGENIC_RIGHT_VENTRICULAR_CARDIOMYOPATHY_ARVC | 72 | -1.749172732 | 0.001347735 | 0.001941335 | 5113 |
| KEGG_CITRATE_CYCLE_TCA_CYCLE | KEGG_CITRATE_CYCLE_TCA_CYCLE | 30 | -1.836989758 | 0.00187744 | 0.002657719 | 1935 |
| KEGG_GAP_JUNCTION | KEGG_GAP_JUNCTION | 84 | -1.665952029 | 0.002018656 | 0.002809191 | 5029 |
| KEGG_NUCLEOTIDE_EXCISION_REPAIR | KEGG_NUCLEOTIDE_EXCISION_REPAIR | 43 | -1.8059147 | 0.002120674 | 0.002901975 | 4764 |
| KEGG_PROPANOATE_METABOLISM | KEGG_PROPANOATE_METABOLISM | 31 | -1.807923464 | 0.00223297 | 0.003005551 | 3773 |
| KEGG_PRION_DISEASES | KEGG_PRION_DISEASES | 34 | -1.798183044 | 0.002740086 | 0.003628637 | 4033 |
| KEGG_CIRCADIAN_RHYTHM_MAMMAL | KEGG_CIRCADIAN_RHYTHM_MAMMAL | 11 | -1.864278651 | 0.003558367 | 0.00463747 | 3068 |
| KEGG_PYRUVATE_METABOLISM | KEGG_PYRUVATE_METABOLISM | 39 | -1.817940194 | 0.003710758 | 0.004695361 | 2390 |
| KEGG_TGF_BETA_SIGNALING_PATHWAY | KEGG_TGF_BETA_SIGNALING_PATHWAY | 81 | -1.636932553 | 0.00375872 | 0.004695361 | 6155 |
| KEGG_MISMATCH_REPAIR | KEGG_MISMATCH_REPAIR | 22 | -1.809295809 | 0.003774348 | 0.004695361 | 1747 |
| KEGG_PEROXISOME | KEGG_PEROXISOME | 77 | -1.686598321 | 0.004233595 | 0.005188066 | 3992 |
| KEGG_OOCYTE_MEIOSIS | KEGG_OOCYTE_MEIOSIS | 107 | -1.598446629 | 0.004859993 | 0.005868102 | 5655 |
| KEGG_TIGHT_JUNCTION | KEGG_TIGHT_JUNCTION | 126 | -1.558579792 | 0.005223842 | 0.006216013 | 5094 |
| KEGG_INOSITOL_PHOSPHATE_METABOLISM | KEGG_INOSITOL_PHOSPHATE_METABOLISM | 54 | -1.695008447 | 0.005396559 | 0.006329798 | 7178 |
| KEGG_FC_EPSILON_RI_SIGNALING_PATHWAY | KEGG_FC_EPSILON_RI_SIGNALING_PATHWAY | 73 | -1.659380453 | 0.00619586 | 0.007108708 | 5679 |
| KEGG_RETINOL_METABOLISM | KEGG_RETINOL_METABOLISM | 45 | 1.625837122 | 0.006309417 | 0.007108708 | 3941 |
| KEGG_GRAFT_VERSUS_HOST_DISEASE | KEGG_GRAFT_VERSUS_HOST_DISEASE | 35 | -1.791380828 | 0.006320371 | 0.007108708 | 2349 |
| KEGG_COMPLEMENT_AND_COAGULATION_CASCADES | KEGG_COMPLEMENT_AND_COAGULATION_CASCADES | 67 | -1.610287579 | 0.00674907 | 0.007425155 | 2912 |
| KEGG_SNARE_INTERACTIONS_IN_VESICULAR_TRANSPORT | KEGG_SNARE_INTERACTIONS_IN_VESICULAR_TRANSPORT | 37 | -1.724588685 | 0.006782594 | 0.007425155 | 6475 |
| KEGG_P53_SIGNALING_PATHWAY | KEGG_P53_SIGNALING_PATHWAY | 65 | -1.638561798 | 0.006892643 | 0.007446345 | 5226 |
| KEGG_VALINE_LEUCINE_AND_ISOLEUCINE_DEGRADATION | KEGG_VALINE_LEUCINE_AND_ISOLEUCINE_DEGRADATION | 43 | -1.682621265 | 0.007846808 | 0.008367068 | 6180 |
| KEGG_VASCULAR_SMOOTH_MUSCLE_CONTRACTION | KEGG_VASCULAR_SMOOTH_MUSCLE_CONTRACTION | 103 | -1.523389608 | 0.008753046 | 0.009213733 | 5270 |
| KEGG_DILATED_CARDIOMYOPATHY | KEGG_DILATED_CARDIOMYOPATHY | 88 | -1.532099363 | 0.010245436 | 0.010608227 | 5168 |
| KEGG_CYTOKINE_CYTOKINE_RECEPTOR_INTERACTION | KEGG_CYTOKINE_CYTOKINE_RECEPTOR_INTERACTION | 250 | -1.379811617 | 0.010423868 | 0.010608227 | 4882 |
| KEGG_HYPERTROPHIC_CARDIOMYOPATHY_HCM | KEGG_HYPERTROPHIC_CARDIOMYOPATHY_HCM | 81 | -1.553568938 | 0.010465424 | 0.010608227 | 5168 |
| KEGG_PROGESTERONE_MEDIATED_OOCYTE_MATURATION | KEGG_PROGESTERONE_MEDIATED_OOCYTE_MATURATION | 83 | -1.566548824 | 0.011595922 | 0.011610808 | 5234 |
| KEGG_GLYCOLYSIS_GLUCONEOGENESIS | KEGG_GLYCOLYSIS_GLUCONEOGENESIS | 60 | -1.581702402 | 0.012477309 | 0.012342804 | 2800 |
| KEGG_INTESTINAL_IMMUNE_NETWORK_FOR_IGA_PRODUCTION | KEGG_INTESTINAL_IMMUNE_NETWORK_FOR_IGA_PRODUCTION | 44 | -1.648572284 | 0.013286905 | 0.0129872 | 2232 |
| KEGG_DNA_REPLICATION | KEGG_DNA_REPLICATION | 36 | -1.652500156 | 0.01344603 | 0.012988115 | 5685 |
| KEGG_DRUG_METABOLISM_CYTOCHROME_P450 | KEGG_DRUG_METABOLISM_CYTOCHROME_P450 | 58 | 1.545566109 | 0.014394147 | 0.01374227 | 4966 |
| KEGG_THYROID_CANCER | KEGG_THYROID_CANCER | 29 | -1.610910698 | 0.015462168 | 0.014592246 | 6580 |
| KEGG_NATURAL_KILLER_CELL_MEDIATED_CYTOTOXICITY | KEGG_NATURAL_KILLER_CELL_MEDIATED_CYTOTOXICITY | 128 | -1.433622165 | 0.016607103 | 0.015494666 | 4746 |
| KEGG_LYSINE_DEGRADATION | KEGG_LYSINE_DEGRADATION | 39 | -1.586567968 | 0.020229063 | 0.018560757 | 6563 |
| KEGG_ETHER_LIPID_METABOLISM | KEGG_ETHER_LIPID_METABOLISM | 25 | -1.65038628 | 0.020345446 | 0.018560757 | 2027 |
| KEGG_FRUCTOSE_AND_MANNOSE_METABOLISM | KEGG_FRUCTOSE_AND_MANNOSE_METABOLISM | 31 | -1.526000282 | 0.023918912 | 0.021580974 | 6071 |
| KEGG_STEROID_BIOSYNTHESIS | KEGG_STEROID_BIOSYNTHESIS | 16 | -1.651250969 | 0.024986957 | 0.022299572 | 3645 |
| KEGG_ERBB_SIGNALING_PATHWAY | KEGG_ERBB_SIGNALING_PATHWAY | 86 | -1.406750002 | 0.026061515 | 0.023008468 | 6301 |
| KEGG_LONG_TERM_POTENTIATION | KEGG_LONG_TERM_POTENTIATION | 68 | -1.466307069 | 0.028891422 | 0.025235508 | 3949 |
| KEGG_NOTCH_SIGNALING_PATHWAY | KEGG_NOTCH_SIGNALING_PATHWAY | 46 | -1.465995423 | 0.036619143 | 0.031648677 | 1976 |
| KEGG_MATURITY_ONSET_DIABETES_OF_THE_YOUNG | KEGG_MATURITY_ONSET_DIABETES_OF_THE_YOUNG | 23 | 1.534734066 | 0.040610834 | 0.034491063 | 7038 |
| KEGG_MELANOGENESIS | KEGG_MELANOGENESIS | 96 | -1.384232778 | 0.040748096 | 0.034491063 | 3922 |
| KEGG_ASTHMA | KEGG_ASTHMA | 26 | -1.555450894 | 0.041464035 | 0.034738934 | 2232 |
| KEGG_PHOSPHATIDYLINOSITOL_SIGNALING_SYSTEM | KEGG_PHOSPHATIDYLINOSITOL_SIGNALING_SYSTEM | 74 | -1.406902182 | 0.042355029 | 0.035030923 | 5577 |
| KEGG_AXON_GUIDANCE | KEGG_AXON_GUIDANCE | 128 | -1.330515668 | 0.042665868 | 0.035030923 | 4722 |
| KEGG_VEGF_SIGNALING_PATHWAY | KEGG_VEGF_SIGNALING_PATHWAY | 71 | -1.40057213 | 0.043235014 | 0.035146754 | 7072 |
| KEGG_PENTOSE_PHOSPHATE_PATHWAY | KEGG_PENTOSE_PHOSPHATE_PATHWAY | 26 | -1.546406467 | 0.043996142 | 0.035414851 | 2800 |
| KEGG_AMINOACYL_TRNA_BIOSYNTHESIS | KEGG_AMINOACYL_TRNA_BIOSYNTHESIS | 21 | -1.490985442 | 0.046333164 | 0.036933948 | 7048 |
| KEGG_VASOPRESSIN_REGULATED_WATER_REABSORPTION | KEGG_VASOPRESSIN_REGULATED_WATER_REABSORPTION | 44 | -1.469450674 | 0.049522295 | 0.038410541 | 6670 |
| KEGG_NON_SMALL_CELL_LUNG_CANCER | KEGG_NON_SMALL_CELL_LUNG_CANCER | 54 | -1.387122853 | 0.04952381 | 0.038410541 | 5679 |
| KEGG_GLYOXYLATE_AND_DICARBOXYLATE_METABOLISM | KEGG_GLYOXYLATE_AND_DICARBOXYLATE_METABOLISM | 16 | -1.554416763 | 0.049588993 | 0.038410541 | 1935 |

**Table 5b. GLUD1 of GSEA analysis.**

| ID | Description | setSize | NES | pvalue | qvalues | rank |
| --- | --- | --- | --- | --- | --- | --- |
| KEGG_NEUROACTIVE_LIGAND_RECEPTOR_INTERACTION | KEGG_NEUROACTIVE_LIGAND_RECEPTOR_INTERACTION | 262 | -2.27006335 | 1.00E-10 | 4.25E-09 | 5363 |
| KEGG_SPLICEOSOME | KEGG_SPLICEOSOME | 115 | 2.630196776 | 1.00E-10 | 4.25E-09 | 6284 |
| KEGG_UBIQUITIN_MEDIATED_PROTEOLYSIS | KEGG_UBIQUITIN_MEDIATED_PROTEOLYSIS | 125 | 2.475189901 | 1.00E-10 | 4.25E-09 | 5052 |
| KEGG_RNA_DEGRADATION | KEGG_RNA_DEGRADATION | 54 | 2.188426217 | 4.18E-06 | 0.000133081 | 4766 |
| KEGG_CELL_CYCLE | KEGG_CELL_CYCLE | 122 | 1.830346021 | 3.72E-05 | 0.000800861 | 6192 |
| KEGG_PROTEIN_EXPORT | KEGG_PROTEIN_EXPORT | 22 | 2.212541165 | 3.77E-05 | 0.000800861 | 7050 |
| KEGG_OLFACTORY_TRANSDUCTION | KEGG_OLFACTORY_TRANSDUCTION | 108 | -1.843981589 | 0.000245411 | 0.003734118 | 5304 |
| KEGG_ADHERENS_JUNCTION | KEGG_ADHERENS_JUNCTION | 66 | 1.874248442 | 0.000262889 | 0.003734118 | 7579 |
| KEGG_B_CELL_RECEPTOR_SIGNALING_PATHWAY | KEGG_B_CELL_RECEPTOR_SIGNALING_PATHWAY | 75 | 1.868532017 | 0.000263857 | 0.003734118 | 7016 |
| KEGG_NEUROTROPHIN_SIGNALING_PATHWAY | KEGG_NEUROTROPHIN_SIGNALING_PATHWAY | 122 | 1.687424336 | 0.000443381 | 0.005647278 | 6561 |
| KEGG_ENDOMETRIAL_CANCER | KEGG_ENDOMETRIAL_CANCER | 52 | 1.867611089 | 0.000757682 | 0.008773163 | 6474 |
| KEGG_ENDOCYTOSIS | KEGG_ENDOCYTOSIS | 170 | 1.595857247 | 0.000878087 | 0.008921821 | 4768 |
| KEGG_LYSOSOME | KEGG_LYSOSOME | 116 | 1.66920314 | 0.000937962 | 0.008921821 | 7276 |
| KEGG_PANCREATIC_CANCER | KEGG_PANCREATIC_CANCER | 69 | 1.797292895 | 0.000980663 | 0.008921821 | 6522 |
| KEGG_PATHOGENIC_ESCHERICHIA_COLI_INFECTION | KEGG_PATHOGENIC_ESCHERICHIA_COLI_INFECTION | 51 | 1.821931545 | 0.001190755 | 0.010110969 | 6414 |
| KEGG_RENAL_CELL_CARCINOMA | KEGG_RENAL_CELL_CARCINOMA | 66 | 1.722879168 | 0.001520361 | 0.011804638 | 6432 |
| KEGG_CHRONIC_MYELOID_LEUKEMIA | KEGG_CHRONIC_MYELOID_LEUKEMIA | 72 | 1.690029585 | 0.001575578 | 0.011804638 | 6522 |
| KEGG_COLORECTAL_CANCER | KEGG_COLORECTAL_CANCER | 62 | 1.773497859 | 0.001724593 | 0.012203257 | 5206 |
| KEGG_BASAL_TRANSCRIPTION_FACTORS | KEGG_BASAL_TRANSCRIPTION_FACTORS | 32 | 1.884397444 | 0.001959252 | 0.013134047 | 8082 |
| KEGG_N_GLYCAN_BIOSYNTHESIS | KEGG_N_GLYCAN_BIOSYNTHESIS | 44 | 1.811717035 | 0.002126152 | 0.013323086 | 5412 |
| KEGG_VALINE_LEUCINE_AND_ISOLEUCINE_DEGRADATION | KEGG_VALINE_LEUCINE_AND_ISOLEUCINE_DEGRADATION | 43 | 1.720269769 | 0.002196657 | 0.013323086 | 5719 |
| KEGG_OOCYTE_MEIOSIS | KEGG_OOCYTE_MEIOSIS | 107 | 1.618741866 | 0.00260946 | 0.015107397 | 4662 |
| KEGG_T_CELL_RECEPTOR_SIGNALING_PATHWAY | KEGG_T_CELL_RECEPTOR_SIGNALING_PATHWAY | 107 | 1.605032385 | 0.002959328 | 0.015796863 | 5508 |
| KEGG_PROTEASOME | KEGG_PROTEASOME | 40 | 1.773837852 | 0.002976599 | 0.015796863 | 6475 |
| KEGG_CALCIUM_SIGNALING_PATHWAY | KEGG_CALCIUM_SIGNALING_PATHWAY | 172 | -1.456905996 | 0.003615694 | 0.018421009 | 4911 |
| KEGG_PROSTATE_CANCER | KEGG_PROSTATE_CANCER | 89 | 1.59105883 | 0.003762789 | 0.018433096 | 5300 |
| KEGG_NUCLEOTIDE_EXCISION_REPAIR | KEGG_NUCLEOTIDE_EXCISION_REPAIR | 43 | 1.650186684 | 0.004033865 | 0.019029148 | 4992 |
| KEGG_HEDGEHOG_SIGNALING_PATHWAY | KEGG_HEDGEHOG_SIGNALING_PATHWAY | 53 | -1.72837107 | 0.004288942 | 0.019509848 | 3833 |
| KEGG_EPITHELIAL_CELL_SIGNALING_IN_HELICOBACTER_PYLORI_INFECTION | KEGG_EPITHELIAL_CELL_SIGNALING_IN_HELICOBACTER_PYLORI_INFECTION | 67 | 1.626835832 | 0.004837375 | 0.021245823 | 7055 |
| KEGG_CIRCADIAN_RHYTHM_MAMMAL | KEGG_CIRCADIAN_RHYTHM_MAMMAL | 11 | 1.79619104 | 0.005075718 | 0.021549538 | 4890 |
| KEGG_APOPTOSIS | KEGG_APOPTOSIS | 87 | 1.522229594 | 0.005773648 | 0.023721948 | 4654 |
| KEGG_GLYCOSPHINGOLIPID_BIOSYNTHESIS_LACTO_AND_NEOLACTO_SERIES | KEGG_GLYCOSPHINGOLIPID_BIOSYNTHESIS_LACTO_AND_NEOLACTO_SERIES | 25 | -1.729489873 | 0.006499553 | 0.02586993 | 1541 |
| KEGG_ASTHMA | KEGG_ASTHMA | 26 | -1.673170746 | 0.00756236 | 0.029188054 | 1786 |
| KEGG_OXIDATIVE_PHOSPHORYLATION | KEGG_OXIDATIVE_PHOSPHORYLATION | 95 | 1.518995385 | 0.008407887 | 0.031497038 | 7642 |
| KEGG_SMALL_CELL_LUNG_CANCER | KEGG_SMALL_CELL_LUNG_CANCER | 84 | 1.526640077 | 0.008892299 | 0.032359945 | 4935 |
| KEGG_PARKINSONS_DISEASE | KEGG_PARKINSONS_DISEASE | 93 | 1.559086653 | 0.00924563 | 0.032711148 | 7477 |
| KEGG_CITRATE_CYCLE_TCA_CYCLE | KEGG_CITRATE_CYCLE_TCA_CYCLE | 30 | 1.704888536 | 0.010138494 | 0.033297507 | 5205 |
| KEGG_NON_SMALL_CELL_LUNG_CANCER | KEGG_NON_SMALL_CELL_LUNG_CANCER | 54 | 1.625784826 | 0.010173359 | 0.033297507 | 6705 |
| KEGG_MATURITY_ONSET_DIABETES_OF_THE_YOUNG | KEGG_MATURITY_ONSET_DIABETES_OF_THE_YOUNG | 23 | -1.706009815 | 0.010195642 | 0.033297507 | 1965 |
| KEGG_GLYCOSYLPHOSPHATIDYLINOSITOL_GPI_ANCHOR_BIOSYNTHESIS | KEGG_GLYCOSYLPHOSPHATIDYLINOSITOL_GPI_ANCHOR_BIOSYNTHESIS | 24 | 1.641120361 | 0.013060132 | 0.04158621 | 6412 |
| KEGG_FC_GAMMA_R_MEDIATED_PHAGOCYTOSIS | KEGG_FC_GAMMA_R_MEDIATED_PHAGOCYTOSIS | 90 | 1.514615301 | 0.013611715 | 0.04228543 | 6705 |
| KEGG_LONG_TERM_POTENTIATION | KEGG_LONG_TERM_POTENTIATION | 68 | 1.545410116 | 0.015183 | 0.046043685 | 2091 |
| KEGG_SNARE_INTERACTIONS_IN_VESICULAR_TRANSPORT | KEGG_SNARE_INTERACTIONS_IN_VESICULAR_TRANSPORT | 37 | 1.634107366 | 0.016351305 | 0.048433485 | 7520 |
| KEGG_SPHINGOLIPID_METABOLISM | KEGG_SPHINGOLIPID_METABOLISM | 31 | 1.599752774 | 0.020603835 | 0.059541184 | 2901 |
| KEGG_PHOSPHATIDYLINOSITOL_SIGNALING_SYSTEM | KEGG_PHOSPHATIDYLINOSITOL_SIGNALING_SYSTEM | 74 | 1.489038379 | 0.021284589 | 0.059541184 | 5671 |
| KEGG_ALZHEIMERS_DISEASE | KEGG_ALZHEIMERS_DISEASE | 138 | 1.406888742 | 0.021793172 | 0.059541184 | 6297 |
| KEGG_MTOR_SIGNALING_PATHWAY | KEGG_MTOR_SIGNALING_PATHWAY | 50 | 1.591668898 | 0.021971189 | 0.059541184 | 4636 |
| KEGG_THYROID_CANCER | KEGG_THYROID_CANCER | 29 | 1.588987655 | 0.024314491 | 0.063291046 | 5206 |
| KEGG_WNT_SIGNALING_PATHWAY | KEGG_WNT_SIGNALING_PATHWAY | 146 | 1.40677408 | 0.024348745 | 0.063291046 | 5623 |
| KEGG_HUNTINGTONS_DISEASE | KEGG_HUNTINGTONS_DISEASE | 152 | 1.367258151 | 0.026364212 | 0.067159362 | 6548 |
| KEGG_PROGESTERONE_MEDIATED_OOCYTE_MATURATION | KEGG_PROGESTERONE_MEDIATED_OOCYTE_MATURATION | 83 | 1.459219284 | 0.027745436 | 0.069292006 | 6772 |
| KEGG_PEROXISOME | KEGG_PEROXISOME | 77 | 1.440589265 | 0.029778852 | 0.072940103 | 4332 |
| KEGG_ACUTE_MYELOID_LEUKEMIA | KEGG_ACUTE_MYELOID_LEUKEMIA | 56 | 1.522492241 | 0.031027965 | 0.073006961 | 6474 |
| KEGG_CYTOKINE_CYTOKINE_RECEPTOR_INTERACTION | KEGG_CYTOKINE_CYTOKINE_RECEPTOR_INTERACTION | 250 | -1.308057619 | 0.031484607 | 0.073006961 | 4682 |
| KEGG_COMPLEMENT_AND_COAGULATION_CASCADES | KEGG_COMPLEMENT_AND_COAGULATION_CASCADES | 67 | -1.421408771 | 0.031525733 | 0.073006961 | 5399 |
| KEGG_DNA_REPLICATION | KEGG_DNA_REPLICATION | 36 | 1.524944223 | 0.033381192 | 0.075923387 | 4845 |
| KEGG_VIBRIO_CHOLERAE_INFECTION | KEGG_VIBRIO_CHOLERAE_INFECTION | 51 | 1.46813614 | 0.035480759 | 0.079282953 | 7604 |
| KEGG_INSULIN_SIGNALING_PATHWAY | KEGG_INSULIN_SIGNALING_PATHWAY | 133 | 1.362840951 | 0.037410093 | 0.082152835 | 6493 |
| KEGG_TOLL_LIKE_RECEPTOR_SIGNALING_PATHWAY | KEGG_TOLL_LIKE_RECEPTOR_SIGNALING_PATHWAY | 97 | 1.363852202 | 0.03968254 | 0.085666143 | 6854 |
| KEGG_PROPANOATE_METABOLISM | KEGG_PROPANOATE_METABOLISM | 31 | 1.495806166 | 0.042466207 | 0.089231064 | 5690 |
| KEGG_FATTY_ACID_METABOLISM | KEGG_FATTY_ACID_METABOLISM | 40 | 1.459763016 | 0.042735043 | 0.089231064 | 6238 |
| KEGG_REGULATION_OF_ACTIN_CYTOSKELETON | KEGG_REGULATION_OF_ACTIN_CYTOSKELETON | 208 | 1.292294111 | 0.046898638 | 0.09634525 | 4709 |
| KEGG_INOSITOL_PHOSPHATE_METABOLISM | KEGG_INOSITOL_PHOSPHATE_METABOLISM | 54 | 1.463544761 | 0.049264447 | 0.099598966 | 5687 |

# Appendix 6

**Drug prediction**

**Table 6. Drug prediction.**

| search_term | gene | drug | interaction_types | sources |
| --- | --- | --- | --- | --- |
| CPS1 | CPS1 | CARGLUMIC ACID | positive modulator | ChemblInteractions |
| CPS1 | CPS1 | METHIONINE | unknown | PharmGKB |
| GGT1 | GGT1 | TAMOXIFEN | unknown | NCI |
| GGT1 | GGT1 | DITIOCARB | unknown | NCI |
| GGT1 | GGT1 | PIROXICAM | unknown | NCI |
| GGT1 | GGT1 | DICLOFENAC | unknown | NCI |
| GGT1 | GGT1 | CARBAMAZEPINE | unknown | NCI |
| GGT1 | GGT1 | ADRIAMYCIN | unknown | NCI |
| GGT1 | GGT1 | GENTAMICIN | unknown | NCI |
| GGT1 | GGT1 | AZACITIDINE | unknown | NCI |
| GGT1 | GGT1 | AMINOGLUTETHIMIDE | unknown | NCI |
| GGT1 | GGT1 | INDOMETHACIN | unknown | NCI |
| GGT1 | GGT1 | DISULFIRAM | unknown | NCI |
| GGT1 | GGT1 | MANNITOL | unknown | NCI |
| GGT1 | GGT1 | PHENYTOIN | unknown | NCI |
| GGT1 | GGT1 | MESTRANOL | unknown | NCI |
| GGT1 | GGT1 | CANNABINOL | unknown | NCI |
| GGT1 | GGT1 | CHEMBL1743358 | unknown | NCI |
| GGT1 | GGT1 | DEXAMETHASONE | unknown | NCI |
| GGT1 | GGT1 | LIOTHYRONINE SODIUM | unknown | NCI |
| GCLM | GCLM | CISPLATIN | unknown | NCI |
| GCLM | GCLM | SULFORAPHANE | unknown | NCI |
| GCLM | GCLM | MELATONIN | unknown | NCI |

# Appendix 7

**MiRNA and LncRNA**

**Table 7a. Gene-miRNA.**

| Gene | miRNA | miRanda | miRDB | TargetScan | Sum |
| --- | --- | --- | --- | --- | --- |
| SLC38A1 | hsa-miR-3148 | 1 | 1 | 1 | 3 |
| GCLM | hsa-miR-1197 | 1 | 1 | 1 | 3 |
| SLC38A1 | hsa-miR-548p | 1 | 1 | 1 | 3 |
| GCLM | hsa-miR-607 | 1 | 1 | 1 | 3 |
| SLC38A1 | hsa-miR-624-3p | 1 | 1 | 1 | 3 |
| GLUD1 | hsa-miR-548c-3p | 1 | 1 | 1 | 3 |
| NAGS | hsa-miR-3119 | 1 | 1 | 1 | 3 |
| AADAT | hsa-miR-557 | 1 | 1 | 1 | 3 |
| AADAT | hsa-miR-1303 | 1 | 1 | 1 | 3 |
| GLUD1 | hsa-miR-195-5p | 1 | 1 | 1 | 3 |
| GCLM | hsa-miR-708-3p | 1 | 1 | 1 | 3 |
| GCLM | hsa-miR-526b-5p | 1 | 1 | 1 | 3 |
| GFPT2 | hsa-miR-548a-3p | 1 | 1 | 1 | 3 |
| AADAT | hsa-miR-3187-3p | 1 | 1 | 1 | 3 |
| GLUD1 | hsa-miR-3119 | 1 | 1 | 1 | 3 |
| GCLM | hsa-miR-3148 | 1 | 1 | 1 | 3 |
| GFPT2 | hsa-miR-449b-3p | 1 | 1 | 1 | 3 |
| CPS1 | hsa-miR-195-3p | 1 | 1 | 1 | 3 |
| GCLM | hsa-miR-1279 | 1 | 1 | 1 | 3 |
| CPS1 | hsa-miR-1200 | 1 | 1 | 1 | 3 |
| CPS1 | hsa-miR-34a-3p | 1 | 1 | 1 | 3 |
| SLC38A1 | hsa-miR-892a | 1 | 1 | 1 | 3 |
| SLC38A1 | hsa-miR-4286 | 1 | 1 | 1 | 3 |
| GFPT2 | hsa-miR-3132 | 1 | 1 | 1 | 3 |
| GLUD1 | hsa-miR-4314 | 1 | 1 | 1 | 3 |
| SLC38A1 | hsa-miR-30c-5p | 1 | 1 | 1 | 3 |
| SLC38A1 | hsa-miR-182-3p | 1 | 1 | 1 | 3 |
| GCLM | hsa-miR-4279 | 1 | 1 | 1 | 3 |
| GLUD1 | hsa-miR-1184 | 1 | 1 | 1 | 3 |
| GFPT2 | hsa-miR-30d-5p | 1 | 1 | 1 | 3 |
| SLC38A1 | hsa-miR-3168 | 1 | 1 | 1 | 3 |
| GFPT2 | hsa-miR-340-5p | 1 | 1 | 1 | 3 |
| GCLM | hsa-miR-499a-3p | 1 | 1 | 1 | 3 |
| GFPT2 | hsa-miR-367-3p | 1 | 1 | 1 | 3 |
| CPS1 | hsa-miR-633 | 1 | 1 | 1 | 3 |
| SLC38A1 | hsa-miR-4263 | 1 | 1 | 1 | 3 |
| SLC38A1 | hsa-miR-4277 | 1 | 1 | 1 | 3 |
| GLUD1 | hsa-miR-30d-5p | 1 | 1 | 1 | 3 |
| AADAT | hsa-miR-590-3p | 1 | 1 | 1 | 3 |
| ASNSD1 | hsa-miR-606 | 1 | 1 | 1 | 3 |
| GFPT2 | hsa-miR-92b-3p | 1 | 1 | 1 | 3 |
| GLUD1 | hsa-miR-3148 | 1 | 1 | 1 | 3 |
| SLC38A1 | hsa-miR-3126-5p | 1 | 1 | 1 | 3 |
| GCLM | hsa-miR-9-5p | 1 | 1 | 1 | 3 |
| AADAT | hsa-miR-22-5p | 1 | 1 | 1 | 3 |
| ASNSD1 | hsa-miR-548x-3p | 1 | 1 | 1 | 3 |
| GCLM | hsa-miR-466 | 1 | 1 | 1 | 3 |
| SLC38A1 | hsa-miR-548d-3p | 1 | 1 | 1 | 3 |
| AADAT | hsa-miR-130a-5p | 1 | 1 | 1 | 3 |
| CPS1 | hsa-miR-373-5p | 1 | 1 | 1 | 3 |
| AADAT | hsa-miR-450b-5p | 1 | 1 | 1 | 3 |
| GFPT2 | hsa-miR-1197 | 1 | 1 | 1 | 3 |
| GLUD1 | hsa-miR-183-3p | 1 | 1 | 1 | 3 |
| GFPT2 | hsa-miR-363-3p | 1 | 1 | 1 | 3 |
| CPS1 | hsa-miR-16-2-3p | 1 | 1 | 1 | 3 |
| CPS1 | hsa-miR-637 | 1 | 1 | 1 | 3 |
| PHGDH | hsa-miR-3157-5p | 1 | 1 | 1 | 3 |
| SLC38A1 | hsa-miR-4308 | 1 | 1 | 1 | 3 |
| SLC38A1 | hsa-miR-527 | 1 | 1 | 1 | 3 |
| CPS1 | hsa-miR-574-5p | 1 | 1 | 1 | 3 |
| GFPT2 | hsa-miR-448 | 1 | 1 | 1 | 3 |
| GCLM | hsa-miR-3163 | 1 | 1 | 1 | 3 |
| SLC38A1 | hsa-miR-3163 | 1 | 1 | 1 | 3 |
| GLUD1 | hsa-miR-150-5p | 1 | 1 | 1 | 3 |
| GCLM | hsa-miR-3119 | 1 | 1 | 1 | 3 |
| GLUD1 | hsa-miR-760 | 1 | 1 | 1 | 3 |
| NAGS | hsa-miR-142-3p | 1 | 1 | 1 | 3 |
| SLC38A1 | hsa-miR-1202 | 1 | 1 | 1 | 3 |
| SLC38A1 | hsa-miR-3164 | 1 | 1 | 1 | 3 |
| SLC38A1 | hsa-miR-4261 | 1 | 1 | 1 | 3 |
| CPS1 | hsa-miR-363-5p | 1 | 1 | 1 | 3 |
| GFPT2 | hsa-miR-4279 | 1 | 1 | 1 | 3 |
| SLC38A1 | hsa-miR-432-5p | 1 | 1 | 1 | 3 |
| GLUD1 | hsa-miR-1185-5p | 1 | 1 | 1 | 3 |
| GFPT2 | hsa-miR-3121-3p | 1 | 1 | 1 | 3 |
| GCLM | hsa-miR-31-5p | 1 | 1 | 1 | 3 |
| GFPT2 | hsa-miR-32-5p | 1 | 1 | 1 | 3 |
| SLC38A1 | hsa-miR-4318 | 1 | 1 | 1 | 3 |
| SLC38A1 | hsa-miR-185-5p | 1 | 1 | 1 | 3 |
| CPS1 | hsa-miR-548n | 1 | 1 | 1 | 3 |
| SLC38A1 | hsa-miR-491-5p | 1 | 1 | 1 | 3 |
| GLUD1 | hsa-miR-30c-5p | 1 | 1 | 1 | 3 |
| CPS1 | hsa-miR-146a-3p | 1 | 1 | 1 | 3 |
| SLC38A1 | hsa-miR-4287 | 1 | 1 | 1 | 3 |
| GCLM | hsa-miR-1206 | 1 | 1 | 1 | 3 |
| GLUD1 | hsa-miR-466 | 1 | 1 | 1 | 3 |
| AADAT | hsa-miR-524-5p | 1 | 1 | 1 | 3 |
| GLUD1 | hsa-miR-424-5p | 1 | 1 | 1 | 3 |
| ASNSD1 | hsa-let-7f-1-3p | 1 | 1 | 1 | 3 |
| SLC38A1 | hsa-miR-3137 | 1 | 1 | 1 | 3 |
| SLC38A1 | hsa-miR-3135a | 1 | 1 | 1 | 3 |
| GLUD1 | hsa-miR-125a-3p | 1 | 1 | 1 | 3 |
| GLUD1 | hsa-miR-335-3p | 1 | 1 | 1 | 3 |
| GCLM | hsa-miR-186-5p | 1 | 1 | 1 | 3 |
| SLC38A1 | hsa-miR-486-5p | 1 | 1 | 1 | 3 |
| GLUD1 | hsa-miR-1224-5p | 1 | 1 | 1 | 3 |
| GFPT2 | hsa-miR-3125 | 1 | 1 | 1 | 3 |
| CPS1 | hsa-miR-3065-3p | 1 | 1 | 1 | 3 |
| SLC38A1 | hsa-miR-33a-3p | 1 | 1 | 1 | 3 |
| SLC38A1 | hsa-miR-23a-3p | 1 | 1 | 1 | 3 |
| ASNSD1 | hsa-miR-338-5p | 1 | 1 | 1 | 3 |
| ASNSD1 | hsa-let-7b-3p | 1 | 1 | 1 | 3 |
| GLUD1 | hsa-miR-1205 | 1 | 1 | 1 | 3 |
| CPS1 | hsa-miR-548a-5p | 1 | 1 | 1 | 3 |
| CPS1 | hsa-miR-1257 | 1 | 1 | 1 | 3 |
| ASNSD1 | hsa-let-7a-3p | 1 | 1 | 1 | 3 |
| CPS1 | hsa-miR-548b-5p | 1 | 1 | 1 | 3 |
| GLUD1 | hsa-miR-1244 | 1 | 1 | 1 | 3 |
| CPS1 | hsa-miR-29c-3p | 1 | 1 | 1 | 3 |
| SLC38A1 | hsa-miR-205-3p | 1 | 1 | 1 | 3 |
| DDAH2 | hsa-miR-659-3p | 1 | 1 | 1 | 3 |
| SLC38A1 | hsa-miR-943 | 1 | 1 | 1 | 3 |
| GLUD1 | hsa-miR-4311 | 1 | 1 | 1 | 3 |
| CPS1 | hsa-miR-29b-3p | 1 | 1 | 1 | 3 |
| GCLM | hsa-miR-4328 | 1 | 1 | 1 | 3 |
| GFPT2 | hsa-miR-1284 | 1 | 1 | 1 | 3 |
| SLC38A1 | hsa-miR-936 | 1 | 1 | 1 | 3 |
| GCLM | hsa-miR-576-5p | 1 | 1 | 1 | 3 |
| SLC38A1 | hsa-miR-2110 | 1 | 1 | 1 | 3 |
| GCLM | hsa-miR-126-5p | 1 | 1 | 1 | 3 |
| CPS1 | hsa-miR-559 | 1 | 1 | 1 | 3 |
| SLC38A1 | hsa-let-7a-3p | 1 | 1 | 1 | 3 |
| AADAT | hsa-miR-373-5p | 1 | 1 | 1 | 3 |
| GGT1 | hsa-miR-3150a-3p | 1 | 1 | 1 | 3 |
| GCLM | hsa-miR-145-5p | 1 | 1 | 1 | 3 |
| GGT1 | hsa-miR-4308 | 1 | 1 | 1 | 3 |
| CPS1 | hsa-miR-548t-5p | 1 | 1 | 1 | 3 |
| SLC38A1 | hsa-miR-518a-5p | 1 | 1 | 1 | 3 |
| GFPT2 | hsa-miR-506-3p | 1 | 1 | 1 | 3 |
| SLC38A1 | hsa-miR-518c-5p | 1 | 1 | 1 | 3 |
| SLC38A1 | hsa-miR-607 | 1 | 1 | 1 | 3 |
| GCLM | hsa-miR-205-3p | 1 | 1 | 1 | 3 |
| NAGS | hsa-miR-15a-3p | 1 | 1 | 1 | 3 |
| GLUD1 | hsa-miR-1208 | 1 | 1 | 1 | 3 |
| AADAT | hsa-miR-507 | 1 | 1 | 1 | 3 |
| GFPT2 | hsa-miR-30c-5p | 1 | 1 | 1 | 3 |
| CPS1 | hsa-miR-590-3p | 1 | 1 | 1 | 3 |
| AADAT | hsa-miR-4325 | 1 | 1 | 1 | 3 |
| CPS1 | hsa-miR-101-5p | 1 | 1 | 1 | 3 |
| GFPT2 | hsa-miR-508-3p | 1 | 1 | 1 | 3 |
| GLUD1 | hsa-miR-497-5p | 1 | 1 | 1 | 3 |
| GCLM | hsa-miR-590-3p | 1 | 1 | 1 | 3 |
| SLC38A1 | hsa-let-7b-3p | 1 | 1 | 1 | 3 |
| SLC38A1 | hsa-miR-4276 | 1 | 1 | 1 | 3 |
| CPS1 | hsa-miR-29a-3p | 1 | 1 | 1 | 3 |
| SLC38A1 | hsa-miR-148a-5p | 1 | 1 | 1 | 3 |
| SLC38A1 | hsa-miR-1182 | 1 | 1 | 1 | 3 |
| CPS1 | hsa-miR-3175 | 1 | 1 | 1 | 3 |
| SLC38A1 | hsa-miR-222-5p | 1 | 1 | 1 | 3 |
| CPS1 | hsa-miR-548i | 1 | 1 | 1 | 3 |
| CPS1 | hsa-miR-548c-5p | 1 | 1 | 1 | 3 |
| CPS1 | hsa-miR-4251 | 1 | 1 | 1 | 3 |
| SLC38A1 | hsa-miR-374b-3p | 1 | 1 | 1 | 3 |
| AADAT | hsa-miR-1972 | 1 | 1 | 1 | 3 |
| SLC38A1 | hsa-miR-199b-3p | 1 | 1 | 1 | 3 |
| SLC38A1 | hsa-miR-4271 | 1 | 1 | 1 | 3 |
| SLC38A1 | hsa-miR-3153 | 1 | 1 | 1 | 3 |
| GCLM | hsa-miR-944 | 1 | 1 | 1 | 3 |
| SLC38A1 | hsa-miR-199a-3p | 1 | 1 | 1 | 3 |
| NAGS | hsa-miR-2861 | 1 | 1 | 1 | 3 |
| GCLM | hsa-miR-374b-3p | 1 | 1 | 1 | 3 |
| ASNSD1 | hsa-miR-195-5p | 1 | 1 | 1 | 3 |
| SLC38A1 | hsa-miR-1229-3p | 1 | 1 | 1 | 3 |
| SLC38A1 | hsa-miR-643 | 1 | 1 | 1 | 3 |
| GLUD1 | hsa-miR-3163 | 1 | 1 | 1 | 3 |
| CPS1 | hsa-miR-3202 | 1 | 1 | 1 | 3 |
| CPS1 | hsa-miR-144-3p | 1 | 1 | 1 | 3 |
| SLC38A1 | hsa-miR-138-2-3p | 1 | 1 | 1 | 3 |
| PHGDH | hsa-miR-759 | 1 | 1 | 1 | 3 |
| CPS1 | hsa-miR-2054 | 1 | 1 | 1 | 3 |
| GCLM | hsa-miR-432-3p | 1 | 1 | 1 | 3 |
| ASNSD1 | hsa-miR-16-5p | 1 | 1 | 1 | 3 |
| AADAT | hsa-miR-129-5p | 1 | 1 | 1 | 3 |
| SLC38A1 | hsa-miR-30d-5p | 1 | 1 | 1 | 3 |
| GFPT2 | hsa-miR-513a-5p | 1 | 1 | 1 | 3 |
| GLUD1 | hsa-miR-30b-5p | 1 | 1 | 1 | 3 |
| SLC38A1 | hsa-miR-376a-5p | 1 | 1 | 1 | 3 |
| GLUD1 | hsa-miR-126-5p | 1 | 1 | 1 | 3 |
| SLC38A1 | hsa-miR-96-3p | 1 | 1 | 1 | 3 |
| SLC38A1 | hsa-miR-15b-3p | 1 | 1 | 1 | 3 |
| SLC38A1 | hsa-miR-508-3p | 1 | 1 | 1 | 3 |
| CPS1 | hsa-miR-198 | 1 | 1 | 1 | 3 |
| CPS1 | hsa-miR-767-5p | 1 | 1 | 1 | 3 |
| GFPT2 | hsa-miR-767-3p | 1 | 1 | 1 | 3 |
| SLC38A1 | hsa-miR-4254 | 1 | 1 | 1 | 3 |
| GLUD1 | hsa-miR-138-1-3p | 1 | 1 | 1 | 3 |
| GFPT2 | hsa-miR-25-3p | 1 | 1 | 1 | 3 |
| ASNSD1 | hsa-miR-424-5p | 1 | 1 | 1 | 3 |
| ASNSD1 | hsa-miR-200a-5p | 1 | 1 | 1 | 3 |
| GLUD1 | hsa-miR-935 | 1 | 1 | 1 | 3 |
| SLC38A1 | hsa-miR-1236-3p | 1 | 1 | 1 | 3 |
| SLC38A1 | hsa-miR-30b-5p | 1 | 1 | 1 | 3 |
| SLC38A1 | hsa-miR-578 | 1 | 1 | 1 | 3 |
| GCLM | hsa-miR-340-5p | 1 | 1 | 1 | 3 |
| GFPT2 | hsa-miR-935 | 1 | 1 | 1 | 3 |
| GFPT2 | hsa-miR-27a-5p | 1 | 1 | 1 | 3 |
| SLC38A1 | hsa-miR-146a-3p | 1 | 1 | 1 | 3 |
| GLUD1 | hsa-miR-524-5p | 1 | 1 | 1 | 3 |
| SLC38A1 | hsa-miR-30d-3p | 1 | 1 | 1 | 3 |
| GCLM | hsa-miR-570-3p | 1 | 1 | 1 | 3 |
| SLC38A1 | hsa-miR-32-3p | 1 | 1 | 1 | 3 |
| GCLM | hsa-miR-21-5p | 1 | 1 | 1 | 3 |
| GFPT2 | hsa-miR-30b-5p | 1 | 1 | 1 | 3 |
| SLC38A1 | hsa-miR-2113 | 1 | 1 | 1 | 3 |
| CPS1 | hsa-miR-3169 | 1 | 1 | 1 | 3 |
| GCLM | hsa-miR-183-5p | 1 | 1 | 1 | 3 |
| SLC38A1 | hsa-let-7f-1-3p | 1 | 1 | 1 | 3 |
| SLC38A1 | hsa-miR-599 | 1 | 1 | 1 | 3 |
| PHGDH | hsa-miR-1207-5p | 1 | 1 | 1 | 3 |
| SLC38A1 | hsa-miR-450b-5p | 1 | 1 | 1 | 3 |
| SLC38A1 | hsa-miR-944 | 1 | 1 | 1 | 3 |
| GLUD1 | hsa-miR-4328 | 1 | 1 | 1 | 3 |
| NAGS | hsa-miR-1914-5p | 1 | 1 | 1 | 3 |
| SLC38A1 | hsa-miR-342-3p | 1 | 1 | 1 | 3 |
| GLUD1 | hsa-miR-15b-5p | 1 | 1 | 1 | 3 |
| GCLM | hsa-miR-888-5p | 1 | 1 | 1 | 3 |
| SLC38A1 | hsa-miR-3201 | 1 | 1 | 1 | 3 |
| GLUD1 | hsa-miR-15a-5p | 1 | 1 | 1 | 3 |
| FTCD | hsa-miR-1207-5p | 1 | 1 | 1 | 3 |
| NAGS | hsa-miR-133b | 1 | 1 | 1 | 3 |
| CPS1 | hsa-miR-18a-3p | 1 | 1 | 1 | 3 |
| AADAT | hsa-miR-335-3p | 1 | 1 | 1 | 3 |
| CPS1 | hsa-miR-93-3p | 1 | 1 | 1 | 3 |
| SLC38A1 | hsa-miR-1286 | 1 | 1 | 1 | 3 |
| SLC38A1 | hsa-miR-3182 | 1 | 1 | 1 | 3 |
| SLC38A1 | hsa-miR-16-1-3p | 1 | 1 | 1 | 3 |
| NAGS | hsa-miR-615-5p | 1 | 1 | 1 | 3 |
| SLC38A1 | hsa-miR-100-3p | 1 | 1 | 1 | 3 |
| GLUD1 | hsa-miR-450b-5p | 1 | 1 | 1 | 3 |
| FTCD | hsa-miR-1827 | 1 | 1 | 1 | 3 |
| CPS1 | hsa-miR-24-3p | 1 | 1 | 1 | 3 |
| GCLM | hsa-miR-497-3p | 1 | 1 | 1 | 3 |
| SLC38A1 | hsa-miR-449b-3p | 1 | 1 | 1 | 3 |
| SLC38A1 | hsa-miR-3188 | 1 | 1 | 1 | 3 |
| SLC38A1 | hsa-miR-186-3p | 1 | 1 | 1 | 3 |
| GFPT2 | hsa-miR-27b-3p | 1 | 1 | 1 | 3 |
| SLC38A1 | hsa-miR-23b-3p | 1 | 1 | 1 | 3 |
| GLUD1 | hsa-miR-609 | 1 | 1 | 1 | 3 |
| GFPT2 | hsa-miR-27a-3p | 1 | 1 | 1 | 3 |
| SLC38A1 | hsa-miR-302e | 1 | 1 | 1 | 3 |
| AADAT | hsa-miR-548n | 1 | 1 | 1 | 3 |
| GLUD1 | hsa-miR-3152-3p | 1 | 1 | 1 | 3 |
| SLC38A1 | hsa-miR-2278 | 1 | 1 | 1 | 3 |
| GCLM | hsa-miR-335-3p | 1 | 1 | 1 | 3 |
| SLC38A1 | hsa-miR-548k | 1 | 1 | 1 | 3 |
| SLC38A1 | hsa-miR-3149 | 1 | 1 | 1 | 3 |
| GLUD1 | hsa-miR-452-5p | 1 | 1 | 1 | 3 |
| NAGS | hsa-miR-661 | 1 | 1 | 1 | 3 |
| SLC38A1 | hsa-miR-3065-5p | 1 | 1 | 1 | 3 |
| SLC38A1 | hsa-miR-3185 | 1 | 1 | 1 | 3 |
| GCLM | hsa-miR-1281 | 1 | 1 | 1 | 3 |
| NAGS | hsa-miR-1281 | 1 | 1 | 1 | 3 |
| GLUD1 | hsa-miR-185-5p | 1 | 1 | 1 | 3 |
| DDAH2 | hsa-miR-4310 | 1 | 1 | 1 | 3 |
| DDAH2 | hsa-miR-138-1-3p | 1 | 1 | 1 | 3 |
| GCLM | hsa-miR-548c-3p | 1 | 1 | 1 | 3 |
| GCLM | hsa-miR-590-5p | 1 | 1 | 1 | 3 |
| SLC38A1 | hsa-miR-758-3p | 1 | 1 | 1 | 3 |
| SLC38A1 | hsa-miR-569 | 1 | 1 | 1 | 3 |
| CPS1 | hsa-miR-3150a-3p | 1 | 1 | 1 | 3 |
| GCLM | hsa-miR-101-3p | 1 | 1 | 1 | 3 |
| GLUD1 | hsa-miR-298 | 1 | 1 | 1 | 3 |
| SLC38A1 | hsa-miR-3123 | 1 | 1 | 1 | 3 |
| SLC38A1 | hsa-miR-539-5p | 1 | 1 | 1 | 3 |
| SLC38A1 | hsa-miR-548x-3p | 1 | 1 | 1 | 3 |
| CPS1 | hsa-miR-548d-5p | 1 | 1 | 1 | 3 |
| CPS1 | hsa-miR-548l | 1 | 1 | 1 | 3 |
| AADAT | hsa-miR-548t-5p | 1 | 1 | 1 | 3 |
| CPS1 | hsa-miR-548w | 1 | 1 | 1 | 3 |
| GCLM | hsa-miR-587 | 1 | 1 | 1 | 3 |
| CPS1 | hsa-miR-16-1-3p | 1 | 1 | 1 | 3 |
| SLC38A1 | hsa-miR-1297 | 1 | 1 | 1 | 3 |
| SLC38A1 | hsa-miR-1207-3p | 1 | 1 | 1 | 3 |
| GLUD1 | hsa-miR-944 | 1 | 1 | 1 | 3 |
| GCLM | hsa-miR-129-5p | 1 | 1 | 1 | 3 |
| SLC38A1 | hsa-miR-548m | 1 | 1 | 1 | 3 |
| ASNSD1 | hsa-miR-548a-3p | 1 | 1 | 1 | 3 |
| GLUD1 | hsa-miR-374a-3p | 1 | 1 | 1 | 3 |
| GFPT2 | hsa-miR-1236-3p | 1 | 1 | 1 | 3 |
| SLC38A1 | hsa-miR-562 | 1 | 1 | 1 | 3 |
| CPS1 | hsa-miR-548h-5p | 1 | 1 | 1 | 3 |
| GLUD1 | hsa-miR-16-5p | 1 | 1 | 1 | 3 |
| GCLM | hsa-miR-650 | 1 | 1 | 1 | 3 |
| GLUD1 | hsa-miR-3120-3p | 1 | 1 | 1 | 3 |
| SLC38A1 | hsa-miR-593-3p | 1 | 1 | 1 | 3 |
| SLC38A1 | hsa-miR-628-5p | 1 | 1 | 1 | 3 |
| SLC38A1 | hsa-miR-218-5p | 1 | 1 | 1 | 3 |
| SLC38A1 | hsa-miR-505-3p | 1 | 1 | 1 | 3 |
| GFPT2 | hsa-miR-567 | 1 | 1 | 1 | 3 |
| ASNSD1 | hsa-miR-497-5p | 1 | 1 | 1 | 3 |
| SLC38A1 | hsa-miR-4291 | 1 | 1 | 1 | 3 |
| GCLM | hsa-miR-624-3p | 1 | 1 | 1 | 3 |
| GCLM | hsa-miR-562 | 1 | 1 | 1 | 3 |

**Table 7b. Gene-lncRNA.**

| miRNA | lncRNA |
| --- | --- |
| hsa-miR-1208 | FLJ16779 |
| hsa-miR-615-5p | MUC2 |
| hsa-miR-1200 | LINC01043 |
| hsa-miR-767-5p | RP11-326C3.10 |
| hsa-miR-125a-3p | RP11-10J21.4 |
| hsa-miR-570-3p | RP11-10J21.4 |
| hsa-miR-1202 | HP09025 |
| hsa-miR-1207-3p | MUC19 |
| hsa-miR-125a-3p | RP11-830F9.6 |
| hsa-miR-125a-3p | LINC00917 |
| hsa-miR-363-5p | RP11-573D15.8 |
| hsa-miR-27a-3p | RP11-10J21.4 |
| hsa-miR-218-5p | RP5-894D12.5 |
| hsa-miR-615-5p | PAX8-AS1 |
| hsa-miR-31-5p | C10orf91 |
| hsa-miR-145-5p | MUC19 |
| hsa-miR-198 | MUC19 |
| hsa-miR-125a-3p | AC097468.4 |
| hsa-miR-505-3p | AC079779.7 |
| hsa-miR-1972 | AC079779.7 |
| hsa-miR-363-5p | C10orf91 |
| hsa-miR-767-5p | RP11-326C3.14 |
| hsa-miR-24-3p | RP11-102K13.5 |
| hsa-miR-146a-3p | FAM74A1 |
| hsa-miR-146a-3p | RP11-830F9.6 |
| hsa-miR-342-3p | LL22NC03-27C5.1 |
| hsa-miR-125a-3p | CH507-216K13.2 |
| hsa-miR-1972 | RP11-102K13.5 |
| hsa-miR-1972 | RP5-894D12.5 |
| hsa-miR-185-5p | CTC-265F19.1 |
| hsa-miR-1972 | RP13-580B18.4 |
| hsa-let-7a-3p | RP3-323A16.1 |
| hsa-miR-185-5p | AC092657.2 |
| hsa-miR-186-5p | RP11-99L13.2 |
| hsa-miR-650 | RP11-138B4.1 |
| hsa-miR-1184 | TMEM191C |
| hsa-miR-1207-3p | RP11-326C3.10 |
| hsa-miR-146a-3p | FAM74A7 |
| hsa-miR-24-3p | LINC01106 |
| hsa-miR-624-3p | RP11-333E1.2 |
| hsa-miR-198 | RP11-830F9.6 |
| hsa-miR-18a-3p | RP3-388N13.3 |
| hsa-miR-499a-3p | RP4-737E23.2 |
| hsa-miR-1207-3p | RP11-326C3.14 |
| hsa-miR-146a-3p | FAM74A6 |
| hsa-let-7a-3p | DPP10-AS2 |
| hsa-miR-1224-5p | RP3-388N13.3 |
| hsa-miR-650 | RP5-892K4.1 |
| hsa-miR-1207-3p | TP73-AS1 |
| hsa-miR-576-5p | CTC-459F4.1 |
| hsa-miR-432-3p | RP5-892K4.1 |
| hsa-miR-2113 | RP11-982M15.8 |
| hsa-miR-101-3p | AC091153.4 |
| hsa-miR-1972 | FAM182A |
| hsa-miR-892a | GAS6-AS1 |
| hsa-miR-125a-3p | FAM182A |
| hsa-miR-15b-3p | AC011284.3 |
| hsa-miR-185-5p | AATBC |
| hsa-miR-432-3p | RP11-333E1.2 |
| hsa-miR-624-3p | AC011718.2 |
| hsa-miR-150-5p | LINC01002 |
| hsa-miR-93-3p | RP5-892K4.1 |
| hsa-miR-186-5p | MIR325HG |
| hsa-miR-650 | AIRN |
| hsa-miR-1224-5p | KB-1183D5.13 |
| hsa-miR-1207-5p | C10orf91 |
| hsa-miR-130a-5p | LL22NC03-27C5.1 |
| hsa-miR-1224-5p | XXyac-YM21GA2.7 |
| hsa-miR-1184 | LINC00689 |
| hsa-miR-1972 | LINC01002 |
| hsa-miR-1207-5p | RP11-618K13.2 |
| hsa-miR-1972 | RP11-1228E12.1 |
| hsa-miR-18a-3p | GAS8-AS1 |
| hsa-miR-342-3p | CTD-3138B18.5 |
| hsa-miR-1224-5p | CTD-2619J13.14 |
| hsa-miR-1236-3p | RP11-717I24.1 |
| hsa-miR-129-5p | RP11-166B2.5 |
| hsa-miR-491-5p | GAS6-AS1 |
| hsa-miR-1972 | LINC01001 |
| hsa-miR-1224-5p | AC011718.2 |
| hsa-miR-1206 | LINC01128 |
| hsa-miR-450b-5p | CTC-265F19.1 |
| hsa-miR-342-3p | RP11-210M15.1 |
| hsa-miR-1972 | LINC00174 |
| hsa-miR-363-5p | MUC2 |
| hsa-miR-1207-5p | LINC00265 |
| hsa-miR-1207-5p | RP11-333E1.2 |
| hsa-miR-150-5p | LINC01165 |
| hsa-miR-198 | RP11-32B5.8 |
| hsa-let-7a-3p | FAM230B |
| hsa-miR-1184 | RP3-470B24.5 |
| hsa-miR-335-3p | SLC8A1-AS1 |
| hsa-miR-499a-3p | RP11-210M15.1 |
| hsa-miR-1207-5p | AP001476.4 |
| hsa-miR-1207-5p | RP4-539M6.22 |
| hsa-miR-1208 | RP11-431K24.1 |
| hsa-miR-650 | RP11-458F8.4 |
| hsa-miR-18a-3p | CTD-2619J13.14 |
| hsa-miR-185-5p | RP11-384K6.6 |
| hsa-miR-499a-3p | FAM182A |
| hsa-miR-1184 | HCG22 |
| hsa-miR-1224-5p | NNT-AS1 |
| hsa-miR-185-5p | FLJ35934 |
| hsa-miR-943 | AP001476.4 |
| hsa-miR-142-3p | MUC2 |
| hsa-miR-1200 | LINC01123 |
| hsa-miR-27a-5p | AP000345.1 |
| hsa-miR-615-5p | COL4A2-AS2 |
| hsa-miR-593-3p | CTD-2532K18.2 |
| hsa-miR-499a-3p | CH507-216K13.2 |
| hsa-miR-18a-3p | NNT-AS1 |
| hsa-miR-1208 | C22orf34 |
| hsa-miR-650 | LINC00689 |
| hsa-miR-1236-3p | LINC00689 |
| hsa-miR-2113 | AC005264.2 |
| hsa-miR-129-5p | AC006548.28 |
| hsa-miR-650 | LINC00265 |
| hsa-miR-145-5p | CTD-3099C6.5 |
| hsa-miR-16-1-3p | CTD-3099C6.5 |
| hsa-miR-363-5p | AC011284.3 |
| hsa-miR-1236-3p | LINC00940 |
| hsa-miR-1206 | LINC01043 |
| hsa-miR-1197 | AC011718.2 |
| hsa-miR-186-5p | LINC00613 |
| hsa-miR-218-5p | RP11-526P6.1 |
| hsa-miR-650 | RP11-304L19.13 |
| hsa-miR-18a-3p | CTD-2517M22.17 |
| hsa-miR-185-5p | RP11-269G24.6 |
| hsa-miR-126-5p | RP11-164O23.8 |
| hsa-miR-650 | RP5-1014D13.2 |
| hsa-miR-27a-3p | RP11-449D8.5 |
| hsa-miR-1229-3p | LL22NC03-27C5.1 |
| hsa-miR-1184 | RP11-1260E13.4 |
| hsa-miR-1200 | CTD-2008P7.3 |
| hsa-miR-1972 | CH507-216K13.2 |
| hsa-miR-539-5p | AC018816.3 |
| hsa-miR-593-3p | AC005330.2 |
| hsa-miR-1184 | AC092535.3 |
| hsa-miR-1207-5p | RP11-680F20.6 |
| hsa-miR-186-5p | DYX1C1-CCPG1 |
| hsa-miR-363-5p | RP11-7M8.2 |
| hsa-miR-1200 | RP11-22M7.2 |
| hsa-miR-767-3p | TMEM191C |
| hsa-miR-1972 | CTD-2330K9.2 |
| hsa-miR-1972 | LINC00905 |
| hsa-miR-1184 | TMEM191A |
| hsa-miR-1207-3p | CTD-2006K23.1 |
| hsa-miR-1207-5p | RP11-867G23.4 |
| hsa-miR-491-5p | CTD-2619J13.14 |
| hsa-miR-18a-3p | ADGRA1-AS1 |
| hsa-miR-650 | RP11-66B24.2 |
| hsa-miR-93-3p | RP11-130L8.1 |
| hsa-miR-767-3p | RP5-902P8.10 |
| hsa-miR-186-5p | RP11-154D6.1 |
| hsa-miR-27a-3p | LINC01123 |
| hsa-miR-185-5p | CTA-280A3.2 |
| hsa-miR-650 | CTD-2013N17.7 |
| hsa-miR-1200 | RP11-627G23.1 |
| hsa-miR-130a-5p | RP11-210M15.1 |
| hsa-miR-125a-3p | LINC00686 |
| hsa-miR-18a-3p | RP5-1029F21.2 |
| hsa-miR-758-3p | AC079586.1 |
| hsa-miR-185-5p | RP11-159D12.10 |
| hsa-miR-186-5p | RP11-22A3.2 |
| hsa-miR-1207-3p | KCNQ1OT1 |
| hsa-miR-590-3p | LINC00240 |
| hsa-miR-185-5p | LINC00265 |
| hsa-miR-186-5p | SFTPD-AS1 |
| hsa-miR-18a-3p | RP11-573D15.2 |
| hsa-miR-130a-5p | AC084219.4 |
| hsa-miR-18a-3p | RP11-469N6.1 |
| hsa-miR-150-5p | AC015849.13 |
| hsa-miR-1207-5p | LINC00969 |
| hsa-miR-650 | RP5-1039K5.19 |
| hsa-miR-593-3p | CTD-2553C6.1 |
| hsa-miR-1207-3p | LINC00588 |
| hsa-miR-593-3p | MZF1-AS1 |
| hsa-miR-615-5p | RP11-32B5.8 |
| hsa-miR-18a-3p | AP001062.7 |
| hsa-miR-16-1-3p | RP11-50B3.4 |
| hsa-miR-1972 | RP11-142C4.6 |
| hsa-miR-129-5p | LINC00662 |
| hsa-miR-146a-3p | LINC00689 |
| hsa-miR-1207-5p | H19 |
| hsa-miR-1207-5p | RP5-1142A6.2 |
| hsa-miR-1200 | RP11-1129I3.1 |
| hsa-miR-15a-3p | RP11-384K6.6 |
| hsa-miR-335-3p | CTA-392E5.1 |
| hsa-miR-590-3p | AC005614.3 |
| hsa-miR-628-5p | RP11-311F12.1 |
| hsa-miR-539-5p | ZNF883 |
| hsa-miR-145-5p | CTA-390C10.9 |
| hsa-miR-198 | CTA-390C10.9 |
| hsa-miR-539-5p | CTC-435M10.10 |
| hsa-miR-150-5p | RP11-38M8.1 |
| hsa-miR-93-3p | CAMTA1-IT1 |
| hsa-miR-1972 | RP11-504P24.8 |
| hsa-miR-18a-3p | CTD-2245F17.9 |
| hsa-miR-1184 | CTD-3193O13.11 |
| hsa-miR-15a-3p | RP11-638I8.1 |
| hsa-miR-129-5p | RP11-69I8.2 |
| hsa-miR-1972 | RP11-849H4.4 |
| hsa-miR-760 | AC006019.3 |
| hsa-miR-1236-3p | RP11-798K23.1 |
| hsa-miR-499a-3p | ZNF883 |
| hsa-miR-29a-3p | RP11-223P11.3 |
| hsa-miR-767-5p | RP11-223P11.3 |
| hsa-miR-650 | RP11-378E13.3 |
| hsa-miR-615-5p | RP11-54O7.17 |
| hsa-miR-1224-5p | CTD-2245F17.9 |
| hsa-miR-1224-5p | LINC00689 |
| hsa-miR-1200 | LINC00689 |
| hsa-miR-650 | CTD-2283N19.1 |
| hsa-miR-15a-3p | RP13-580B18.4 |
| hsa-miR-758-3p | LINC01224 |
| hsa-miR-335-3p | RP11-146D12.2 |
| hsa-miR-335-3p | RP11-335L23.4 |
| hsa-miR-1236-3p | RP11-91K11.2 |
| hsa-miR-34a-3p | CTD-2619J13.19 |
| hsa-miR-760 | RP11-394A14.2 |
| hsa-miR-1184 | RP1-29C18.10 |
| hsa-miR-1236-3p | RP11-23J9.4 |
| hsa-miR-590-3p | RP11-762H8.4 |
| hsa-miR-34a-3p | GS1-279B7.1 |
| hsa-miR-767-3p | CTB-171A8.1 |
| hsa-miR-15a-5p | RP11-483P21.6 |
| hsa-miR-186-5p | RP1-288H2.2 |
| hsa-miR-767-5p | MCF2L-AS1 |
| hsa-miR-27a-3p | CTD-2281E23.1 |
| hsa-miR-1184 | CTD-2311B13.1 |
| hsa-miR-888-5p | AC079799.2 |
| hsa-miR-27a-5p | AC004156.3 |
| hsa-miR-1182 | LINC01128 |
| hsa-miR-342-3p | RP13-580B18.4 |
| hsa-miR-145-5p | RP11-717I24.1 |
| hsa-miR-130a-5p | AC068489.1 |
| hsa-miR-432-5p | RP11-932O9.4 |
| hsa-miR-1207-3p | AC005481.5 |
| hsa-miR-650 | AC074212.5 |
| hsa-miR-650 | CTA-243E7.4 |
| hsa-miR-129-5p | RP11-67K19.3 |
| hsa-miR-186-5p | AC124997.1 |
| hsa-miR-1207-5p | LINC01168 |
| hsa-miR-129-5p | REV3L-IT1 |
| hsa-miR-593-3p | AC137934.1 |
| hsa-miR-32-3p | TCF4-AS2 |
| hsa-miR-590-3p | AC006548.28 |
| hsa-miR-1972 | RP11-1191J2.2 |
| hsa-miR-145-5p | AC015849.16 |
| hsa-miR-491-5p | FAM95B1 |
| hsa-miR-93-3p | CTB-50L17.7 |
| hsa-miR-335-3p | LINC01122 |
| hsa-miR-590-3p | AC093639.1 |
| hsa-miR-1207-5p | AC000095.11 |
| hsa-miR-93-3p | RP11-673P17.2 |
| hsa-miR-650 | EIF3J-AS1 |
| hsa-miR-129-5p | RP5-1125A11.7 |
| hsa-miR-1184 | AC139099.4 |
| hsa-miR-340-5p | LINC00869 |
| hsa-miR-1229-3p | CTD-2562J17.2 |
| hsa-miR-186-5p | CTD-2410N18.4 |
| hsa-miR-539-5p | SATB1-AS1 |
| hsa-miR-129-5p | RP3-508I15.22 |
| hsa-miR-126-5p | RP11-517O13.1 |
| hsa-miR-18a-3p | UCKL1-AS1 |
| hsa-miR-335-3p | RP11-96K19.4 |
| hsa-miR-93-3p | LINC01529 |
| hsa-miR-767-3p | CCDC144NL-AS1 |
| hsa-miR-186-5p | AJ003147.8 |
| hsa-miR-130a-5p | LINC00664 |
| hsa-miR-539-5p | LINC01539 |
| hsa-miR-892a | C22orf34 |
| hsa-miR-1972 | LINC00661 |
| hsa-miR-943 | CTD-3099C6.5 |
| hsa-miR-1184 | COL18A1-AS1 |
| hsa-miR-24-3p | LINC01165 |
| hsa-miR-18a-3p | CTD-2523D13.1 |
| hsa-miR-129-5p | SEPSECS-AS1 |
| hsa-miR-9-5p | RP11-397O4.1 |
| hsa-miR-186-5p | CTD-3046C4.1 |
| hsa-miR-125a-3p | RP11-982M15.8 |
| hsa-miR-1197 | RP11-186N15.3 |
| hsa-miR-650 | RP3-395M20.8 |
| hsa-miR-186-5p | RP11-227H15.4 |
| hsa-miR-1184 | RP3-402G11.28 |
| hsa-miR-125a-3p | LINC00689 |
| hsa-miR-944 | RP5-1077H22.2 |
| hsa-miR-374a-3p | PKD1P6 |
| hsa-miR-1197 | KB-1183D5.13 |
| hsa-miR-185-5p | SSTR5-AS1 |
| hsa-let-7a-3p | LPP-AS2 |
| hsa-miR-539-5p | RP11-598F7.3 |
| hsa-miR-1229-3p | AC012501.2 |
| hsa-miR-129-5p | RP11-848P1.3 |
| hsa-miR-186-5p | LINC00662 |
| hsa-miR-590-3p | CTD-2561J22.5 |
| hsa-miR-129-5p | RP11-486O12.2 |
| hsa-miR-518a-5p | CTD-2521M24.5 |
| hsa-miR-186-5p | CTB-181F24.1 |
| hsa-miR-1207-3p | RP4-751H13.7 |
| hsa-miR-18a-3p | LL22NC03-86G7.1 |
| hsa-miR-129-5p | RP1-283E3.8 |
| hsa-miR-708-3p | LINC00662 |
| hsa-miR-1202 | SNHG14 |
| hsa-miR-758-3p | RP11-157B13.7 |
| hsa-miR-129-5p | RP11-189E14.3 |
| hsa-miR-590-3p | LA16c-60D12.2 |
| hsa-miR-491-5p | RP1-278C19.8 |
| hsa-miR-767-5p | RP4-539M6.22 |
| hsa-miR-125a-3p | CTD-2291D10.2 |
| hsa-miR-758-3p | RP11-15H20.6 |
| hsa-miR-340-5p | RP11-374A4.1 |
| hsa-miR-1972 | FAM95B1 |
| hsa-miR-27a-3p | AC078942.1 |
| hsa-miR-24-3p | AC078942.1 |
| hsa-miR-342-3p | AC078942.1 |
| hsa-miR-185-5p | RP11-458F8.4 |
| hsa-miR-539-5p | EGFLAM-AS3 |
| hsa-miR-650 | LA16c-313D11.12 |
| hsa-miR-1207-5p | MIRLET7BHG |
| hsa-miR-1206 | RP11-13K12.1 |
| hsa-miR-15a-3p | AATBC |
| hsa-miR-491-5p | CTD-2666L21.1 |
| hsa-miR-767-3p | RP11-673P17.2 |
| hsa-miR-186-3p | RP11-368I7.4 |
| hsa-miR-650 | RP5-1171I10.5 |
| hsa-miR-129-5p | RP4-794I6.4 |
| hsa-miR-186-5p | SNHG14 |
| hsa-miR-590-3p | RP11-638L3.1 |
| hsa-miR-574-5p | RP5-894D12.5 |
| hsa-miR-1205 | RP3-470B24.5 |
| hsa-miR-342-3p | LINC01002 |
| hsa-miR-18a-3p | RP11-54O7.17 |
| hsa-miR-15a-5p | RP11-34P13.7 |
